# Changes in the Coding and Non-coding Transcriptome and DNA Methylome that Define the Schwann Cell Repair Phenotype after Nerve Injury

**DOI:** 10.1016/j.celrep.2017.08.064

**Published:** 2017-09-12

**Authors:** Peter J. Arthur-Farraj, Claire C. Morgan, Martyna Adamowicz, Jose A. Gomez-Sanchez, Shaline V. Fazal, Anthony Beucher, Bonnie Razzaghi, Rhona Mirsky, Kristjan R. Jessen, Timothy J. Aitman

**Affiliations:** 1 Department of Clinical Neurosciences, Addenbrooke’s Hospital, University of Cambridge, Cambridge CB2 0QQ, UK; 2 Department of Medicine, Imperial College, London W12 0NN, UK; 3 Department of Cell and Developmental Biology, University College London, London WC1E 6BT, UK; 4 Centre for Genomic and Experimental Medicine, MRC Institute of Genetics and Molecular Medicine, University of Edinburgh, Edinburgh EH16 2XU, UK

**Keywords:** Schwann cell, repair Schwann cell, nerve injury, nerve regeneration, epigenetics, c-Jun, DNA methylation, microRNA, long non-coding RNA

## Abstract

Repair Schwann cells play a critical role in orchestrating nerve repair after injury, but the cellular and molecular processes that generate them are poorly understood. Here, we perform a combined whole-genome, coding and non-coding RNA and CpG methylation study following nerve injury. We show that genes involved in the epithelial-mesenchymal transition are enriched in repair cells, and we identify several long non-coding RNAs in Schwann cells. We demonstrate that the AP-1 transcription factor C-JUN regulates the expression of certain micro RNAs in repair Schwann cells, in particular miR-21 and miR-34. Surprisingly, unlike during development, changes in CpG methylation are limited in injury, restricted to specific locations, such as enhancer regions of Schwann cell-specific genes (e.g., *Nedd4l*), and close to local enrichment of AP-1 motifs. These genetic and epigenomic changes broaden our mechanistic understanding of the formation of repair Schwann cell during peripheral nervous system tissue repair.

## Highlights

• Epithelial-mesenchymal genes are enriched in the injured nerve
• Identification of differentially expressed lncRNAs following nerve injury
• c-Jun regulates specific microRNA expression after nerve injury
• Limited changes in CpG methylation following nerve injury

## Graphical Abstract

**Figure:**
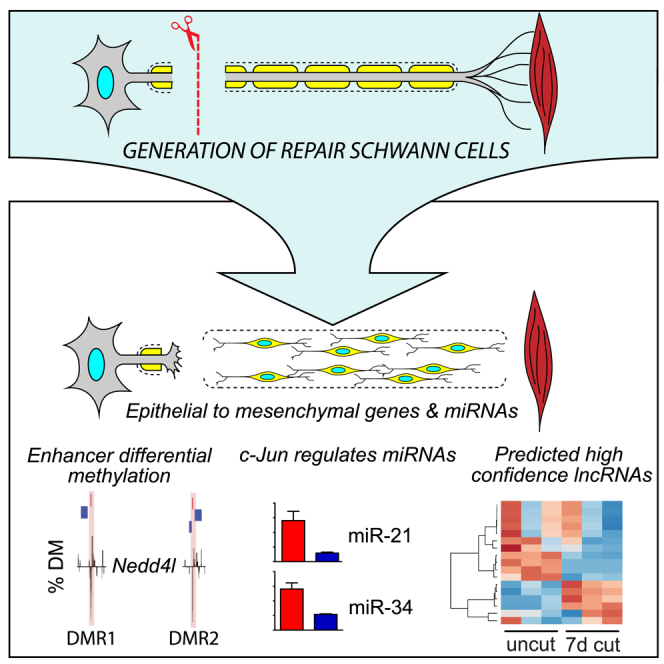

## Teaser

Arthur-Farraj et al. report a combined transcriptome and whole-genome CpG methylation study in repair Schwann cells after nerve injury. They identify Schwann cell-expressed lncRNAs and miRNAs under the control of c-Jun, as well differential methylation of enhancers of repair program genes.

## Introduction

Schwann cells in the peripheral nervous system (PNS) play a crucial role in the repair of injured nerves (Jessen and Mirsky, 2016). In response to injury, the myelin and non-myelin (Remak) Schwann cells that normally ensheath undamaged axons undergo extensive molecular and cellular changes to generate a distinct Schwann cell phenotype, the repair Schwann cell. This cell is specialized for maintaining survival of injured neurons, supports axonal regeneration, and is essential for functional nerve repair. Formation of repair Schwann cells requires the downregulation of genes involved in myelination and upregulation of an injury-specific program of gene expression (Arthur-Farraj et al., 2012, Fontana et al., 2012, Jessen and Mirsky, 2016).

Repair Schwann cells express key regeneration promoting genes, such as neuronal growth factors and cell adhesion molecules; break down redundant myelin sheaths by activating myelin autophagy and recruiting macrophages; and by adopting a slender, elongated morphology, they form regeneration tracts, called bands of Bungner, which help guide axons back to their targets (Gomez-Sanchez et al., 2015, Jessen and Mirsky, 2016). Repair Schwann cell formation is regulated by the AP-1 transcription factor (TF) C-JUN; however, several other factors have subsequently been shown to regulate the Schwann cell response to nerve injury (Arthur-Farraj et al., 2012; reviewed in Boerboom et al., 2017).

The present study explores the involvement of epigenetic mechanisms in the generation of repair Schwann cells after nerve injury, in particular the roles of non-coding RNA and DNA methylation in gene expression and subsequent phenotype (Bonasio et al., 2010). We describe two functional groups of non-coding RNAs, microRNAs (miRNAs), which are 21- to 24-nucleotide regulatory RNAs, and long non-coding RNAs (lncRNAs), which are defined as RNA molecules greater than 200 nucleotides in length with no coding potential (Quinn and Chang, 2016, Sabin et al., 2013). Both miRNAs and global DNA methylation changes have already been shown to have a role in Schwann cell development (Gökbuget et al., 2015, Varela-Rey et al., 2014). After nerve injury, disruption of miRNA processing in Schwann cells results in reduced remyelination in regenerating nerves, but knowledge about the roles or regulation of specific miRNAs is limited (Viader et al., 2011, Zhou et al., 2016). Furthermore, little is known about lncRNA expression and the DNA methylation changes that occur after nerve injury.

Here, we present a large-scale combined study of changes in the coding and non-coding transcriptome and methlyome in response to PNS injury and detail our findings below.

## Results

To determine the changes in the transcriptome and methylome in repair Schwann cells after nerve injury, we performed sciatic nerve cuts in 6- to 8-week-old male C57BL/6J mice and harvested the distal stump for either RNA sequencing (RNA-seq) or whole-genome shotgun bisulfite sequencing (WGSB-seq) 7 days after nerve cut. For miRNA analysis, we also performed small RNA-seq on sciatic nerve distal stump tissue 3 and 7 days after cut. The contralateral uninjured sciatic nerve was the control in all experiments (referred to as uncut throughout this article). Sequencing experiments were performed with a minimum of three biological replicates. Results of RNA-seq, small RNA-seq, and WGSB-seq were confirmed on uncut and cut nerve samples by qPCR and Sanger sequencing, respectively. In order to investigate cell-specific coding and non-coding RNA expression and CpG methylation changes, we also performed qPCR and Sanger sequencing on cultured purified Schwann cells, nerve-derived fibroblasts, and bone-marrow-derived macrophages activated with lipopolysaccharide (LPS).

### RNA-Seq Analysis Shows Enrichment of Genes Involved in Epithelial to Mesenchymal Transition in the Injured Nerve

**Figure 1:**
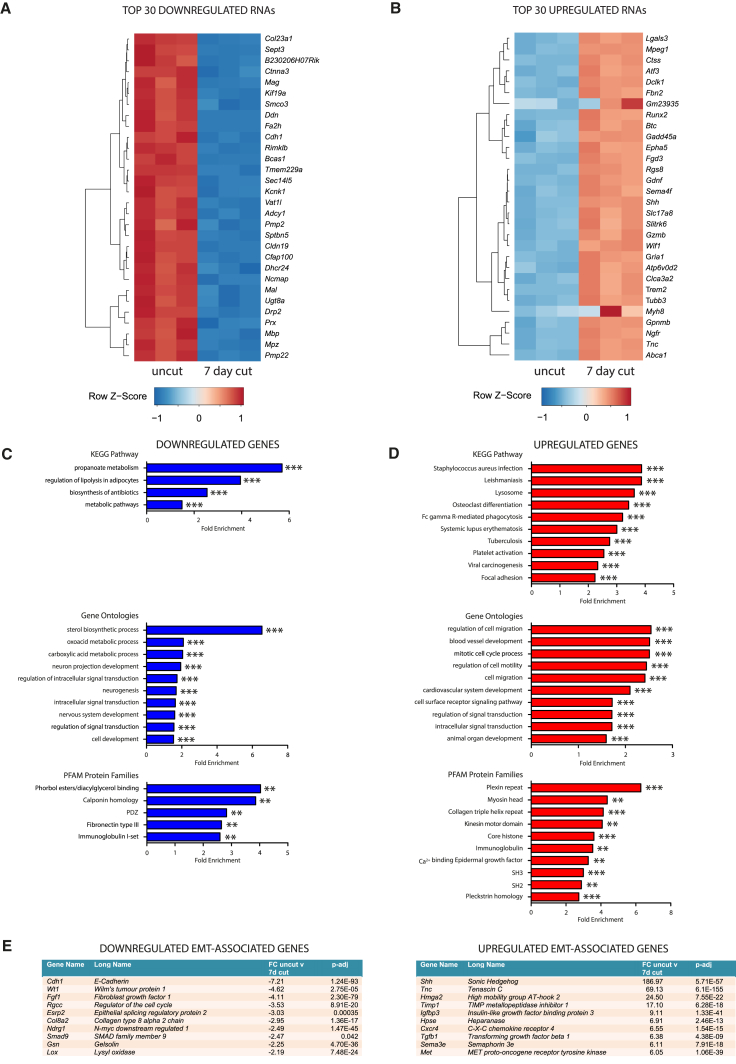
RNA-Seq Analysis Identifies Enrichment of EMT Genes after Nerve Injury (A) A heatmap of the top 30 significantly downregulated genes between uncut and 7-day cut nerves (n = 3, adjusted p value [p-adj] < 0.05). (B) A heatmap of the top 30 significantly upregulated genes between uncut and 7-day cut nerves (n = 3, p-adj < 0.05). (C and D) Enriched KEGG pathways, GO terms, and protein families (PFAM) for (C) downregulated genes and (D) upregulated genes from RNA-seq analysis 7 days after nerve cut compared to the uncut nerve (n = 3). ^∗^p-adj < 0.05; ^∗∗^p-adj < 0.01; ^∗∗∗^p-adj < 0.001. (E) Enrichment analysis of EMT genes from the RNA-seq study showing the 10 most downregulated and upregulated mRNAs (p-adj < 0.05).

From our RNA-seq analysis, we identified 3,176 differentially expressed (DE) coding and non-coding RNAs between uncut and 7-day cut mouse sciatic nerves (Table S1; Figures S1A and S1B). These results correlated well with our previously published microarray data (Arthur-Farraj et al., 2012), where 80% of significantly DE genes (fold change >2, p-adj < 0.05) from the microarray were also significant and in the same direction in our RNA-seq dataset (see Supplemental Experimental Procedures). Among the top 30 most downregulated RNAs, reassuringly, we identified a number of known myelin-associated genes, such as *Mbp*, *Pmp22*, *Mpz*, *Prx*, *Drp2*, and *Cdh1* (Figure 1A). Similarly, among the top 30 most upregulated RNAs 7 days after nerve injury were several well-known repair program genes, such as *Ngfr*, *Lgals3*, *Atf3*, *Shh*, and *Gdnf* (Arthur-Farraj et al., 2012; Figure 1B). Out of all DE RNAs, we selected mainly upregulated RNAs to validate by qPCR based on their potential roles in repair cells identified from literature searches and Kyoto Encyclopedia of Genes and Genomes (KEGG) pathway and protein family analysis (Figures 1C and 1D; Table S2A). Myelin genes and known repair program genes were used as controls. In total, we successfully validated 36 out of these 37 RNAs by qPCR on uncut and 7-day cut nerves. These included the main AP-1 TF members, four lncRNAs, and repair cell genes with potential roles in extracellular matrix (ECM) remodeling, axon growth and intracellular signaling (Table S2A). Although the majority of cells in uninjured and injured nerves are Schwann cells (Table S2C), we wanted to check the relative expression of putative repair program RNAs in the major different cells types found within the injured nerve. As cultured Schwann cells closely replicate the gene expression of repair Schwann cells in vivo, they make a valid in vitro assay for repair cells (Arthur-Farraj et al., 2012). Using purified cultures of Schwann cells, nerve fibroblasts, and macrophages, we found that the large majority of putative repair program coding and non-coding RNAs (24 out of 33) we tested were significantly more highly expressed in Schwann cells than in fibroblasts or macrophages (Table S2B).

To observe the broader regulation of genes after nerve injury, we performed KEGG pathway, gene ontology (GO), and protein family enrichment analysis (PFAM). Among downregulated genes, this revealed enrichment in the sterol biosynthetic processes and regulation of lipolysis and significant enrichment for genes belonging to diacylglycerol-binding, calponin homology, and PDZ-domain-containing protein families (Figure 1C). Within upregulated genes, there was enrichment of lysosome, osteoclast differentiation, focal adhesion, and inflammatory pathways along with biological processes involving the cell cycle, migration, signal transduction, and cell surface receptor signaling. Furthermore, there was enrichment of genes belonging to various protein families, including collagens, plexins, myosins, and calcium-binding epidermal growth factor domains (Figure 1D). These findings emphasize the importance of gene expression changes after nerve injury in remodeling of the ECM, changes in the actin cytoskeleton, and changes in intracellular signaling in repair Schwann cells.

The morphological transition from a differentiated Schwann cell into a repair Schwann cell is similar to epithelial-mesenchymal transition (EMT), which has well-known roles in oncogenesis and wound healing (Lamouille et al., 2014). Myelin Schwann cells have previously been likened to epithelial cells (Bunge and Bunge, 1983), as they have a basement membrane and cell polarity, with an abaxonal and adaxonal membrane, and express typical epithelial markers such as E-cadherin, claudins, and polarity proteins such as PAR3 (Chan et al., 2006, Crawford et al., 2008; Table S1). After nerve injury, Schwann cells lose their abaxonal and adaxonal polarity, re-enter the cell cycle, and adopt migratory behavior at the site of injury (Jessen and Mirsky, 2016).

To test whether genes involved in EMT were substantially regulated after nerve injury, we utilized the list of known human EMT genes in the dbEMT database and identified the corresponding mouse orthologs (Zhao et al., 2015). Out of the 326 mouse-to-human orthologous EMT genes, 111 were significantly DE between uncut and 7-day cut nerves, and a Fisher’s exact test confirmed enrichment (p < 2.2E−16; odds ratio [OR], 3.14; 95% confidence interval [CI], 2.46, 3.97; Table S3). Of the top 10 most upregulated EMT genes, there were mRNAs known to be specifically expressed in repair Schwann cells, such as *Shh*, *Tnc*, and *Tgfb1* (Arthur-Farraj et al., 2012, Martini et al., 1990, Scherer et al., 1993; Figure 1E; Table S3). Furthermore, we also found that there was significant enrichment of EMT genes within the subset of Schwann cell/*c-Jun*-dependent genes identified from our previous microarray (11/172 genes; OR, 14.58; p = 1.39E−09; Arthur-Farraj et al., 2012). These results show that a significant proportion of the molecular machinery involved in EMT is also regulated during the formation of repair Schwann cells after nerve injury.

### Fast Activation of AP-1 TFs in Repair Schwann Cells

**Figure 2:**
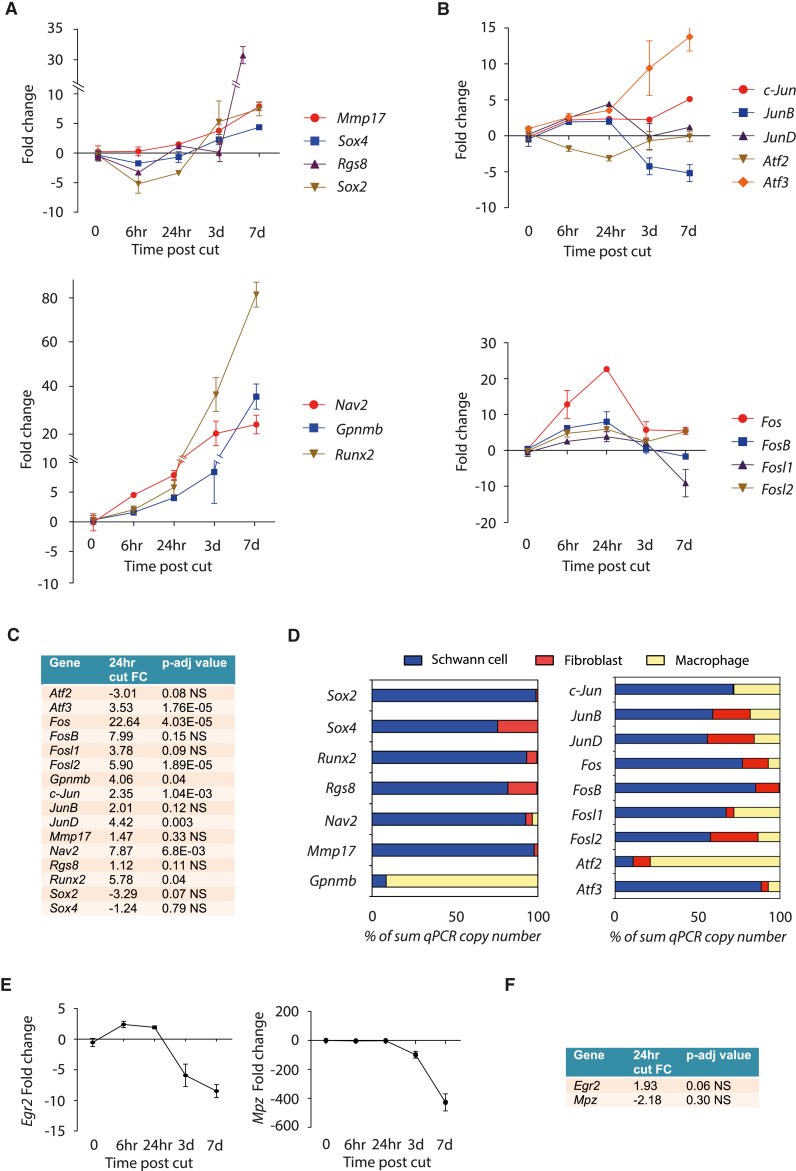
Expression Patterns for Putative Schwann Cell Repair Program Genes and AP-1 TFs after Nerve Injury (A) Time course of putative repair program gene expression after sciatic nerve cut by qPCR (n = 5). (B) Time course of expression of members of the AP-1 TF family after sciatic nerve cut by qPCR (n = 5). Fold change in (A) and (B) is relative to the uncut nerve. (C) Table showing fold change values of repair program genes and all AP-1 TFs 24 hr post-cut relative to uncut nerve. p-adj values are displayed. (D) Relative cell type expression of repair program genes and AP-1 TFs in cultured mouse Schwann cells (blue), nerve fibroblasts (red), and activated macrophages (yellow) displayed as percentage of the sum of qPCR copy-number values from 1ug of RNA from each of the three cell types (n = 3). (E) Time course of expression of *Egr2* and *Mpz* by qPCR after sciatic nerve cut. Fold change is relative to the uncut nerve (n = 5). (F) Table showing fold change values of myelin program genes, *Egr2* and *Mpz*, 24 hr post-cut relative to uncut nerve, with p-adj displayed. Error bars in all graphs represent SEM.

*c-Jun* mRNA and protein are upregulated within the first 24 hr after nerve injury, and it has been suggested that this fast rate of upregulation may be important for the strong effects of Schwann cell C-JUN on nerve regeneration (Arthur-Farraj et al., 2012, Painter et al., 2014, Parkinson et al., 2008). We determined the time course of expression for a number of repair program genes that we validated from the RNA-seq results, including *c-Jun* and other members of the AP-1 TF family. There was little change or reduction in the expression of several repair program genes, such as *Rgs8*, *Sox2*, and *Sox4*, during the first 3 days after cut, followed by a linear increase up to day 7 (Figure 2A). However, other repair program genes, such as *Nav2* and *Runx2*, and many of the AP-1 genes followed a different expression pattern, showing an early peak of mRNA expression ∼24 hr after nerve injury (Figures 2A–2C). Importantly, 20 out of 22 genes examined in this set of experiments were significantly more highly expressed in cultured Schwann cells than in nerve fibroblasts or macrophages. Only *JunB* and *Gpnmb*, while expressed in Schwann cells, were more highly expressed in macrophages (Figure 2D; Table S2B).

Interestingly, the fast activation of repair program genes was not accompanied by downregulation of myelin genes, as both *Mpz* and *Egr2* mRNA were still robustly expressed at 24 hr post-cut and only significantly downregulated at 3 days post-cut (Figures 2E and 2F). This indicates that transcriptional activation of the repair program starts very soon after injury, before the TFs that control myelination are extinguished and, crucially, before axonal degeneration has taken place, which happens at 36–44 hr in mouse sciatic nerves (Conforti et al., 2014). Therefore, the transcriptional regulators of both the myelin program and the repair program may be active in Schwann cells at early time points after nerve injury.

### Expression Patterns of Non-coding RNAs in Repair Schwann Cells

**Figure 3:**
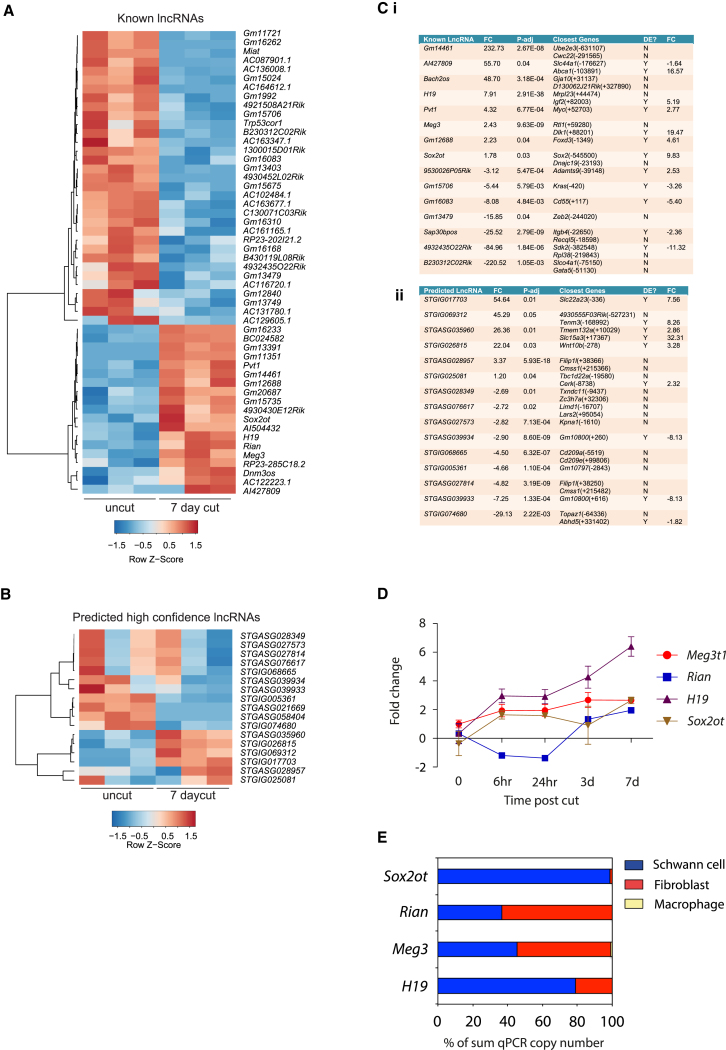
lncRNA Expression after Nerve Injury (A) A heatmap of the 52 annotated DE lncRNAs between uncut and 7-day cut sciatic nerve from RNA-seq experiments (p-adj < 0.05). (B) A heatmap of the 17 predicted DE lncRNAs between uncut and 7-day cut sciatic nerve from RNA-seq experiments (p-adj < 0.05). (C) Table of selected known (i) and predicted (ii) lncRNAs, with fold change (FC) and p-adj values from the RNA-seq study along with their closest genes, whether they are also differentially expressed (DE?) in the RNA-seq study, and the corresponding fold change. (D) Time course of expression of the lncRNAs, *Meg3* (t1 = transcript 1), *Rian*, *H19*, and *Sox2ot* after sciatic nerve cut by qPCR (n = 4). Fold change is relative to the uncut nerve. Error bars represent SEM. (E) Relative cell type expression of *Meg3* (transcript 1), *Rian*, *H19*, and *Sox2ot* in cultured mouse Schwann cells (blue), nerve fibroblasts (red), and activated macrophages (yellow) displayed as percentage of the sum of qPCR copy-number values from 1ug of RNA from each of the three cell types (n = 3).

Between uncut and 7-day cut samples, we identified 52 known DE lncRNAs out of a total of 1,533 annotated lncRNAs. Of these 52, 19 were upregulated and 33 were downregulated (Figure 3A; Table S1). Additionally, we identified 913 predicted high-confidence lncRNAs, 433 antisense and 480 intergenic, of which 17 were DE (11 up and 6 down), 7 days after nerve cut (Figures 3B and S2; Table S1). Interestingly, a number of known and predicted lncRNAs were located near genes that were also DE after nerve injury. These included *H19* and *Igf2*, *Pvt1* and *Myc*, *Gm12688* and *Foxd3*, *Sox2ot* and *Sox2*, *GM16083* and *Cd55*, *Sap30bpos* and *Itgb4*, *STGIG035960* and *Slc15a3*, and *STGIG026815* and *Wnt10b* (Figure 3C). We validated expression of four lncRNAs upregulated after nerve injury by qPCR (Figure 3D; Table S2A). We then used cell culture to examine the cell-type expression of these lncRNAs and found that *Sox2ot* and *H19* were both preferentially expressed in Schwann cells. *Rian* and *Meg3* were also expressed in Schwann cells, although they were found at higher levels in nerve fibroblasts. There was negligible expression of these four lncRNAs in macrophages (Figure 3E; Table S2B).

**Figure 4:**
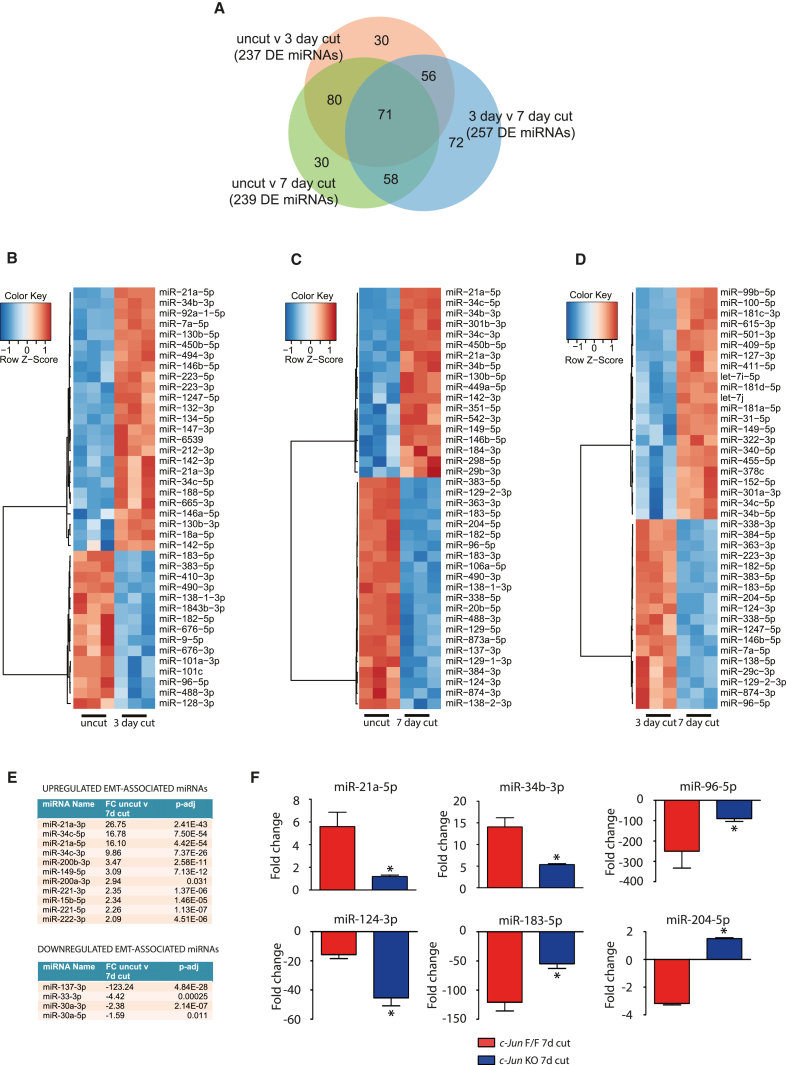
miRNA Expression after Nerve Injury (A) Venn diagram showing the overlap of DE miRNAs between uncut, 3-day cut, and 7-day cut sciatic nerve. (B–D) Heatmap of the top 40 DE miRNAs between (B) uncut and 3-day cut nerves, (C) uncut and 7-day cut nerves, and (D) 3-day cut and 7-day cut nerves (n = 5, p-adj < 0.05). (E) Enrichment analysis of EMT associated miRNAs from small RNA-seq study showing the most upregulated and downregulated miRNAs (FC, Fold change; p-adj < 0.05). (F) Expression of miRNAs in *c-Jun* flox/flox (control; red) and *c-Jun* null (*P0Cre c-Jun* flox/flox; blue) 7-day cut nerves. ^∗^p < 0.05, n = 3. Fold change is relative to the uncut control nerve (*c-Jun* flox/flox). Error bars represent SEM.

We identified a total of 397 DE miRNAs between uncut and cut samples from our small RNA-seq analysis. We analyzed a 3-day time point, in addition to 7 days post-cut, as earlier time points had previously been shown to have significant miRNA regulation (Viader et al., 2011, Adilakshmi et al., 2012) (Figure 4A; Table S4). We found similar numbers of DE miRNAs at 3 days and 7 days post-cut when both were compared to uncut nerve. Specifically, we found 237 DE miRNAs between uncut and 3-day cut samples, of which 113 were upregulated and 124 were downregulated. Between uncut and 7-day cut samples, we identified 239 DE miRNAs, of which 114 were upregulated and 125 were downregulated. Comparisons between 3-day and 7-day cut samples revealed 257 DE miRNAs, with 127 significantly upregulated and 130 downregulated (Figures 4B–4D; Table S4). Six miRNAs (miR-146b, miR-383-5p, miR-34c-5p, miR-96-5p, miR-183-5p, and miR-182-5p) were among the top 40 regulated miRNAs in all three comparisons, and miR-21a-5p and miR-34b-3p were among the top three most upregulated miRNAs in 3-day and 7-day cut nerves (Figures 4B–4D; Table S4). We successfully validated 15 miRNAs (7 upregulated and 8 downregulated) on uncut and 3-day and 7-day cut samples using qPCR (Figures S3A and S3B).

To test whether miRNAs involved in EMT were regulated after nerve cut, we again used the dbEMT database to obtain a list of known human EMT miRNAs and identified the corresponding mouse orthologs (Zhao et al., 2015). We found a significant enrichment of miRNAs involved in EMT. 15 out of 20 mouse orthologous miRNAs with a role in EMT were DE between uncut and 7-day cut samples from our small RNA-seq analysis; this included several members of the miR-21 and miR-34 families (p < 0.013; OR, 2.61; 95% CI, 1.15, 5.94; Figure 4E; Table S3).

Six of the seven validated upregulated miRNAs after nerve injury were expressed in cultured Schwann cells (Figure S3C). miR-17-5p and miR-362-3p showed the highest relative expression in Schwann cells, while miR-142-3p was expressed specifically in cultured macrophages. However, expression levels of miR-21-3p, miR-34a-5p, miR-34b-5p, miR34c-5p, and miR-132-3p were higher in nerve fibroblasts than in Schwann cells (Figure S3C).

Using the miRWalk webserver, we identified a set of miRNAs that target genes associated with some of the enriched KEGG pathways, gene ontologies, and protein families from our RNA-seq study (Figures 1 and S3D). For instance, two miRNAs belonging to the miR29 family (miR-29a and miR-29c) and two members of the miR-154 family (miR-409 and miR-494) were both predicted to target genes enriched for the focal adhesion pathway, and miR29 family members are also predicted to target collagen protein families (Figure S3D).

These findings demonstrate that there is significant regulation of non-coding RNAs in the injured peripheral nerve. In particular, the lncRNAs *H19* and *Sox2ot* are highly expressed in cultured Schwann cells. However, we have also identified that a number of lncRNAs and miRNAs are also highly expressed in nerve fibroblasts. This suggests that other cells types, in addition to Schwann cells, are likely to contribute to non-coding RNA expression patterns in the injured nerve.

### *c-Jun* Regulates the Expression of miRNA in Repair Schwann Cells

In silico interactions among miRNAs, mRNAs, and TFs were calculated using six independent predictor methods. In this way, we identified *c-Jun* and *Foxd3* as potential key regulators of miRNA expression (Figure S3E). We analyzed the expression of the 15 validated injury-regulated miRNAs in cut nerves of a Schwann cell-specific knockout of *c-Jun* (Arthur-Farraj et al., 2012). We found no significant differences in expression of four miRNAs, which were predicted to be regulated by *c-Jun*, between cut *c-Jun flox/flox* control nerves and *c-Jun*-null nerves (Figure S2F). However, we did identify six miRNAs that were DE between 7-day cut control and *c-Jun* null nerves. Both miR-21a-5p and miR-34b were expressed at significantly lower levels in *c-Jun*-null cut nerves than in controls, suggesting that C-JUN may be important for their upregulation after nerve injury. In contrast, miR96-5p, miR-124-3p, miR-183-5p, and miR-204-5p were more highly expressed in *c-Jun*-null cut nerves than in controls, indicating that C-JUN may downregulate expression of these miRNAs after nerve injury (Figure 4F). These results show that the *c-Jun*-dependent control of Schwann cell reprogramming extends to the regulation of appropriate miRNA levels in injured nerves.

### CpG Methylation Changes in Repair Schwann Cells after Nerve Injury

**Figure 5:**
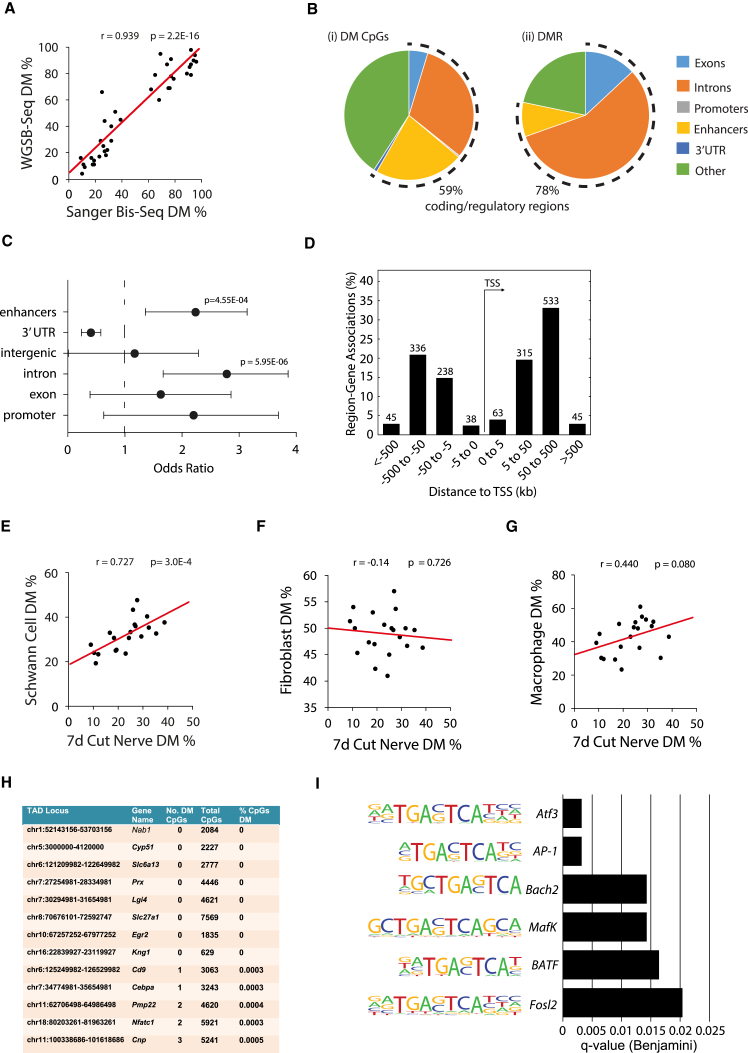
Overview of the Methylome in the Injured Nerve (A) Validation of WGSB-seq results by Sanger sequencing of bisulfite-treated DNA. Strong correlation of total methylation percentage of 20 individual CpGs within six DMRs in WGSB-seq compared with Sanger sequencing. DMRs related to genes *Cln8*, *Mob3b*, *Arl4C*, and *Nr1h4* and two DMRs in *Nedd4l* were used. (B) Percentage of DM CpGs (i) or DMRs(ii) in coding/regulatory regions (dotted line) or other genomic loci in 7-day cut nerve. (C) Enrichment of DM CpGs across genomic loci with odds ratio and confidence intervals (x axis), genomic loci (y axis), and significant p-adj values shown. Error bars represent SEM. (D) Distance of DM CpGs to the transcription start site (TSS), binned by distance and gene orientation, with total number of DM CpGs shown above each bin. (E–G) Percentage total methylation of 20 individual CpGs within six DMRs by Sanger sequencing in 7-day cut nerves compared with cultured (E) mouse Schwann cells, (F) nerve fibroblasts, and (G) activated macrophages (n = 3, ^∗^p-adj < 0.05). (H) Table demonstrating lack of DM around myelin genes after nerve injury. For each topological associated domain (TAD) locus containing a known myelin gene, the percentage of DM CpGs is shown. (I) Enrichment of significant transcription-factor-binding motifs in close proximity to DM CpGs.

For analysis of CpG methylation changes in injured nerves, we performed WGSB-seq. First, we identified 4,221,750 methylated CpG sites that had >5× coverage (meaning that each CpG site was sequenced at least five times) across the genome. Validation of the WGBS-seq data was obtained by locus-specific bisulfite-Sanger sequencing (r^2^ = 0.88) at 20 individual CpGs (Figures 5A and S4A–S4C). Out of these 4,221,750 CpGs, only 853 were significantly differentially methylated (DM) between uncut and 7-day cut samples (adjusted p value [p-adj] < 0.05; differential methylation difference >20%) (Table S5; Figure S4D). Since biologically significant alterations in CpG methylation often affect adjacent CpGs, we clustered together ≥2 DM CpGs within 500 bp of one another and identified 46 unique differentially methylated regions (DMR) (Figure S4E). Significant enrichment of individual DM CpGs and DMRs occurred within gene regulatory regions, in particular introns and enhancers (Figures 5B, 5C, and S4D–S4F). Importantly, this enrichment was independent of technical variation in CpG read coverage (Figure S4G). DM CpGs were predominantly within 500 kb of transcription start sites (TSSs), indicating their *cis*-regulatory potential (Figure 5D). Next, we investigated whether the DM in the nerve is a result of Schwann cell-specific changes. We assessed percentage total methylation at individual CpGs in six of the most DM CpG clusters, identified from our WGSB-seq analysis. We then compared this to the percentage methylation of the same CpGs in cultured Schwann cells, nerve-derived fibroblasts, and activated macrophages using Sanger sequencing. We found CpG methylation levels in Schwann cells correlated strongly with levels of CpG methylation in the injured nerve, whereas no significant correlation was found for macrophages and fibroblasts with injured nerve samples (Figures 5E–5G).

The expression of myelin genes is reduced in adulthood relative to that seen during active myelination in developing nerves, and this reduction is accompanied by increased methylation of promoters and enhancer regions of myelin genes (Varela-Rey et al., 2014). Downregulation of myelin genes also takes place after injury, when adult expression levels decline to the very low levels seen in repair Schwann cells. However, comparing uncut nerves with 7-day cut nerves, we observed barely any CpG (between 0.0003% and 0.0005%) DM in putative regulatory regions within myelin-associated gene boundaries (determined by topological associated domains; see Supplemental Experimental Procedures) (Figure 5H). Surprisingly, therefore, myelin gene downregulation associated with development and after injury appear to be regulated differently, with CpG methylation having a reduced role in the injury response.

CpG methylation has previously been suggested to influence TF binding to target sites in DNA (Domcke et al., 2015, Schmidl et al., 2009). To investigate whether DM was occurring in or around specific TF-binding sites, we performed a de novo motif search within a 40-bp window centered on each of the 853 DM CpGs. In particular, we identified AP-1, *Atf3*, and *Fosl2* motifs as significantly enriched (Figure 5I). Both *Atf3* and *Fosl2* are AP-1 family members, along with *c-Jun*. These results demonstrate that significant changes in DNA methylation occur near TF-binding motifs for *c-Jun* and other AP-1 members that are expressed as part of the repair Schwann cell phenotype.

### Differential CpG Methylation in Enhancers of Repair Program Genes

**Figure 6:**
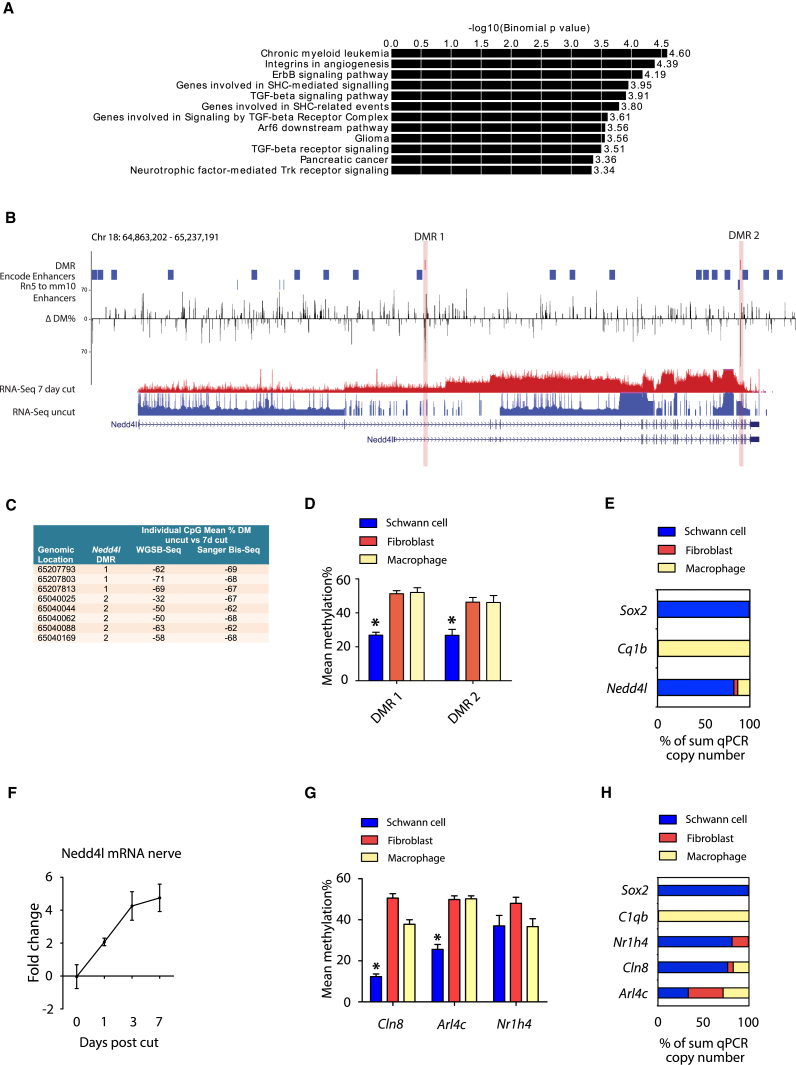
Enhancer-Specific Differential Methylation in the Injured Nerve (A) Pathway enrichment of genes associated with DM enhancer loci. (B) Differential CpG methylation after 7-day nerve cut compared to uncut nerve along the *Nedd4l* gene. The two DMRs, both of which were significantly demethylated after nerve cut, are marked in red, and mouse (mm10) encode enhancers and active rat (rn5) nerve enhancers, identified by Hung et al., (2015), are shown. DMR2 is located in close proximity to an active rat injured nerve enhancer. Mean RNA-seq data for uncut (blue) and 7-day cut (red) nerves are shown in addition to the location of all introns (arrowed blue lines) and exons (vertical blue lines) within the *Nedd4l* gene. (C) Genomic location and mean DM between uncut and 7-day cut nerves of individual CpGs within both DMRs of the *Nedd4l* gene measured by WGSB-seq and by bisulfite Sanger sequencing. (D) Mean methylation percentage for the two DMRs within *Nedd4l* enhancers in cultured mouse Schwann cells (blue), nerve fibroblasts (red), and activated macrophages (yellow). ^∗^p-adj < 0.05, n = 3. (E) Relative cell type expression of *Nedd4l* mRNA in cultured mouse Schwann cells (blue), nerve fibroblasts (red), and activated macrophages (yellow) displayed as percentage of the sum of qPCR copy-number values from 1 μg RNA from each of the three cell types (n = 3). *Sox2* and *Cq1b* are included as positive controls for Schwann cells and macrophages, respectively. (F) Time course of *Nedd4l* mRNA expression in the distal segment after sciatic nerve cut (n = 4). (G) Mean methylation percentage for DMRs near *Arl4c*, *Cln8*, and *Nr1h4* genes in cultured mouse Schwann cells (blue), nerve fibroblasts (red), and activated macrophages (yellow). ^∗^p-adj < 0.05. (H) Relative cell-type expression of *Arl4c*, *Cln8*, and *Nr1h4* mRNA in cultured mouse Schwann cells (blue), nerve fibroblasts (red), and activated macrophages (yellow) displayed as percentage of the sum of qPCR copy-number values from 1 μg RNA from each of the three cell types (n = 3). *Sox2* and *Cq1b* are included as positive controls for Schwann cells and macrophages, respectively. Error bars in all graphs represent SEM.

We correlated WGSB-seq data with gene expression data from the RNA-seq experiments (Tables S1 and S5) 7 days after nerve cut. No correlation with gene expression was observed between DM CpGs found within exons, introns, intergenic regions, or enhancers, and weak negative correlation was observed with DM in the promoter region based on a limited number of CpGs (Figure S5A). Of the DM CpGs that occurred in enhancers, we found enrichment of genes associated in particular with ERBB, transforming growth factor β (TGF-β), and neurotrophin signaling (Figure 6A). Enhancers are tissue-specific regulatory elements that exhibit low levels of sequence conservation across vertebrates (Villar et al., 2015). While histone marks of enhancer activity have not been studied in injured mouse nerves, a recent study identified H3K27acylation chromatin immunoprecipitation sequencing (ChIP-seq) peaks in injured rat nerve (Hung et al., 2015), and we mapped 2,529 of these rat enhancers to mm10 mouse genome coordinates. Despite the majority of the mouse DM CpGs occurring >1,000 bp away from the mapped enhancers, 18 DM CpGs either overlapped or were in syntenic conservation with the mapped sciatic nerve enhancers, which were associated with 23 unique genes, including *Nedd4l*, *Arl4c*, *Filip1*, and *Camk2d* (Table S5).

We identified two DMRs, within intronic regions of *Nedd4l*, which contained three DM CpGs within 22 bp and five DM CpGs within 146 bp. These DMRs had a mean decrease in CpG methylation of 66.9% and 49.9%, respectively, which we also validated by Sanger bisulfite sequencing (Figures 6B, 6C, and S5B). The second DMR was located within a known mouse enhancer region and in close proximity to a mapped rat sciatic nerve enhancer (Figure 6B). Sanger bisulfite sequencing showed that these two *Nedd4l*-associated DMRs are significantly hypomethylated in cultured Schwann cells relative to macrophages and fibroblasts (Figure 6D). We also confirmed *Nedd4l* upregulation in injured sciatic nerve by qPCR and demonstrated that *Nedd4l* mRNA is more highly expressed in cultured Schwann cells than in macrophages and nerve fibroblasts (Figures 6E and 6F). In addition to *Nedd4l*, we found relative hypomethylation of a DMR in the *Cln8* gene along with strong expression of *Cln8* mRNA in cultured Schwann cells when compared to cultured macrophages and fibroblasts (Figures 6G and 6H; Tables S2A and S2B).

In summary, these findings indicate that Schwann cell-specific CpG methylation changes occur within putative enhancer regions of the genome and are correlated with changes in expression of their nearest gene, such as in the case for *Nedd4l* and *Cln8*.

## Discussion

The aims of the present study were to characterize more fully the genetic and epigenetic signature of the repair Schwann cell, define further how the phenotype of this cell depends on c-Jun, and determine the DNA methylation changes that accompany the conversion of myelin and Remak Schwann cells to repair Schwann cells in injured nerves.

### AP-1 TF Expression in Repair Schwann Cells

C-JUN has to homo- or heterodimerize with itself or another AP-1 family member to form transcriptionally active complexes (Wagner, 2002); however, the binding partner for C-JUN in repair Schwann cells is unknown. We find that both *Fosl2* and *Atf3* show expression profiles similar to *c-Jun*, and along with *Fos*, they are the main AP-1 family members highly expressed at the mRNA level 7 days after nerve cut. Interestingly, *c-Jun*, *JunD*, *Fos*, *Fosl2*, and *Atf3* were all significantly elevated only 24 hr after nerve injury, as were other repair-program genes, such as *Nav2* and *Runx2*. At this time point, myelin genes such as *Egr2* and *Mpz* are still highly expressed and axonal degeneration has not yet taken place (Conforti et al., 2014). This suggests that at early time points after nerve injury, Schwann cells may express transcriptional components of both the repair program and the myelin program. In further support of this, phosphorylation of the ERBB2 receptor within 1 hr and the p38 mitogen-activated protein kinase (MAPK) and extracellular signal-related kinase (ERK) pathways within 6–24 hr is observed in Schwann cells after nerve injury (Guertin et al., 2005, Harrisingh et al., 2004, Yang et al., 2012). Additionally, a recent study showed that there is loss of the repressive histone mark H3K27me3 at the promoter and gene bodies of a number of repair program genes such as *Shh*, *Gdnf*, and *Fgf5* within 24 hr of nerve injury in the rat (Ma et al., 2016). Together, these findings support the view that Schwann cells may in part be able to sense nerve injury before axonal degeneration has taken place.

### Repair Schwann Cell Formation Involves EMT

Repair Schwann cell formation represents an adaptive response of an adult differentiated cell type to tissue injury (Jessen et al., 2015). The transition from a myelinated Schwann cell to a repair Schwann cell shares some similarities with EMT. Using a database of all mRNAs and miRNAs currently known to have a role in EMT, we have shown that these genes are markedly enriched in our datasets from injured nerve samples from both wild-type and Schwann cell-specific *c-Jun*-knockout mice (Arthur-Farraj et al., 2012, Zhao et al., 2015). A number of these genes have already been shown to have roles in repair Schwann cells, including *Notch1*, *Sonic Hedgehog*, and members of the miR-34, miR-221, and miR-222 families (Martinez et al., 2015, Viader et al., 2011, Woodhoo et al., 2009, Zhou et al., 2016). Additionally, repair Schwann cells also demonstrate nuclear translocation of β-catenin, a key step in EMT, and express a number of genes expressed by mesenchymal tissue such as *α5 integrin* and *vimentin* and, at the protein level, N-cadherin and neural cell adhesion molecule (Arthur-Farraj et al., 2012, Jung et al., 2011, Lamouille et al., 2014; Table S1).

EMT has a well-established role in tissue remodeling, repair, and fibrosis (Thiery et al., 2009). Tissue injury in organs, such as the kidney and intestine, results in mesenchymal-like cell formation through EMT to promote tissue repair. This process requires a prominent inflammatory response to drive the process, which is another similarity shared with nerve injury (Thiery et al., 2009). The similarities between repair Schwann cell generation on the one hand and EMT on the other are therefore likely to reflect the relationship between this event and injury responses in other tissues. EMT is an important mechanism in tumor formation and invasiveness, and in repair Schwann cells, the involvement of this process may also relate to their capacity to form malignant nerve sheath tumors. Further investigation of the molecular mechanism that underlies the repair Schwann cell transition may therefore potentially benefit both the fields of regenerative medicine and cancer biology.

### Non-coding RNA Expression in Repair Schwann Cells

The role of lncRNAs in the regulation of gene expression is incompletely understood, but they have broadly described roles in regulating transcription, translation, and chromatin remodeling (Quinn and Chang, 2016). After nerve injury, we identified 52 annotated and 17 predicted high-confidence DE lncRNAs. A recent study found 3,314 DE lncRNAs in mouse 7-day crushed nerves (Pan et al., 2017). The disparity between their findings and ours are influenced by (1) the use of a nerve crush versus nerve cut model, (2) parametric tests to determine differential expression versus utilization of the negative binomial distribution, (3) less stringent cutoffs for lowly expressed lncRNAs, and (4) not including FANTOM5 cap analysis of gene expression (CAGE) data to limit lncRNAs to those with defined 5′ ends (Hon et al., 2017).

We found that both *Sox2* overlapping transcript (*Sox2ot*) and *H19* were highly expressed in cultured Schwann cells. *Sox2ot* is a multi-exon lncRNA that contains the *Sox2* gene within one of its introns. *Sox2* is strongly expressed in repair Schwann cells and may have a role in cell-to-cell contact (Parrinello et al., 2010). Interestingly, *Sox2ot* has been shown to positively regulate the expression of *Sox2* in a breast cancer cell line (Askarian-Amiri et al., 2014), suggesting that it may be a regulator of *Sox2* expression in repair Schwann cells too. *H19* is a maternally expressed, paternally imprinted gene, which resides in close proximity to the *Igf2* gene, which is reciprocally imprinted and paternally expressed. *H19* has been shown to regulate osteogenesis, partly through modulation of *Runx2*-dependent gene expression, which is a TF that is also highly expressed in repair Schwann cells (Huang et al., 2015).

Our results have greatly expanded the list of known miRNAs regulated after nerve injury (Adilakshmi et al., 2012, Viader et al., 2011, Zhou et al., 2016). Using six independent predictor methods, we revealed the TFs *c-Jun* and *Foxd3* as potential key regulators of a number of miRNAs after nerve injury. Through analysis of a Schwann cell-specific knockout of *c-Jun*, we found six dysregulated miRNAs out of 15 tested 7 days after nerve cut. One of the most significantly regulated miRNAs, miR-21, has been shown to be a direct target of C-JUN in vascular endothelial cells (Zhou et al., 2011), and we found that miR-21-3p is expressed at much lower levels in *c-Jun*-null injured nerves than in control injured nerves. The function of miR-21 in Schwann cells is unknown, but it has been shown to target Sox2 during differentiation of hair-follicle-derived neural crest stem cells into Schwann cells (Ni et al., 2014).

### The Role of CpG Methylation after Nerve Injury

Roles for cytosine methylation have been implicated in cell differentiation, particularly in the hematopoietic system, Schwann cell myelination, and other processes such as tumorigenesis, splicing, and X chromosome inactivation (Beerman et al., 2013, Jones, 2012, Varela-Rey et al., 2014). In the PNS, mutations in DNA methyltransferase 1 cause a form of hereditary sensory and autonomic neuropathy through aberrant DNA methylation (Klein et al., 2011). Furthermore, methylome analysis of neuronal cell bodies in dorsal root ganglia (DRG) shows tissue-specific changes in response to nerve injury (Gölzenleuchter et al., 2015). In Schwann cells, high-resolution methylation maps derived from reduced representation bisulfite sequencing showed global hypomethylation of promoter and enhancer regions of a large number of myelin-associated genes in neonatal mouse nerves during myelination relative to mature adult nerves. Hypomethylation of these genes was correlated with downregulation of gene expression, and artificially increasing global DNA methylation in Schwann cells in vitro and in vivo perturbed myelin gene expression and myelination (Varela-Rey et al., 2014). Additionally, a recent study performed methylated DNA immunoprecipitation sequencing on cultured Schwann cells that had been harvested from the distal stump of a 7-day cut adult rat sciatic nerve. Since their control condition, in this study, was Schwann cells from an uninjured brachial plexus, which they also cultured, it is difficult to interpret how their results are readily translatable to the changes that a Schwann cell undergoes during nerve injury in vivo (Zhou et al., 2017).

In our study, methylation maps generated from repair Schwann cells in injured nerves did not identify any significant global DM in any myelin-associated genes. In fact, using methods identical to those that we used previously to show differential methylation of tens of thousands of CpGs between rat strains or during development (Johnson et al., 2012, Johnson et al., 2014), out of over four million CpGs analyzed, we found only 853 DM CpGs. This would suggest that CpG methylation plays a less extensive role in adult cellular plasticity than it does in relation to germline DNA differences or in development. Additionally, this finding highlights that the downregulation of myelin genes that accompanies cessation of active myelination is regulated differently from that seen after injury. One possible caveat with this conclusion would be that our study under estimated the true number of DM CpGs. However, we maintained adequate average sequencing coverage of our samples, in line with current recommendations for WGSB-seq, and there was equal coverage of genomic regions of interest within individual samples (Figures S4B and S4G; Ziller et al., 2015). Furthermore, genome-wide methylation studies in other injury models, such as keloid scar formation and toxic injury to sperm, demonstrated a relatively low percentage of DM, comparable to our study (Jones et al., 2015, Dere et al., 2016).

Although a small percentage of DM CpGs were identified, the majority were located in gene regulatory areas of the genome, and a significant proportion were located within putative enhancer regions. This suggests that CpG methylation may have a biologically significant role after nerve injury. However, while a number of these potential enhancer regions were located near mapped active rat nerve injury enhancers (Hung et al., 2015), conservation of enhancer sequences is poor (Villar et al., 2015). Thus, mapping of active enhancers in mouse nerves after injury using H3K27acylation ChIP-seq will be required to show functional conservation and identify additional nerve enhancers in mouse. Enrichment analysis of DM CpGs revealed genes associated with ERBB, TGF-β, and neurotrophin signaling, which have all been previously identified in repair Schwann cells (Boerboom et al., 2017, Guertin et al., 2005, Scherer et al., 1993). This suggests that DNA methylation may have roles in modulating growth factor signaling in repair Schwann cells. When we looked in detail at one gene, *Nedd4l*, which is highly expressed in cultured Schwann cells, we found that there were two specific hypomethylated CpG clusters located within introns. One CpG cluster was located in a known mouse enhancer, which mapped very closely to an active enhancer in injured rat peripheral nerve (Hung et al., 2015). Both CpG clusters were hypomethylated in cultured mouse Schwann cells, but not in macrophages or nerve fibroblasts, suggesting that these changes are likely to be Schwann cell specific. NEDD4L is an E3 ubiquitin ligase that targets various ion channels and proteins involved in growth factor receptor signaling for proteasomal degradation, in particular components of TGF-β and nerve growth factor signaling (Goel et al., 2015). It is therefore possible that *Nedd4l* regulates signaling of multiple growth factors in repair Schwann cells, although further experiments will be needed to explore this.

While the role of CpG methylation in negatively regulating gene expression has been well documented in imprinting and X chromosome inactivation, the causal relationship between DNA methylation in other regions of the genome, including enhancer regions, with gene expression is less certain (Jones, 2012). In our study, enhancer DMRs were associated with both up- and downregulated genes. This is similar to findings for DMR in enhancer regions for Schwann cells during myelination (Varela-Rey et al., 2014). There is evidence from T cells, using luciferase reporter assays, that DMRs within lineage-specific enhancers can differentially regulate enhancer activity (Schmidl et al., 2009). Furthermore, genome-wide studies of the TF NRF in mouse embryonic stem cells identified that its binding to motifs in regulatory regions is influenced by local changes in CpG methylation (Domcke et al., 2015). Certainly, in our study, we found enrichment of DM CpGs within 40 bp of AP-1 TF-binding sites, in particular *Atf3* and *Fosl2* sites, suggesting that DNA methylation may have a biologically important and possibly causal relationship with TF binding, although the directionality of such a relationship has not been investigated here. Further analysis will be needed to test whether DM around TF-binding sites causally influences enhancer activity in repair Schwann cells.

A limitation of this study is that both RNA and methylation sequencing were carried out on whole nerve preparations. Nerves used for methylation analysis were desheathed beforehand, increasing Schwann cell purity to 80%. However, desheathing was not compatible with high-quality RNA extraction, with Schwann cell purity estimated between 48% and 74% in samples used for RNA-seq (Table S2C). To attempt to explore Schwann cell-specific methylation changes and RNA expression, we used purified cultures of Schwann cells, which replicate the expression of many repair Schwann cell genes (Arthur-Farraj et al., 2012). In addition, we repeated the analysis using nerve-derived fibroblasts (which would also include perineurial cells) and bone-marrow-derived macrophages treated with LPS, which constitute the two other main cell populations within the injured nerve. While we were able to show that 24 out of 33 RNAs were specifically highly expressed in Schwann cells in vitro, these findings still need to be confirmed in vivo using in situ hybridization. Furthermore, using Sanger sequencing, we confirmed similar methylation percentages of specific CpGs in cultured Schwann cells and whole nerve samples. Importantly macrophages and nerve fibroblasts showed different methylation profiles. While this finding lends weight to the likelihood that our whole-nerve sequencing results demonstrate DM within Schwann cells, single-cell analysis will reveal cell-type-specific and inter-cell heterogeneity of the DM profile.

In conclusion, this work provides a basis for understanding the molecular mechanisms underlying cell plasticity and a framework for functional studies aimed at identifying drug targets for the development of therapies for nerve injuries and peripheral neuropathies.

## Experimental Procedures

Animal experiments conformed to UK home office guidelines and were performed with institutional permission from University College London. 6- to 8-week-old male C57BL/6J mice were obtained from The Jackson Laboratory. *P0CRE*^+^/*c-Jun*^f/f^ male mice were generated as previously described (Parkinson et al., 2008). *c-Jun*^f/f^ littermates were used as controls.

### Nerve Transection and Cell Culture Experiments

All experiments used sciatic nerve cuts, which were performed at the sciatic notch as previously described (Arthur-Farraj et al., 2012). The proximal stump was diverted away from the distal stump, but not ligated. No animals showed any reconnection of axons between proximal and distal stumps. Nerves were harvested after 3 or 7 days, and the contralateral uninjured nerve was used as a control (referred to as uncut).

For cell culture methods, see Supplemental Experimental Procedures.

### RNA Extraction, Total RNA-Seq, and Small RNA-Seq Library Preparation

RNA was extracted from uncut and 3-day and 7-day cut sciatic nerves and cultures of neonatal mouse Schwann, adult nerve fibroblasts, and activated macrophages using TRIzol (Invitrogen). Nerves from two mice were pooled together for each biological replicate. The integrity and quantity of RNA was determined using Qubit (Invitrogen) and Agilent 2100 Bioanalyzers (Agilent Technologies).

1 μg total RNA was used to generate RNA-seq libraries using the TruSeq Stranded total RNA sample preparation kit (Illumina) according to the manufacturer’s instructions. An average of 61 million reads per sample was obtained from RNA-seq, with an average of 79.72% mapping to the GRCm38/mm10 genome. Hierarchal clustering of RNA-seq data revealed grouping of biological replicates between uncut and 7-day cut nerves (Figure S1).

Small RNA libraries were prepared using the TruSeq Small RNA sample preparation kit (Illumina) (Supplemental Experimental Procedures).

### Whole-Genome Shotgun Bisulfite Sequencing

DNA was extracted from desheathed uninjured and 7-day cut sciatic nerves and cultures of neonatal mouse Schwann, adult nerve fibroblasts, and activated macrophages using the QIAamp DNA micro kit (QIAGEN). The quantity of DNA was determined using a Qubit (Invitrogen). Libraries were prepared from individual nerves to generate methylation profiles from 100-bp paired-end reads on the HiSeq 2000 platform (Illumina). The full protocol was previously described (Johnson et al., 2012). Bisulfite-treated DNA was sequenced to a high depth with an average of 181 million reads per sample. The percentage of aligned pairs mapped to the genome with a mapping success of 72.9% (Figure S3A).

### Statistical Analysis

qPCR data are presented as arithmetic mean ± SEM, and statistical significance was demonstrated using an unpaired, two-tailed Student’s t test with Bonferroni correction for multiple testing where necessary. For sequencing data, detailed statistical methods are described in Supplemental Experimental Procedures. The Benjamini-Hochberg procedure was used for multiple testing.

## Author Contributions

Conceptualization, P.J.A.-F. and T.J.A.; Methodology, M.A., T.J.A., C.C.M., and P.J.A.-F.; Bioinformatics and Analysis, C.C.M. and A.B.; Investigation and Validation, P.J.A.-F., M.A., B.R., J.A.G.-S., and S.V.F.; Writing – Original Draft, P.J.A.-F. and C.C.M.; Writing – Review & Editing; P.J.A.-F., C.C.M., T.J.A., K.R.J., R.M., and M.A.; Funding Acquisition, T.J.A., K.R.J., and R.M.; Resources, T.J.A., K.R.J., and R.M.; Project Oversight, P.J.A.-F., T.J.A., and M.A.

## Data and Software Availability

The accession number for the sequence reads reported in this paper is ArrayExpress: E-MTAB-5633.

## Supplemental Information

Document S1. Supplemental Experimental Procedures, Figures S1–S5, and Tables S2 and S6

Table S1. List of Significantly Differentially Expressed RNAs and Annotated and Predicted lncRNAs 7 Days after Nerve Cut from the RNA-Seq Study, Related to Figures 1, 2, and 3 (A) All differentially expressed RNAs seven days after nerve cut from RNA-seq analysis. Differentially expressed annotated lncRNAs (B) and predicted lncRNAs (C). Average and condition specific base mean scores are shown along with the fold change, log2fold change, p-value and p-adjusted value. Nearest genes are displayed for lncRNAs.

Table S3. EMT-Associated Genes and miRNAs Expressed in the Injured Nerve, Related to Figures 1 and 4 List of enriched EMT genes between (A) uncut and seven day cut nerves from RNA-seq study, (B) miRNAs between uncut and three day cut nerves, (C) miRNAs between uncut and seven day cut nerves and (D) miRNAs between three and seven day cut nerves.

Table S4. List of Significantly Differentially Expressed miRNAs 3 and 7 Days after Nerve Cut from the Small RNA-Seq Study, Related to Figure 4 Differentially expressed miRNAs between (A) uncut versus three days after nerve cut, (B) uncut versus seven days after nerve cut and, (C), three days versus seven days after nerve cut. Average and condition specific base mean scores are shown along with the fold change, log2fold change, p value and p-adj value.

Table S5. DM CpGs and DMRs in 7-Day Cut Sciatic Nerve and DM CpGs that Are in Close Proximity to Mapped Active Enhancers and Their Associated Genes, Related to Figures 5 and 6 (A) Co-ordinates of each significantly DM CpG with supporting p-adjusted value and DM% between uncut and cut seven-day nerves. Individual C to CT % for each DM CpG and its biological replicates is provided. (B) Co-ordinates of DMR with total number of DM CpGs contained and its closest transcript/gene is shown. (C) DM CpGs that are in close proximity (< 400 bp) to active enhancers in rat injured sciatic nerve (Hung et al., 2015), mapped to the mm10 genome. Location of the sciatic nerve enhancer is given along with location of the DM CpGs in the mm10 genome, distance from enhancer to DM CpG and genes associated with each enhancer.

Document S2. Article plus Supplemental Information
