# Supplementary material for: Changes in the Coding and Non-coding Transcriptome and DNA Methylome that Define the Schwann Cell Repair Phenotype after Nerve Injury
Source: Cell Rep. 2017 Sep 12;20(11):2719–34. doi: 10.1016/j.celrep.2017.08.064 (PMC5608958; doi:10.1016/j.celrep.2017.08.064)
Supplement: Document S1. Supplemental Experimental Procedures, Figures S1–S5, and Tables S2 and S6 [file mmc1.pdf]

Cell Reports, Volume *20*

Supplemental Information

Changes in the Coding and Non-coding Transcriptome and DNA Methylome that Define the Schwann Cell Repair Phenotype after Nerve Injury

Peter J. Arthur-Farraj, Claire C. Morgan, Martyna Adamowicz, Jose A. Gomez-Sanchez, Shaline V. Fazal, Anthony Beucher, Bonnie Razzaghi, Rhona Mirsky, Kristjan R. Jessen, and Timothy J. Aitman

SUPPLEMENTAL INFORMATION

1. Supplemental Figures and Tables

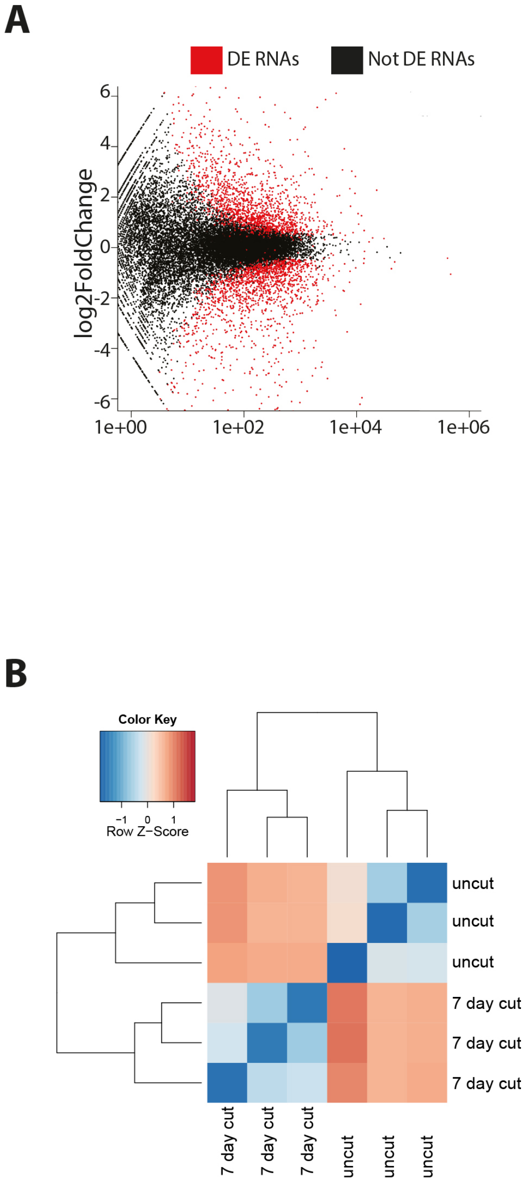

Supplemental Figure 1. RNA-Seq clustering of samples. Related to Figure 1, 2 and 3. (A) MA plot showing differentially expressed (red dots, DE genes) and non-differentially expressed (black dots, not DE genes) genes in uncut versus seven day cut nerves. (B) Clustering of RNA-Seq biological replicates for uncut and seven day cut nerves.

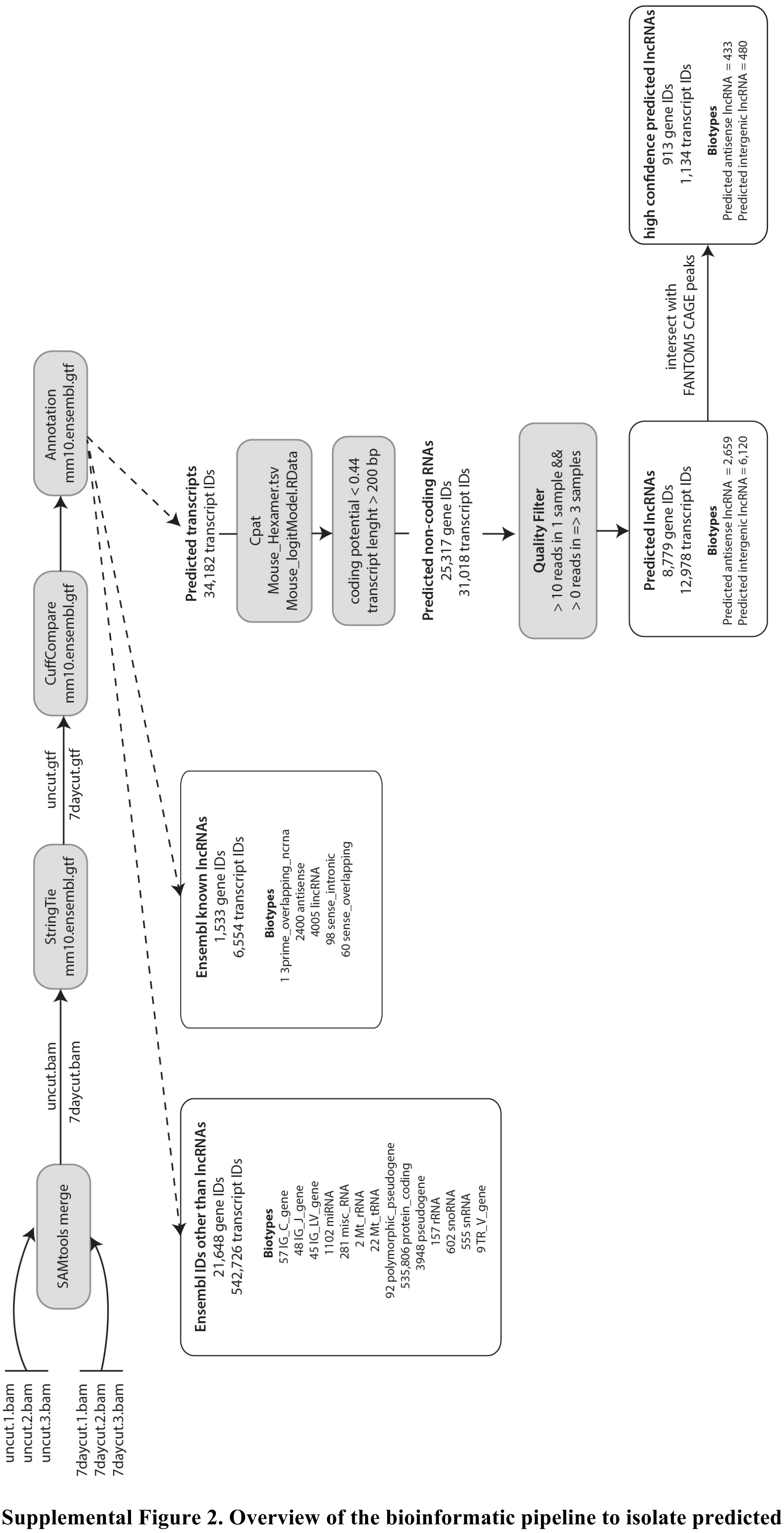

Supplemental Figure 2. Overview of the bioinformatic pipeline to isolate predicted high confidence Lnc RNA. Related to Figure 3. Predicted transcripts from RNA-Seq data, without an ENSEMBL ID, that had low protein coding potential and a minimum length of 200bp were intersected with FANTOM5 CAGE data to identify a set of high confidence lncRNAs (see Supplemental methods).

A

GENE	miRNAseq 3d cut FC	P value	qPCR 3d cut FC	P value
miR 17-5p	2.87	6.29E-06	1.60	0.016
miR 21a-3p	38.33	2.41E-43	18.98	3.50E-06
miR 34b-5p	5.28	3.73E-38	4.02	0.015
miR 34c-5p	6.51	7.50E-54	6.33	0.014
miR 132-3p	2.60	1.02E-07	3.47	0.0088
miR 142-3p	9.09	8.99E-18	6.78	0.00057
miR 362-5p	2.09	8.13E-07	1.94	0.0083
miR 92b-3p	-3.53	2.24E-09	-2.77	0.0085
miR 96-5p	-5.39	1.70E-47	-8.39	0.00027
miR 383-5p	-6.35	2.28E-78	-7.59	0.0017
miR 598-3p	-3.85	0.00013	-4.25	0.0069
miR 34a-5p	-1.04	0.96 NS	-2.31	0.016
miR 204-5p	-1.71	5.70E-23	-5.56	0.0011
miR 183-5p	-4.83	1.95E-46	-6.60	4.32E-05
miR 124-3p	-2.58	4.59E-15	-6.06	0.00017

B

GENE	miRNAseq 7d cut FC	P value	qPCR 7d cut FC	P value
miR 17-5p	1.91	0.0010	1.53	0.027
miR 21a-3p	26.75	2.41351E-43	10.97	3.90E-06
miR 34b-5p	18.15	1.90899E-23	10.10	0.0012
miR 34c-5p	9.86	7.37144E-26	19.14	0.014
miR 132-3p	2.60	1.01824E-07	1.56	0.024
miR 142-3p	4.89	8.99148E-18	5.52	0.0026
miR 362-5p	2.31	8.13282E-07	1.66	0.0072
miR 92b-3p	-5.01	2.23666E-09	-6.17	1.68E-05
miR 96-5p	-77.52	1.69617E-47	-144.02	2.54E-07
miR 383-5p	-155.79	2.27588E-78	-20.93	0.0010
miR 598-3p	-3.83	0.000127806	-4.86	0.0048
miR 34a-5p	-1.08	0.76 NS	-1.55	0.033
miR 204-5p	-4.97	5.70446E-23	-5.59	0.0012
miR 183-5p	-126.1	1.95E-46	-384.40	0.00012
miR 124-3p	-283.90	4.59193E-15	-33.56	9.67E-06

C

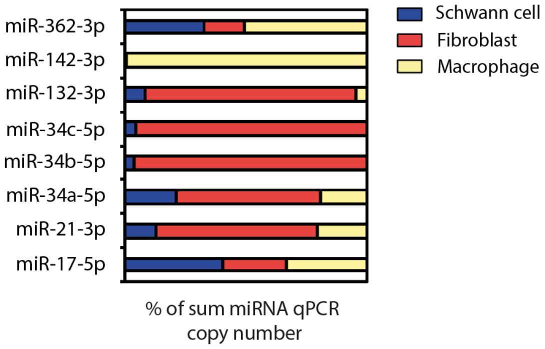

D

Enriched KEGG Pathway	miRNAs targeting enriched gene set (p-adj<0.01)
Focal adhesion	miR-29a, miR-29c, miR-409-3p, miR-485, miR-494, miR-770-5p
ECM-receptor interaction	miR-770
Leukocyte transendothelial migration	NS
**Enriched Gene ontology**	**miRNAs targeting enriched gene set (p-adj<0.001)**
regulation of cell morphogenesis	NA
fatty acid metabolic process	miR-15a, miR-15b, miR-32, miR-322, miR-378, miR-665, miR-758
regulation of signal transduction	miR-214
sterol metabolic process	miR-32
intracellular signaling cascade	miR-32, miR-199a-3p, miR-455, miR-873
regulation of small GTPase mediated signal transduction	NS
endocytosis	miR-32
**Enriched protein family**	**miRNAs targeting enriched gene set (p-adj<0.001)**
Pleckstrin homology	NS
Collagen triple helix repeat	miR-29c, miR-299, miR-29a, miR-29b, miR-455, miR-770-5p
Fibronectin type 3	NS
Plexin repeat	miR-431, miR-671-5p, miR-139-5p, miR-484, miR-874
Immunoglobulin	NS
Myosin_head	NS

E

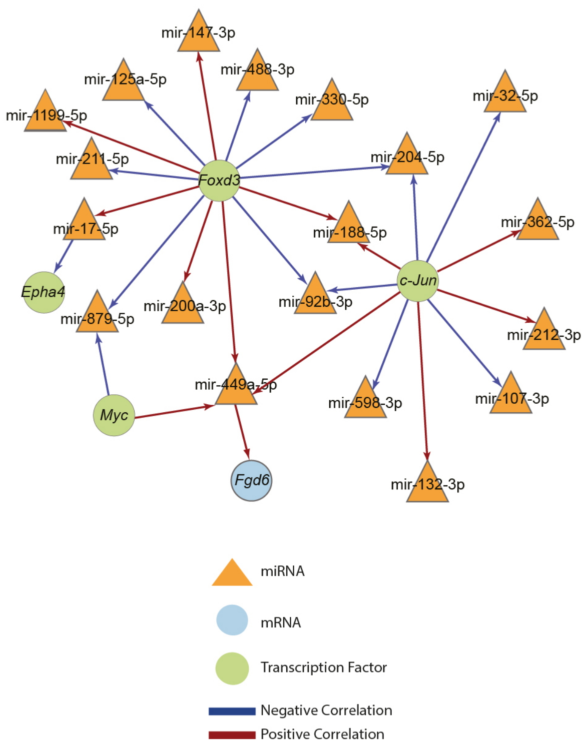

F

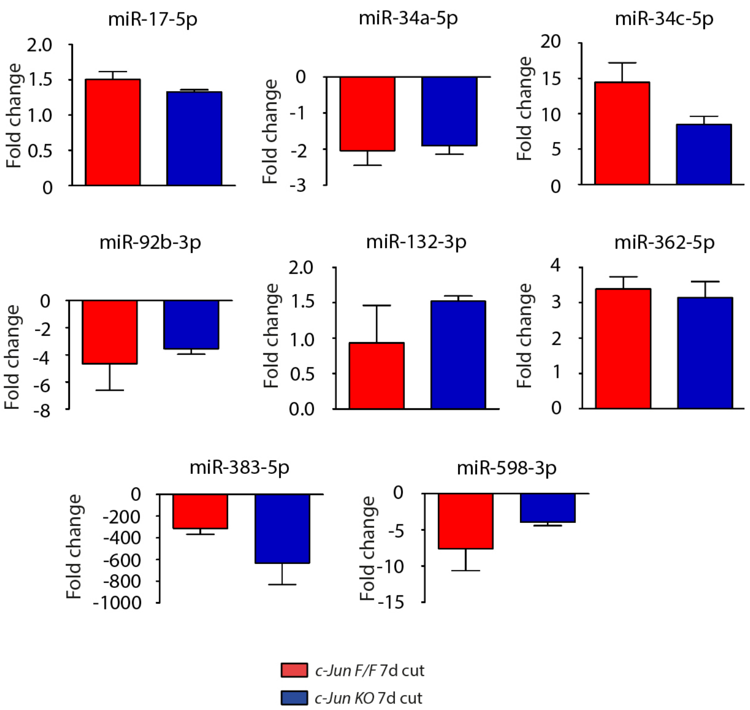

Supplemental Figure 3. miRNA profiles in the injured nerve. Related to Figure 4. (A and B) Fold change (FC) of miRNA expression values from RNA-Seq and qPCR in (A) three day cut and (B) seven day cut sciatic nerve samples compared to uncut nerves, along with associated p-adjusted values (n=5, p-adj<0.05). (C) Relative cell type expression of miRNAs in cultured mouse Schwann cells (blue), nerve fibroblasts (red) and activated macrophages (yellow) displayed as percentage of the sum of qPCR 2DCT (copy number) values from 1ug of RNA from each of the three cell types (n=3) (D) Enriched KEGG pathways, biological processes and protein families of miRNA targets, p-adj<0.05. (E) Magia[2] analysis showing putative regulatory network of miRNAs, transcription factors and genes targets following nerve cut. (F) Expression of miRNAs in *c-Jun* flox/flox (Control; red) and *c-Jun* null (P0Cre *c-Jun* flox/flox; blue) seven day cut nerves. Fold change is relative to the uncut control nerve (*c-Jun* flox/flox) (n=3, * p<0.05).

A

Mapping	aligned pairs	unique pairs	non-unique pairs
uncut 1	89608433 (92.8%)	74566713 (77.2%)	15041720 (15.6%)
uncut 2	88833543 (93.0%)	74643523 (78.2%)	14190020 (14.9%)
uncut 3	67984256 (92.4%)	56056794 (76.2%)	11927462 (16.2%)
Cut 7 days 1	90835398 (93.0%)	76880312 (78.7%)	13955086 (14.3%
Cut 7 days 2	84565914 (93.3%)	71835201 (79.3%)	12730713 (14.0%
Cut 7 days 3	78262623 (92.8%)	65711793 (77.9%)	12550830 (14.9%)
Average	83348361 (92.9%)	69949056 (72.9%)	13399305 (15.0%)

B

Coverage	Min	1st Qu.	Median	Mean	3. Qu	Max
uncut	5	6	8	10.44	11	65540
Cut 7 days	5	6	7	9.98	10	65540

C

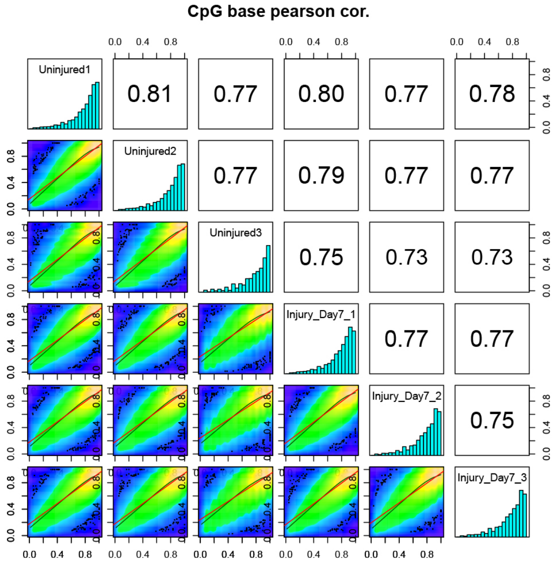

D

Annotation of individual DM CpGs	DM CpGs	promoter	exon	intron	3'UTR	intergenic	Enhancers	CpG Islands	CpG Shores	CpG Shelves
uncut v Cut 7days	853	41 (5%)	40 (5%)	285 (33%)	19 (2%)	468 (55%)	298 (34.9%)	3 (0.9%)	27 (3.2%)	37 (4.3%)

E

Annotation of 46 Clusters	DM CpGs in Clusters	promoter	exon	intron	3'UTR	intergenic	Enhancers	CpG Islands	CpG Shores	CpG Shelves
uncut v Cut 7days	114	8 (7.0%)	6 (5.3%)	60 (52.6%)	1 (0.9%)	39 (34.2%)	57 (50%)	0	0	10 (8.8%)

F

Enrichment of clustered DM CpGs	single CpGs	CpGs in Clusters	p-value	OR	95% CI	Enriched
promoter	41	8	2.39E-01	1.61	0.63, 3.69	No
exon	40	6	8.11E-01	1.15	0.39, 2.86	No
intron	285	60	5.95E-06	2.54	1.67, 3.86	Yes
3' UTR	19	1	4.96E-01	0.35	0.01, 2.29	No
intergenic	468	39	2.55E-06	0.38	0.24, 0.58	No
Enhancers	298	57	4.55E-04	2.06	1.36, 3.14	Yes

G

Uncut_1	min	1st Qu.	Median	3rd. Qu	Max
Exon	1	7	12	17	2744
Promoter	1	6	11	16	1252
3-UTR	1	6	10	15	2744
Enhancer	1	6	10	15	2000600
Intron	1	5	10	15	30913
Other	1	5	9	15	313924
**Uncut_2**	**min**	**1st Qu.**	**Median**	**3rd. Qu**	**Max**
Exon	1	7	11	16	2495
Promoter	1	6	10	15	1054
3-UTR	1	5	9	14	2495
Enhancer	1	6	10	14	1794100
Intron	1	5	9	14	28334
Other	1	4	8	14	255723
**Uncut_3**	**min**	**1st Qu.**	**Median**	**3rd. Qu**	**Max**
Exon	1	7	12	16	2597
Promoter	1	6	11	16	1090
3-UTR	1	6	10	15	2597
Enhancer	1	6	10	15	1770040
Intron	1	5	9	14	28274
Other	1	5	9	14	230895

7daycut_1	min	1st Qu.	Median	3rd. Qu	Max
Exon	1	6	9	13	1744
Promoter	1	5	8	12	822
3-UTR	1	5	8	12	1744
Enhancer	1	5	8	12	1421520
Intron	1	5	8	11	36553
Other	1	4	7	11	290759
**7daycut_2**	**min**	**1st Qu.**	**Median**	**3rd. Qu**	**Max**
Exon	1	5	9	13	1691
Promoter	1	5	8	12	1011
3-UTR	1	4	8	11	1691
Enhancer	1	4	8	12	1702930
Intron	1	4	7	12	22916
Other	1	4	7	12	318952
**7daycut_3**	**min**	**1st Qu.**	**Median**	**3rd. Qu**	**Max**
Exon	1	6	9	13	1973
Promoter	1	5	8	12	793
3-UTR	1	5	8	12	1973
Enhancer	1	5	8	12	1574250
Intron	1	4	8	12	32285
Other	1	4	7	11	307265

Supplemental Figure 4. Overview of Whole genome shotgun bisulfite data quality and results. Related to Figure 5 and 6. (A) Mapping results for aligned unique and non-unique pairs across uncut and seven day cut replicates. (B) Average depth of coverage across CpGs for uncut and seven day cut samples. (C) Pearson correlation of CpG methylation states between uncut and cut samples and technical replicates. (D and E) The genomic annotation of (D) individual DM CpGs and (E) clustered CpGs is provided. (F) Fishers exact test of clustered DM CpGs versus single DM CpGs occurrence in various genomic loci. Odd's ratio (OR) and 95% confidence interval (CI) are displayed. (G) Read coverage across different genomic regions for each individual sample sequenced.

A

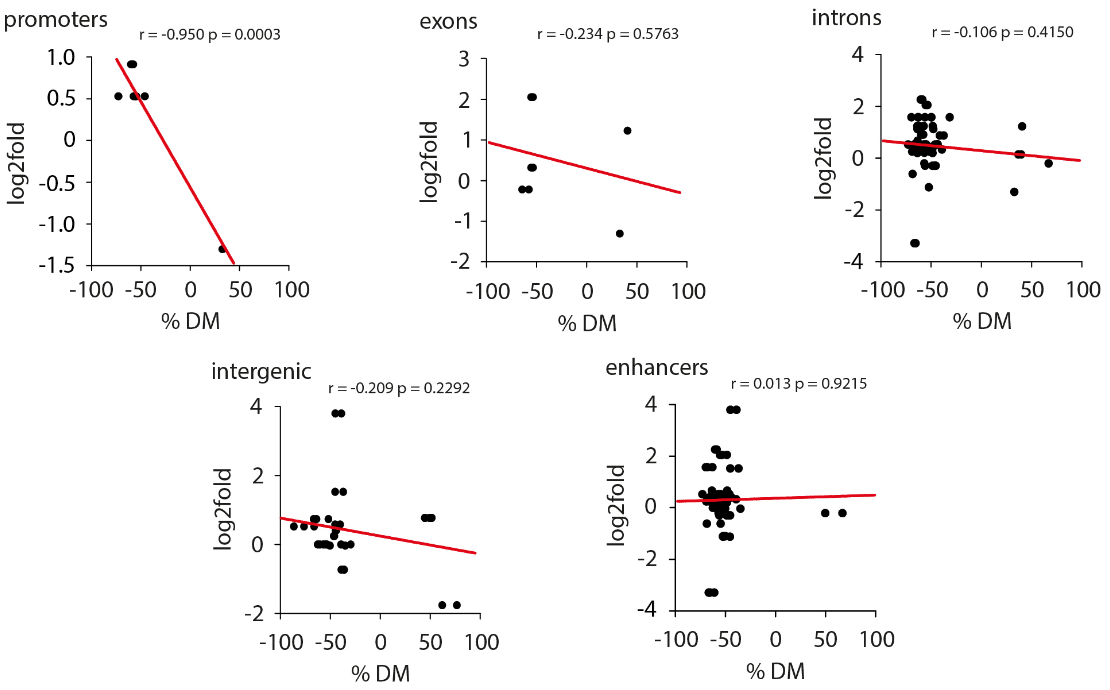

B

Cluster Location	# DM CpGs	Δ %	Closest Gene	Annotation	FC	p-adj	r
chr8:125498650-125498713	3	-37.1	*Sipa1l2*	6055 up	-1.66	2.01E-04	+ve
chr11:51881210-51881377	3	-60.8	*Jade2*	24085 up	1.67	4.11E-02	-ve
chr12:100878642-100878656	3	-52.5	*9030617O03Rik*	Exon	1.25	1.00E+00	NA
chr18:65207791-65207813	3	-66.9	*Nedd4l*	Intron	2.99	1.49E-11	-ve
chr1:88587585-88587674	3	-59.9	*Glrp1*	77700 up	Inf	1.00E+00	NA
chr4:35096498-35096508	3	-64.4	*Mob3b*	Intron	-9.73	6.29E-31	+ve
chr6:31080847-31080985	3	-76.2	*Gm13834*	6927 down	1.44	6.83E-01	NA
chr17:84333040-84333192	4	-49.1	*Thada*	Intron	-1.22	8.33E-01	NA
chr8:14891377-14891676	4	-57.8	*Cln8*	Intron	2.36	1.79E-05	-ve
chr16:16988992-16989128	4	-58.3	*Mapk1*	Intron	1.39	7.84E-01	NA
chr14:105870531-105870582	5	48.2	*Spry2*	22221 down	1.71	2.46E-02	+ve
chr18:65040023-65040169	5	-49.9	*Nedd4l*	Intron	2.99	1.49E-11	-ve
chr9:63743959-63744150	5	-57.3	*Smad3*	Promoter	1.44	4.04E-01	-ve

Supplemental Figure 5. Correlation of DM CpGs with expression of nearest gene. Related to Figure 6. (A) Correlation (r) of percentage DM of each DMR located within promoters, exons, introns, intergenic and enhancer regions with the log2fold expression of the nearest gene from RNA-Seq data. (B) Location and number of differentially methylated CpGs (# DM CpGs) in each cluster (DMR), along with percentage change in methylation (Δ %), closest gene to DMR and location of DMR within that gene (annotation) fold change from RNA-Seq analysis (FC), p-adj<0.05, and correlation with differential methylation are displayed (r).

A

AP-1 transcription factors

GENE	RNAseq 7d cut FC	P adj value	qPCR 7d cut FC	P adj value
ATF3	19.46	1.41E-077	13.76	0.0037
c-Jun	6.63	1.96E-038	5.097	6.41E-06
Fosl2	5.90	1.26E-33	5.57	5.21E-07
Fos	3.99	1.17E-08	5.43	0.013
Fosl1	3.34	0.15 NS	-1.65	0.085 NS
JunB	2.62	8.94E-05	-4.48	0.029 NS
JunD	-1.16	0.068 NS	1.19	0.53 NS
ATF2	1.00	0.88 NS	-1.02	0.76 NS
FosB	-1.49	0.27 NS	-1.62	0.13 NS

LncRNAs

GENE	RNAseq 7d cut FC	P adj value	qPCR 7d cut FC	P adj value
H19	7.84	1.65E-060	6.4	0.004
Meg3	2.31	1.77E-013	NA	NA
Meg3 transcript 1	N/A	N/A	2.66	0.028
Meg3 transcript 2	N/A	N/A	2.84	2.29E-03
Meg3 transcript 3	N/A	N/A	1.70	0.10 NS
Rian	1.97	4.97E-008	1.95	0.012
Sox2ot	1.78	0.00071	2.64	0.03

Myelin genes

GENE	RNAseq 7d cut FC	P adj value	qPCR 7d cut FC	P adj value
Egr2	-8.6	6.53E-31	-8.00	0.0013
Mpz	-74.65	1.47E-189	-403.33	0.0054

Putative Repair program genes

GENE	RNAseq 7d cut FC	P adj value	qPCR 7d cut FC	P adj value
Rgs8	334.80	1.42E-063	29.05	0.00016
Slc17a8	199.68	2.28E-064	234.51	0.039
Runx2	99.39	1.12E-107	80.70	0.0045
Gpnmb	58.35	9.76E-141	36.58	0.0072
Gadd45a	50.25	1.38E-078	39.95	0.0037
Gria1	41.29	7.16E-066	5.87	0.012
Fgd3	40.88	1.2064E-99	106.21	0.013
Rgs16	14.72	1.16E-040	29.36	0.013
Nr1h4	13.96	4.08E-009	38.00	0.017
Arl4c	12.57	4.77E-027	9.20	0.014
Sox4	9.29	2.56095E-38	8.06	0.0026
Mmp17	7.83	9.33E-034	7.88	0.0011
Adam11	7.21	6.43E-01	4.18	8.0049E-05
C1qb	6.53	6.52E-034	8.39	0.015
Nav2	6.43	6.59E-046	29.01	7.011E-06
Foxd3	4.02	1.73E-012	3.62	0.0003
Socs3	3.18	2.27E-008	6.21	0.00039
Nedd4l	2.99	4.12E-013	4.75	0.037
Cln8	2.36	1.06E-006	4.50	0.025
Mgp	2.26	2.72E-008	10.77	0.0057
Dnmt1	1.74	0.00014	3.91	0.046
Sox2	7.78	9.57E-018	7.47	0.0017

B

Gene	SC % of sum copy no.	Fibroblast % of sum copy no.	Macrophage % of sum copy no.	SC vs. Fibro p adj	SC vs. Macro p adj
Adam11	97.58	2.42	0.00	8.64E-05	6.88E-05
Arl4c	33.37	38.27	28.36	0.719 NS	0.273 NS
Atf2	59.14	23.07	17.79	1.79E-04	2.80E-04
Atf3	71.48	0.55	27.98	0.011	0.028
C1qb	0.30	0.33	99.36	0.843 NS	0.025
Cln8	76.53	5.93	17.55	2.10E-03	3.10E-04
Fgd3	98.14	0.94	0.91	7.15E-03	7.16E-03
Fos	67.07	4.89	28.04	0.019	3.35E-03
FosB	56.16	28.44	15.40	0.111 NS	0.087 NS
Fosl1	85.48	14.06	0.46	8.55E-04	5.32E-03
Fosl2	77.32	15.61	7.07	0.025	0.013
Foxd3	98.77	1.23	0.00	0.027	0.026
Gadd45a	95.07	4.26	0.67	0.040	0.037
Gpnmb	8.47	0.19	91.34	0.293 NS	0.007
Gria1	99.10	0.70	0.20	0.009	0.009
H19	79.01	20.89	0.10	1.67E-03	0.039
c-Jun	88.51	4.31	7.18	0.027	0.025
JunB	10.70	10.82	78.48	0.929 NS	0.023
JunD	58.04	28.82	13.14	0.023	7.24E-03
Meg3t1	45.61	53.59	0.80	0.736 NS	0.045
Meg3t2	45.82	54.14	0.05	0.562 NS	0.030
Meg3t3	41.88	57.65	0.47	0.107 NS	0.004
Mmp17	97.69	2.31	0.01	0.003	0.003
Mob3b	5.79	1.52	92.68	0.099 NS	0.056 NS
Nav2	92.93	3.50	3.57	0.020	0.020
Nedd4l	82.89	4.15	12.96	2.20E-03	1.16E-03
Nrlh4	81.34	17.99	0.67	0.070	0.054
Rian	36.68	63.22	0.10	0.027	0.024
Rgs8	82.17	17.53	0.30	1.97E-04	0.017
Rgs16	75.52	23.62	0.86	6.78E-04	0.011
Runx2	92.99	6.23	0.78	4.38E-06	4.38E-06
Slc17a8	86.47	1.26	12.27	5.36E-03	7.95E-03
Socs3	2.77	2.92	94.30	0.752 NS	0.016
Sox2	99.14	0.77	0.09	0.013	0.013
Sox2ot	98.64	1.19	0.17	0.044	0.044

C

i

TOTAL CELLS (n=3)	SCHWANN CELLS	MACROPHAGES	FIBROBLASTS	OTHER
311	229	41	35	6
	74%	13%	11%	2%

ii

UNTREATED (n=4)	IMMUNOPANNED (n=4)	DESHEATHED (n=4)	IMMUNOPANNED + DESHEATHED (n=4)
48%	62%	80%	89%

Supplemental Table 2. Confirmation of RNA-Seq coding and non-coding RNAs by qPCR in whole nerve and primary cultured cell types and estimates of Schwann cell purity in injured nerves. Related to Figure 1, 2 and 3 and experimental procedures. (A) Fold change for RNA-Seq and qPCR gene expression values (ΔCT values) in seven day cut compared to uncut nerves for AP-1 TF, lncRNAs, Myelin genes and putative repair programme genes. P-adjusted values are given. (B) Relative cell type expression for repair program coding RNAs and lncRNAs in cultured mouse Schwann cells, nerve fibroblasts and activated macrophages displayed as percentage of the sum of qPCR copy number values from 1ug of RNA from each of the three cell types (n=3). Separate p-adjusted values given for statistical comparisons of Schwann cell values to fibroblasts and to macrophages, respectively. RNAs labeled in blue are statistically more highly expressed in Schwann cells than in fibroblasts or macrophages. (C) (i) Cells counts from Electron micrographs at 5000x magnification of seven day cut distal stumps (5mm from cut site; n=3). (ii) Percentage purity of Schwann cells from enzyme dissociated cultures of adult mouse seven day cut nerve, immunopanned, desheathed, immunopanned and desheathed or left untreated (n=4).

A

GENE	PRIMER NAME	PRIMER SEQUENCE
Adam11	**Adam11_For2**	**GGAGAGGTCCGAAAGCCACA**
	Adam11_Rev2	CCGGGATGACGAAACTCACC
Ankrd27	**Ank27_For2**	**TCCTGCCAGTTCGAGTCCTAT**
	Ank27_Rev2	AATGACGACAGCCTTTCCATC
Arl4c	**Arl4c_For1**	**CTGCACATCGTTATGCTGGG**
	Arl4c_Rev1	GGTGTTGAAGCCGATAGTGGG
ATF2	**ATF2_For4**	**ACTCCAACGCCAACAAGATTC**
	ATF2_Rev4	TCTACAACAGAAGGCTCCTCAAT
ATF3	**ATF3_For1**	**CCTTGATAGGTGGGGTGGGA**
	ATF3_Rev1	AACACCAGGCTCTGAAACGG
C1qb	**C1qb_For1**	**GGCTCTGGGAATCCACTGCT**
	C1qb_Rev1	GCCCAGGACACATGGAGAAAA
Canx	**Canx_For2**	**CTTCCAGGGGATAAAGGACTTGT**
	Canx_Rev2	ACATAGGCACCACCACATTCTA
Cln8	**Cln8_For2**	**CAGCTCTCATCGTCCCTGAAT**
	Cln8_Rev2	GGTGATGTGAAACCAACACCA
Dnmt1	**Dnmt1_Fw1**	**CTAGTTCCGTGGCTACGAGG**
	Dnmt1_Rev1	AGTTCCCCTCTTCCGACTCT
Egr2	**Egr2_For2**	**CCGTATCCGAGTAGCTTCGC**
	Egr2_Rev2	TCAATGGAGAATTTGCCCATGT
Fgd3	**Fgd3_For2**	**CACTCAGGACCGACACCTCT**
	Fgd3_Rev2	CAGGCTGCGTTTGGTTTCAC
Fos	**cFos_For3**	**GGTTTCAACGCCGACTACGA**
	cFos_Rev3	GTTGGCACTAGAGACGGACAG
FosB	**FosB_For2**	**ACTGGAGAGAAAGTTTTGTGGGT**
	FosB_Rev2	AGCGTCCCAAGAAATGAGGG
Fosl1	**Fosl1_For1**	**GCATGTACCGAGACTACGGGGAA**
	Fosl1_Rev1	TGCACCATCCAGTGCAGTTCCT
Fosl2	**Fosl2_For3**	**CACGCTCACATCCCTACAGTC**
	Fosl2_Rev3	CCGGATTCGACGCTTCTCCT
Foxd3	**Foxd3_Fw1**	**GACCCCGAACAAGCCCAAG**
	Foxd3_Rev1	GAAAACGGTTGCTGATGAACTC
Gadd45a	**Gadd45a_Fw1**	**CTGCAGAGCAGAAGACCGAA**
	Gadd45a_Rev1	GGGTCTACGTTGAGCAGCTT
GAPDH	**GAPDH_Fw3**	**GGGTGTGAACCAGAGAAA**
	GAPDH_Rev3	GTCTTCTGGGTGGCAGTGAT
GPNMB	**GPNMB_For1**	**ATTACGTGGCTGGTCTTCGG**
	GPNMB_Rev1	CCTTCTGGCATCTGGGGAAC
GRIA1	**GRIA1_For1**	**AAGGGGAATGTGGAAGCAAGG**
	GRIA1_Rev1	CGACTCGCTACGGGATTTGT
H19	**H19_For1**	**GCTCTGGCAAAGTCCCAAGT**
	H19_Rev1	AGTGCCTCATGGGAATGGTG
c-Jun	**cJun_For1**	**CCTTCTACGACGATGCCCTC**
	cJun_Rev1	GGTTCAAGGTCATGCTCTGTTT
JunB	**JunB_Fw4**	**TCACGACGACTCTTACGCAG**
	JunB_Rev4	CCTTGAGACCCCGATAGGGA

GENE	PRIMER NAME	PRIMER SEQUENCE
JunD	**JunD_For2**	**CACGCAAGAACGCATCAAGG**
	JunD_Rev2	GTTGACGTGGCTGAGGACTT
Meg3t1	**Meg3t1_For1**	**GTCCACGTTTTCGTTGCTCC**
	Meg3t1_Rev1	ATGCTGGGAAAGCTGGTTGT
Meg3t2	**Meg3t2_For2**	**GTCCACGTTTTCGTTGCTCC**
	Meg3t2_Rev2	ATGCTGGGAAAGCTGGTTGT
Meg3t3	**Meg3t3_For2**	**ACATCATCGGCTCACACCAG**
	Meg3t3_Rev2	CAGGCAACTTGAGGGAGTGG
MMP17	**MMP17_For3**	**AGTGTGGACGCCGTGTATG**
	MMP17_Rev3	GGAGGCTGAAGTCGGAGACA
MPZ	**MPZ_For1**	**CGGACAGGGAAATCTATGGTGC**
	MPZ_Rev1	TGGTAGCGCCAGGTAAAAGAG
Nav2	**Nav2_For2**	**AGCCTGCTCTGGAGGAACTCA**
	Nav2_Rev2	CTCAGTGGTGACGTTGGTGTC
Nedd4l	**Nedd4l_For1**	**GTCCGGCTGTTCCGTACTC**
	Nedd4l_Rev1	AGGCCATAGTAGGGGTTAAACAT
Nr1h4	**Nr1h4_For**	**GGCAGAATCTGGATTTGGAATCG**
	Nr1h4_Rev	GCCCAGGTTGGAATAGTAAGACG
Rgs16	**Rgs16For1**	**TCCAGGGCTCACCACATCTT**
	Rgs16Rev1	TCTCCATCAATGTGCGGGTC
Rgs8	**Rgs8_For4**	**GCAGGAACAAAGGCATGAGGA**
	Rgs8_Rev4	TGCTTCTTCCGTGGAGAGTCT
Rian	**Rian_For2**	**GACTCATCCTGACGTGCCAA**
	Rian_Rev2	TTCCCTTACCCACACAGGGG
Runx2	**Runx2_For1**	**CACCTCTGACTTCTGCCTCTG**
	Runx2_Rev1	CTGGGTAGTGCATTCGTGGG
Slc17a8	Slc_Rev1	AGCTGACAAGAGACCCACCT
	**Slc_For2**	**CCTATGCTGGGGCAGTTGTT**
Socs3	**Socs3_Fw1**	**GCGGGCACCTTTCTTATCC**
	Socs3_Rev1	CTGGAGGCGGCATGTAGTG
Sox2	**Sox2_For1**	**TCGCAGGGAGTTCGCAAAAG**
	Sox2_Rev1	ACCCAGCAAGAACCCTTTCC
Sox2ot	**Sox2ot_For2**	**GAACGGGCACACATCAAGCA**
	Sox2ot_Rev2	GCCCCCTATACAACACTGCC
Sox4	**Sox4_For2**	**CCAGTTCTTGCACGCTGTTTC**
	Sox4_Rev2	ATGTATGTTTCTCCCTCCCTCCT

B

Primer Name	Product No.	Target Sequence 5'-3'
hsa-miR-17-5p	204771	CAAAGUGCUUACAGUGCAGGUAG
mmu-miR-21a-3p	205400	CAACAGCAGUCGAUGGGCUGUC
mmu-miR-34a-5p	204486	UGGCAGUGUCUUAGCUGGUUGU
mmu-miR-34b-5p	205075	AGGCAGUGUAAUUAGCUGAUUGU
mmu-miR-34c-5p	202017	AGGCAGUGUAGUUAGCUGAUUGC
hsa-miR-92b-3p	204383	UAUUGCACUCGUCCCGGCCUCC
hsa-miR-96-5p	204417	UUUGGCACUAGCACAUUUUUGCU
hsa-miR-124-3p	204319	UAAGGCACGCGGUGAAUGCC
hsa-miR-132-3p	204129	UAACAGUCUACAGCCAUGGUCG
hsa-miR-142-3p	204291	UGUAGUGUUUCCUACUUUAUGGA
hsa-miR-183-3p	204652	UAUGGCACUGGUAGAAUUCACU
hsa-miR-204-5p	205708	UUCCCUUUGUCAUCCUAUGCCU
hsa-miR-362-5p	205073	AAUCCUUGGAACCUAGGUGUGAAU
hsa-miR-383-5p	205000	AGAUCAGAAGGUGACUGUGGCU
hsa-miR-598-3p	205045	UACGUCAUCGUCGUCAUCGUUA

C

Chr	Str	Start	End	Gene	Pr	Sequence
1	+ve	8858<br>7086	8858<br>8175	*Arl4c*	F	**TTTTTTGAAAATGGAGGAAATTATAGA**
					R	AAAATAAACCCAAATCCTCACACATAC
4	-ve	3509<br>5999	3509<br>7009	*Mob3b*	F	**ATGAGTTAGGTGTGATTGGGTTAT**
					R	AACCCAACTAAAAACTCAATATAAAATT
8	+ve	1489<br>0878	1489<br>2177	*Cln8*	F	**TTAGGGGATAGGGTGTGTTAGTTTAGAG**
					R	ACAACAAAATCCACTTCCTCAACC
10	-ve	8945<br>2630	8945<br>3668	*Nr1h4*	F	**GAGATAAAGGAGGGTGGAGATTAAATAAAT**
					R	AAAAACCCCATTACAACCAAAAAC
18	-ve	6503<br>9524	6504<br>0670	*Nedd4l*	F	**GAGAGTTTTTTTAAGATTATTGGGATTA**
					R	ACCTTCCTTTAAACTACCTTCAAATACT

Supplemental Table 6. List of primers for quantitative PCR and bisulfite PCR. Related to Figure 2 to 6 and experimental procedures. (A) Primers for qPCR (B) miRNA qPCR and (C) Primers for bisulfite PCR.

2. Supplemental Experimental Procedures

Cell culture preparation

Neonatal mouse Schwann cell culture and cell expansion was performed as previously described. Cultures were consistently >95% pure judged by 04 and S100b (Arthur-Farraj et al., 2011; Stevens et al., 1998). Adult seven day cut nerves were desheathed and dissociated with enzyme cocktail (Woodhoo et al., 2004) for 24 hours before plating on PLL/laminin coverslips plates in Defined medium (Arthur-Farraj et al., 2011) with 0.5% horse serum (Invitrogen, UK). Adult peripheral nerve fibroblasts were obtained from seven day cut nerves, which were not desheathed and were then dissociated in enzyme cocktail for 24 hours before being plated directly onto uncoated tissue culture dishes in DMEM 10% foetal calf serum (Perbio, UK) and expanded for 14 days, re-plating three times to remove Schwann cells. Cultures were >99% pure judged by S100b negative immuno-staining. Bone marrow derived macrophage cultures were prepared as described previously (Behmoares et al., 2010).

Assessment of Schwann cell purity in nerve samples for sequencing

The uncut nerve consists of predominantly of Schwann cells (>90%). Seven days after nerve cut, Schwann cells still make up greater than 70% of the total cell number in the nerve, the majority of the rest being fibroblasts, macrophages and perineural cells. For all DNA sequencing experiments, adult nerves were desheathed, enabling greater than 80% cell purity in cut nerve samples, when tested in cell dissociation experiments. For RNA sequencing experiments, adult nerves were not desheathed as this compromised RNA integrity (Table S2C).

Quantitative RT-PCR

cDNA was synthesized, using 1μg of total RNA per sample and iScript cDNA synthesis kit (BioRad) following manufacturers instruction. MiRCURY LNA Universal RT microRNA PCR kits (Exiqon) were used for microRNA qPCR. For standard qPCR, *Ankrd27* and *Canx* were used as housekeeping genes. All primers were designed using Primer blast (NCBI)(Ye et al., 2012). Primer sequences are detailed in Table S6A. Micro RNA Primers were commercially deigned (Exiqon). miR191-5p was used as a housekeeping miRNA. For target miRNA sequences see Table S6B Standard curves and fluorescent quantitation PCR were performed on 7900HT Fast Real Time PCR Machine (Applied Biosystems) and cDNA quantified according to the manufacturer's software recommendation.

Quantification of relative RNA expression in specific cultured cell types

In order to compare the relative level of expression of RNAs between different cell types *in vitro*, we performed qPCR, using cDNA made from 1ug of RNA extracted from each of the following cell types: cultured mouse Schwann cells; cultured mouse nerve-derived fibroblasts; and cultured mouse bone marrow-derived macrophages treated with Lipopolysaccharide (LPS). The mean qPCR copy number values (n=3) for each cell type were then added together to achieve a sum total. The mean copy number value for each cell type was then expressed as a percentage of the sum total. Preferential expression of an RNA in a particular cell type was determined by an unpaired two-tailed students t-test with Bonferonni correction for multiple testing (p-adjusted <0.05).

Stranded RNA-seq

Briefly, cytoplasmic and mitochondrial rRNA was removed using biotinylated, target specific oligos combined with Ribo-Zero rRNA removal beads. Following purification, RNA was fragmented using divalent cations under elevated temperature and cleaved fragments were copied onto first strand cDNA using reverse transcriptase and random primers. Next, the second strand was synthesised using DNA Polymerase I and RNase H. cDNA 3' ends were adenylated and indexed adapters ligated followed by 10 cycles of library amplification. Finally, the libraries were purified using AMPue XP Beads (Beckman Coulter) and quality was evaluated using Agilent 2100 Bioanalyzer. Three libraries were run on a single lane of the HiSeq 2000 platform (Illumina) to generate 100bp paired-end reads.

Small RNA Seq

Briefly, 3` and 5` indexed adapters were ligated to 1mg of total RNA and the product of this reaction was RT-PCR amplified. Small RNA library was size selected using Pippin Prep elution system (Sage Science). The quality of the libraries was evaluated using Agilent 2100 Bioanalyzer. All the libraries were pooled together and run on two lanes of the HiSeq 2000 platform to generate 50bp single-end reads.

Bisulfite PCR
Primers for bisulfite PCR were designed using MethPrimer (Li and Dahiya, 2002) and sequences are listed in Table S6C. Bisulfite PCR was performed using PfuTurbo Cx hotstart DNA polymerase (Agilent) according to manufacturers guidelines. PCR conditions used 95°C for 2 min; 95°C for 30s, 52°C-62°C for 30s and 72°C for 1min for 40 cycles; then 72°C for 10min.

Analysis of CpG dinucleotide methylation using Bisulfite-Sanger sequencing
Genomic DNA was bisulfite treated using MethylCode ™ bisulfite conversion kit (Thermofisher). Bisulfite PCR was performed and PCR products were then purified and sent for Sanger sequencing. Sequences were analysed using Sequencher® version 5.4.1 sequence analysis software (Gene Codes).

Identification of miRNA sequences present in samples
Illumina adapters were clipped from the 3' end and sequences shorter than 18 nucleotides were discarded. MirDeep2 (v.2.0.0.5) (Friedlander et al., 2012) was used to determine the presence and quantity of miRNAs based on mouse precursor sequences and mature sequences from mouse and rat with miRBase release 19 (Kozomara and Griffiths-Jones, 2011).

Identification of transcriptionally active sequences in samples
RNA-Seq reads were aligned with Tophat splice junction mapper (v2.0.8) (Trapnell et al., 2009) and short read mapper Bowtie (v2.1.0) (Langmead and Salzberg, 2012) using the stranded parameter (fr-firststrand) against the UCSC Mouse genome reference sequence assembly (GCRm38/mm10) and the Ensembl transcript annotations (version GRCm38.73). Gene based read counts were obtained using HTSeq count module (v0.5.4p3) (Anders et al., 2015).

Identification of Enhancer and their association to genes
Putative enhancers were defined by genomic locations derived from Encode and the UCSC browser (ENCODE PMID:22763441 and UCSC PMID: 25428374). Using GREAT (http://bejerano.stanford.edu/great/public/html/index.php) the DM CpGs overlapping enhancers that were associated with a gene were identified using basal plus extension default parameters, which search 5kb upstream of a TSS, 1kb downstream plus distal extension up to 1 Mb from the TSS.

Mapping Rat enhancers to genomic loci in mouse
Rat sciatic nerve H3K27ac Chip peaks were obtained from NCBI GEO under accession number GSE64103. Of the 31,894 active enhancer regions identified in the rn5 genome, 2,529 of these loci map to the mm10 genome using UCSC liftOver in conjunction with the rn5ToMm10.over.chain file.

Assessing DM CpGs within topological associated domains of myelin-associated genes.
Topological associated domains for the mm9 genome were obtained from the Mouse Encode project (http://chromosome.sdsc.edu/mouse/hi-c/download.html) (Pope et al., 2014). The mm9 co-ordinates were converted to mm10 using liftOver. The TAD domains were between 0.8 and 1.9 Mb in size. The total number of DM CpGs and the average DM CpG % were calculated within the TAD that contained the following myelin-associated genes studied by (Varela-Rey et al., 2014); *Nab1*, *Cyp51*, *Slc6a13*, *Prx*, *Lgi4*, *Slc27a1*, *Egr2*, *Kng1*, *Cd9*, *Cebpa*, *Pmp22*, *Nfatc1* and *Cnp*.

De novo motif searching around DM CpG loci
We performed a de novo motif search using findMotifsGenome.pl which is part of the Homer package. We searched for enrichment of motifs of 6, 8, 10, 12, 14 and 16 bp in length within a 40bp window centered on the 853 DM CpGs identified in the mm10 genome.

Differential Expression analysis of RNA-Seq and small RNA-Seq data Differential expression (DE) analysis was performed using DESeq Bioconductor package (Anders and Huber, 2010). Independent filtering was applied to remove the genes in the lowest 40% quantile before statistical testing was employed. The empirical dispersion values for each gene estimated using a conservative approach (sharing mode='maximum'). Raw p values were adjusted for multiple testing with the Benjamini-Hochberg procedure (Benjamini and Hochberg, 1995). Genes or miRNAs with adjusted p-value of 0.05 or less were termed as differentially expressed and are listed in Table S1 and S5 respectively.

Comparison of seven day cut microarray to RNA-Seq datasets
There are 6009 genes that are DE in the uncut vs seven day cut microarray data with p-adj < 0.05, 4999 of which have ensemble IDs (Arthur-Farraj et al., 2012). This is compared to 3,176 DE RNAs from our uncut vs seven day cut RNA-Seq data. However, when we analyse DE genes (p-adj<0.05) with a fold

change >2 in the microarray, 84% (267/317) of downregulated genes and 77% of upregulated genes (326/422) were similarly regulated in our RNA-Seq dataset.

Enrichment Analysis of KEGG Pathways, Gene Ontologies and Protein Families
Enrichment of KEGG Pathways and Biological Processes for the 3331 DEG was calculated using DAVID (v6.8) (Dennis et al., 2003) with a background list of 23,121 genes identified in this study. Raw p-values were adjusted for multiple testing using the Benjamini-Hochberg procedure (Benjamini and Hochberg, 1995) and were deemed significant if the p-adj value was < 0.01.

mRNA-miRNA-TF Target predictions
A miRNA-mRNA target was accepted if at least two of the six programs employed on the miRWalk webserver (miRanda, miRDB, miRWalk, Targetscan, RNA22 and PITA) predicted a significant match ($p<0.05$), with a minimum seed length of seven within the 3'UTR region of the longest transcript. The predicted targets were compared against the list of DE genes identified between uncut and seven day cut nerves and genes common to both were tested for enrichment of pathways and gene ontologies. The miRNA gene target enrichment analysis using DAVID was set at p-adjusted value < 0.05. Magia^2 (Bisognin et al., 2012) was used to reconstruct a post-transcriptional regulatory network and transcription factors (TF) that regulate miRNA were determined. Both miRNA and RNA-Seq expression data was uploaded to Magia^2 (Bisognin et al., 2012) website and the Spearman correlation method alongside experimentally validated TF-miRNA interactions reported in mirGene2.0 (Friard et al., 2010) and Transmir (Wang et al., 2010) were used to determine the relationships between TFs and miRNAs. The following target predictors with mean thesholds were used to determine TF, miRNA, mRNA interactions; mirDB (threshold: 63), Microcos (threshold: 16.3), RNA22 (threshold: -27.5), DIANA microT (threshold 2.0), TargetScan (threshold: 0.7), Pita (threshold 0.2), PicTar (threshold: 3.8) and microrna.org (threshold: -0.3). P values were adjusted for multiple testing using FDR and the subsequent p-adjusted value was deemed significant if less than 0.10.

Enrichment analysis of EMT genes
A list of human EMT genes was obtained from the dbEMT database (Zhao et al., 2015). Mouse orthologs of the Ensembl listed EMT genes were identified (Ensembl v. 85). Of the 326 of the mouse to human orthologous EMT genes, 111 were significantly differentially expressed between cut and uncut nerve. A fishers exact test was performed to determine enrichment. No multiple correction was applied.
There were 20 mature miRNAs listed in the dbEMT database, of which mouse ortholgous miRNAs were identified through miRBase. Of the 17 mature miRNAs present in mouse nerve tissue, when taking into account the 3' and 5' arm of the miRNAs, there were a total of 29 miRNAs expressed in the mouse nerve. There were 14 of these miRNAs DE between cut and cut day three, 15 DE between uncut and cut at day seven, and 11 DE miRNAs between cut day three and cut day seven. A fishers exact test was performed to determine enrichment. No multiple correction was applied.

Identifying novel lncRNAs
To identify novel lncRNAs, cut and uncut bam file replicates were merged using SAMtools (Li et al., 2009) and RNA-Seq alignments were assembled into potential transcripts with StringTie (Pertea et al., 2015) with a minimum isoform fraction of 0.01. Uncut and cut GTF files were merged with Cuffcompare and transcripts were annotated and divided into those that had known Ensembl gene IDs and those that were novel. The protein coding potential of the novel transcripts was determined using Cpat (Wang et al., 2013), where a coding probability (CP) of 0.44 and a minimum length of 200bp was used to determine putative lncRNAs. To remove truncated transcripts resulting from post transcriptional modifications or degradation of RNA, additional quality filters were applied where lncRNAs were accepted if they had > 10 reads in one of the six samples and were expressed (reads > 0) in at least 3 samples. Lift over was used to convert the FANTOM5 mouse CAGE peak data (Forrest et al., 2014) from mm9 to mm10 and a 500 bp window +/- TSS of novel putative lncRNA was intersected these peaks to identify a set of high confidence lncRNAs (Fig. S2).

Detecting Differentially Methylated Bases
Illumina adapters were removed using paired option in trim_galore (v0.2.5) and reads with quality Phred score cut-off < 20 were removed. The overall quality of the sequence data was assessed for suitability using FastQC (v0.9.4)(www.bioinformatics.babraham.ac.uk/projects/fastqc). BSMAP (v2.74) (Xi and Li, 2009) was used to align reads to the mouse genome with quality threshold cut-off at 20, max mismatches at 8% and mapping strand information set to 0 where PE sequencing maps read1 to ++ and -+ strand and read2 to +- and -- strand. Reads were sorted, duplicates marked and removed, bam file indexed and lanes merged using PICARD tools (v1.107)(http://picard.sourceforge.net).

Methylation ratios were extracted from BSMAP mapping files using methratio.py from BSMAP (v2.74) (Xi and Li, 2009) where the minimum sequencing depth for reported loci was 10X and CpG methylation ratios were combined on both strands. Differential methylation was calculated using methylKit (Akalin et al., 2012) which calculates the p-values using logistic regression and these are subsequently adjusted to p-adjusted values using the sliding linear model (SLIM) method (Wang et al., 2011). Differentially methylated bases were then determined if the p-adjusted value was less than 0.05 and the percentage difference in methylation between samples was greater than 20%.

3. Supplemental References

Akalin, A., Kormaksson, M., Li, S., Garrett-Bakelman, F.E., Figueroa, M.E., Melnick, A., and Mason, C.E. (2012). methylKit: a comprehensive R package for the analysis of genome-wide DNA methylation profiles. Genome Biol. *13*, R87.

Anders, S., and Huber, W. (2010). Differential expression analysis for sequence count data. Genome Biol. *11*, R106.

Anders, S., Theodor, P., and Huber, W. (2015). HTSeq - A Python framework to work with high-throughput sequencing data. Bioinformatics, *31*: 166-169.

Arthur-Farraj, P., Wanek, K., Hantke, J., Davis, C.M., Jayakar, A., Parkinson, D.B., Mirsky, R., and Jessen, K.R. (2011). Mouse schwann cells need both NRG1 and cyclic AMP to myelinate. Glia *59*, 720–733.

Behmoares, J., Smith, J., D'Souza, Z., Bhangal, G., Chawanasuntoropoj, R., Tam, F.W., Pusey, C.D., Aitman, T.J., and Cook, H.T (2010). Genetic loci modulate macrophage activity and glomerular damage in experimental glomerulonephritis. J. Am. Soc. Nephrol. *21*, 1136-1144.

Benjamini, Y., and Hochberg, Y. (1995). Controlling the False Discovery Rate - a Practical and Powerful Approach to Multiple Testing. J. Roy. Stat. Soc. B. *57*, 289-300.

Bisognin, A., Sales, G., Coppe, A., Bortoluzzi, S., and Romualdi, C. (2012). MAGIA(2): from miRNA and genes expression data integrative analysis to microRNA-transcription factor mixed regulatory circuits (2012 update). Nuc. Acids Res. *40*, W13-21.

Dennis, G., Jr., Sherman, B.T., Hosack, D.A., Yang, J., Gao, W., Lane, H.C., and Lempicki, R.A. (2003). DAVID: Database for Annotation, Visualization, and Integrated Discovery. Genome Biol. *4*, P3.

Forrest, A.R., Kawaji, H., Rehli, M., Baillie, J.K., de Hoon, M.J., Haberle, V., Lassmann, T., Kulakovskiy, I.V., Lizio, M., Itoh, M. FANTOM Consortium and the RIKEN PMI and CLST (DGT). (2014). A promoter level mammalian expression atlas. Nature. *507*, 462-470.

Friard, O., Re, A., Taverna, D., De Bortoli, M., and Cora, D. (2010). CircuitsDB: a database of mixed microRNA/transcription factor feed-forward regulatory circuits in human and mouse. BMC bioinformatics *11*, 435.

Friedlander, M.R., Mackowiak, S.D., Li, N., Chen, W., and Rajewsky, N. (2012). miRDeep2 accurately identifies known and hundreds of novel microRNA genes in seven animal clades. Nuc. Acids Res. *40*, 37-52.

Kozomara, A., and Griffiths-Jones, S. (2011). miRBase: integrating microRNA annotation and deep-sequencing data. Nucleic Acids Res. *39*, D152-157.
Krueger, F.

Langmead, B., and Salzberg, S.L. (2012). Fast gapped-read alignment with Bowtie 2. Nat. Methods *9*, 357-359.

Li, H., Handsaker, B., Wysoker, A., Fennell, T., Ruan, J., Homer, N., Marth, G., Abecasis, G., Durbin, R. & Genome Project Data Processing. (2009). The Sequence Alignment/Map format and SAMtools. Bioinformatics. *25*, 2078-9.

Li, M.C., and Dahiya, R. (2002). MethPrimer: designing primers for methylation PCRs. Bioinformatics. *18*, 1427-1431.

Pertea, M., Pertea, G. M., Antonescu, C. M., Chang, T. C., Mendell, J. T. & Salzberg, S. L. (2015). StringTie enables improved reconstruction of a transcriptome from RNA-seq reads. Nat. Biotechnol. *33***,** 290-295.

Pope, B.D., Ryba, T., Dileep, V., Yue, F., Wu, W., Denas, O., Vera, D.L., Wang, Y., Hansen, R.S., Canfield, T.K., et al. (2014). Topologically associating domains are stable units of replication-timing regulation. Nature *515*, 402-405.

Stevens, B., Tanner, S., and Fields, R.D. (1998). Control of myelination by specific patterns of neural impulses. J. Neurosci. *18*, 9303-9311.

Trapnell, C., Pachter, L., and Salzberg, S.L. (2009). TopHat: discovering splice junctions with RNA-Seq. Bioinformatics *25*, 1105-1111.

Wang, H.Q., Tuominen, L.K., and Tsai, C.J. (2011). SLIM: a sliding linear model for estimating the proportion of true null hypotheses in datasets with dependence structures. Bioinformatics *27*, 225-231.

Wang, L., Park, H. J., Dasari, S., Wang, S., Kocher, J. P. & Li, W. (2013). CPAT: Coding-Potential Assessment Tool using an alignment-free logistic regression model. Nucleic Acids Res. *41***,** e74.

Woodhoo, A., Dean, C.H., Droggiti, A., Mirsky, R., and Jessen, K.R. The trunk neural crest and its early glial derivatives: a study of survival responses, developmental schedules and autocrine mechanisms. (2004). Mol. Cell Neurosci. *25*, 30-41.

Xi, Y., and Li, W. (2009). BSMAP: whole genome bisulfite sequence MAPping program. BMC Bioinformatics *10*, 232.

Ye, J., Coulouris, G., Zaretskaya, I., Cutcutache, I., Rozen, S., Madden, T. (2012). Primer-BLAST: A tool to design target-specific primers for polymerase chain reaction. BMC Bioinformatics *13*, 134.
