# Supplementary material for: Changes in the Coding and Non-coding Transcriptome and DNA Methylome that Define the Schwann Cell Repair Phenotype after Nerve Injury
Source: Cell Rep. 2017 Sep 12;20(11):2719–34. doi: 10.1016/j.celrep.2017.08.064 (PMC5608958; doi:10.1016/j.celrep.2017.08.064)
Supplement: Document S2. Article plus Supplemental Information [file mmc6.pdf]

Cell Reports

Resource

Changes in the Coding and Non-coding Transcriptome and DNA Methylome that Define the Schwann Cell Repair Phenotype after Nerve Injury

Graphical Abstract

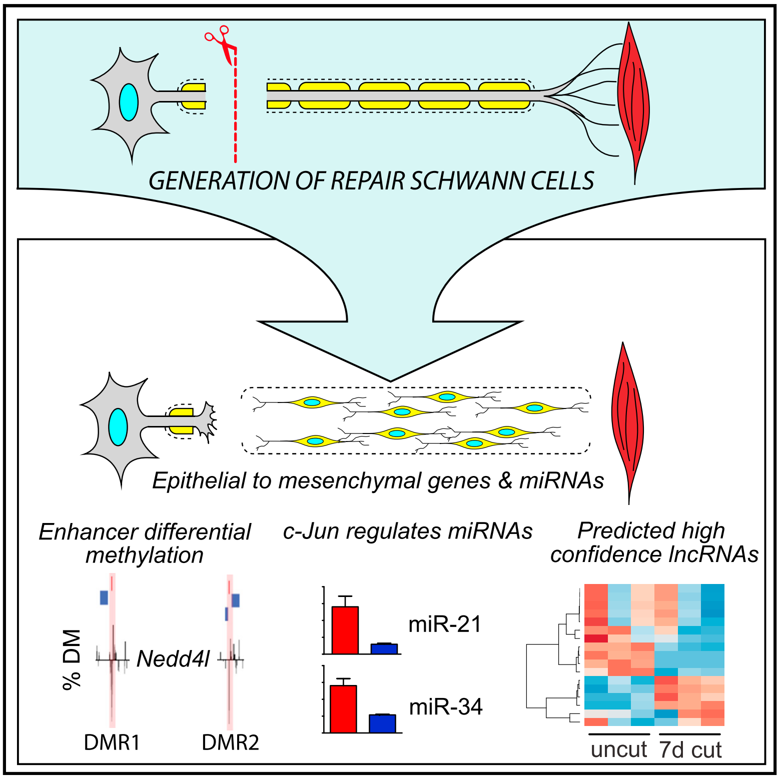

Highlights

- Epithelial-mesenchymal genes are enriched in the injured nerve
- Identification of differentially expressed lncRNAs following nerve injury
- c-Jun regulates specific microRNA expression after nerve injury
- Limited changes in CpG methylation following nerve injury

Authors

Peter J. Arthur-Farraj, Claire C. Morgan, Martyna Adamowicz, ..., Rhona Mirsky, Kristjan R. Jessen, Timothy J. Aitman

Correspondence

p.arthurfarraj@gmail.com (P.J.A.-F.), tim.aitman@ed.ac.uk (T.J.A.)

In Brief

Arthur-Farraj et al. report a combined transcriptome and whole-genome CpG methylation study in repair Schwann cells after nerve injury. They identify Schwann cell-expressed lncRNAs and miRNAs under the control of c-Jun, as well as differential methylation of enhancers of repair program genes.

Data and Software Availability

E-MTAB-5633

CrossMark
Arthur-Farraj et al., 2017, Cell Reports *20*, 2719–2734
September 12, 2017 © 2017 The Authors.
http://dx.doi.org/10.1016/j.celrep.2017.08.064

OPEN ACCESS
CellPress

Changes in the Coding and Non-coding Transcriptome and DNA Methylome that Define the Schwann Cell Repair Phenotype after Nerve Injury

Peter J. Arthur-Farraj,[1,2,3,5,6,*] Claire C. Morgan,[2,5] Martyna Adamowicz,[4,5] Jose A. Gomez-Sanchez,[3] Shaline V. Fazal,[3] Anthony Beucher,[2] Bonnie Razzaghi,[2] Rhona Mirsky,[3] Kristjan R. Jessen,[3] and Timothy J. Aitman[2,4,*]
[1]Department of Clinical Neurosciences, Addenbrooke's Hospital, University of Cambridge, Cambridge CB2 0QQ, UK
[2]Department of Medicine, Imperial College, London W12 0NN, UK
[3]Department of Cell and Developmental Biology, University College London, London WC1E 6BT, UK
[4]Centre for Genomic and Experimental Medicine, MRC Institute of Genetics and Molecular Medicine, University of Edinburgh, Edinburgh EH16 2XU, UK
[5]These authors contributed equally
[6]Lead Contact
*Correspondence: p.arthurfarraj@gmail.com (P.J.A.-F.), tim.aitman@ed.ac.uk (T.J.A.)
http://dx.doi.org/10.1016/j.celrep.2017.08.064

SUMMARY

Repair Schwann cells play a critical role in orchestrating nerve repair after injury, but the cellular and molecular processes that generate them are poorly understood. Here, we perform a combined whole-genome, coding and non-coding RNA and CpG methylation study following nerve injury. We show that genes involved in the epithelial-mesenchymal transition are enriched in repair cells, and we identify several long non-coding RNAs in Schwann cells. We demonstrate that the AP-1 transcription factor C-JUN regulates the expression of certain micro RNAs in repair Schwann cells, in particular miR-21 and miR-34. Surprisingly, unlike during development, changes in CpG methylation are limited in injury, restricted to specific locations, such as enhancer regions of Schwann cell-specific genes (e.g., *Nedd4l*), and close to local enrichment of AP-1 motifs. These genetic and epigenomic changes broaden our mechanistic understanding of the formation of repair Schwann cell during peripheral nervous system tissue repair.

INTRODUCTION

Schwann cells in the peripheral nervous system (PNS) play a crucial role in the repair of injured nerves (Jessen and Mirsky, 2016). In response to injury, the myelin and non-myelin (Remak) Schwann cells that normally ensheath undamaged axons undergo extensive molecular and cellular changes to generate a distinct Schwann cell phenotype, the repair Schwann cell. This cell is specialized for maintaining survival of injured neurons, supports axonal regeneration, and is essential for functional nerve repair. Formation of repair Schwann cells requires the downregulation of genes involved in myelination and upregulation of an injury-specific program of gene expression (Arthur-Farraj et al., 2012; Fontana et al., 2012; Jessen and Mirsky, 2016).

Repair Schwann cells express key regeneration promoting genes, such as neuronal growth factors and cell adhesion molecules; break down redundant myelin sheaths by activating myelin autophagy and recruiting macrophages; and by adopting a slender, elongated morphology, they form regeneration tracts, called bands of Bungner, which help guide axons back to their targets (Gomez-Sanchez et al., 2015; Jessen and Mirsky, 2016). Repair Schwann cell formation is regulated by the AP-1 transcription factor (TF) C-JUN; however, several other factors have subsequently been shown to regulate the Schwann cell response to nerve injury (Arthur-Farraj et al., 2012; reviewed in Boerboom et al., 2017).

The present study explores the involvement of epigenetic mechanisms in the generation of repair Schwann cells after nerve injury, in particular the roles of non-coding RNA and DNA methylation in gene expression and subsequent phenotype (Bonasio et al., 2010). We describe two functional groups of non-coding RNAs, microRNAs (miRNAs), which are 21- to 24-nucleotide regulatory RNAs, and long non-coding RNAs (lncRNAs), which are defined as RNA molecules greater than 200 nucleotides in length with no coding potential (Quinn and Chang, 2016; Sabin et al., 2013). Both miRNAs and global DNA methylation changes have already been shown to have a role in Schwann cell development (Gökbuget et al., 2015; Varela-Rey et al., 2014). After nerve injury, disruption of miRNA processing in Schwann cells results in reduced remyelination in regenerating nerves, but knowledge about the roles or regulation of specific miRNAs is limited (Viader et al., 2011; Zhou et al., 2016). Furthermore, little is known about lncRNA expression and the DNA methylation changes that occur after nerve injury.

Here, we present a large-scale combined study of changes in the coding and non-coding transcriptome and methlyome in response to PNS injury and detail our findings below.

RESULTS

To determine the changes in the transcriptome and methylome in repair Schwann cells after nerve injury, we performed sciatic nerve cuts in 6- to 8-week-old male C57BL/6J mice and

This is an open access article under the CC BY license (http://creativecommons.org/licenses/by/4.0/).

OPEN ACCESS
CellPress

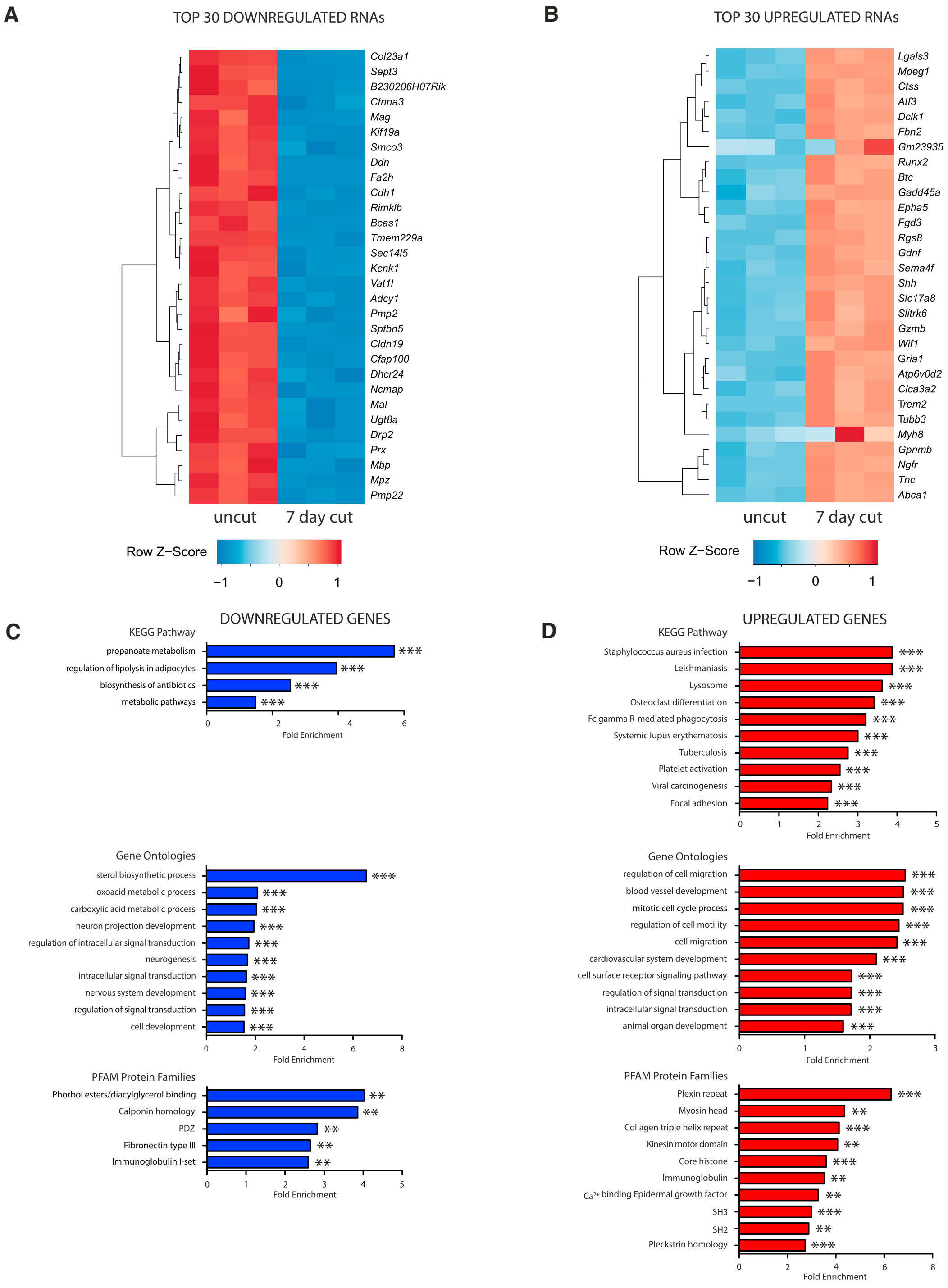

E

DOWNREGULATED EMT-ASSOCIATED GENES

Gene Name	Long Name	FC uncut v 7d cut	p-adj
Cdh1	*E-Cadherin*	-7.21	1.24E-93
Wt1	*Wilm's tumour protein 1*	-4.62	2.75E-05
Fgf1	*Fibroblast growth factor 1*	-4.11	2.30E-79
Rgcc	*Regulator of the cell cycle*	-3.53	8.91E-20
Esrp2	*Epithelial splicing regulatory protein 2*	-3.03	0.00035
Col8a2	*Collagen type 8 alpha 2 chain*	-2.95	1.36E-17
Ndrg1	*N-myc downstream regulated 1*	-2.49	1.47E-45
Smad9	*SMAD family member 9*	-2.47	0.042
Gsn	*Gelsolin*	-2.25	4.70E-36
Lox	*Lysyl oxidase*	-2.19	7.48E-24

UPREGULATED EMT-ASSOCIATED GENES

Gene Name	Long Name	FC uncut v 7d cut	p-adj
Shh	*Sonic Hedgehog*	186.97	5.71E-57
Tnc	*Tenascin C*	69.13	6.1E-155
Hmga2	*High mobility group AT-hook 2*	24.50	7.55E-22
Timp1	*TIMP metallopeptidase inhibitor 1*	17.10	6.28E-18
Igfbp3	*Insulin-like growth factor binding protein 3*	9.11	1.33E-41
Hpse	*Heparanase*	6.91	2.46E-13
Cxcr4	*C-X-C chemokine receptor 4*	6.55	1.54E-15
Tgfb1	*Transforming growth factor beta 1*	6.38	4.38E-09
Sema3e	*Semaphorin 3e*	6.11	7.91E-18
Met	*MET proto-oncogene receptor tyrosine kinase*	6.05	1.06E-39

(legend on next page)

harvested the distal stump for either RNA sequencing (RNA-seq) or whole-genome shotgun bisulfite sequencing (WGSB-seq) 7 days after nerve cut. For miRNA analysis, we also performed small RNA-seq on sciatic nerve distal stump tissue 3 and 7 days after cut. The contralateral uninjured sciatic nerve was the control in all experiments (referred to as uncut throughout this article). Sequencing experiments were performed with a minimum of three biological replicates. Results of RNA-seq, small RNA-seq, and WGSB-seq were confirmed on uncut and cut nerve samples by qPCR and Sanger sequencing, respectively. In order to investigate cell-specific coding and non-coding RNA expression and CpG methylation changes, we also performed qPCR and Sanger sequencing on cultured purified Schwann cells, nerve-derived fibroblasts, and bone-marrow-derived macrophages activated with lipopolysaccharide (LPS).

RNA-Seq Analysis Shows Enrichment of Genes Involved in Epithelial to Mesenchymal Transition in the Injured Nerve

From our RNA-seq analysis, we identified 3,176 differentially expressed (DE) coding and non-coding RNAs between uncut and 7-day cut mouse sciatic nerves (Table S1; Figures S1A and S1B). These results correlated well with our previously published microarray data (Arthur-Farraj et al., 2012), where 80% of significantly DE genes (fold change >2, p-adj < 0.05) from the microarray were also significant and in the same direction in our RNA-seq dataset (see Supplemental Experimental Procedures). Among the top 30 most downregulated RNAs, reassuringly, we identified a number of known myelin-associated genes, such as *Mbp*, *Pmp22*, *Mpz*, *Prx*, *Drp2*, and *Cdh1* (Figure 1A). Similarly, among the top 30 most upregulated RNAs 7 days after nerve injury were several well-known repair program genes, such as *Ngfr*, *Lgals3*, *Atf3*, *Shh*, and *Gdnf* (Arthur-Farraj et al., 2012; Figure 1B). Out of all DE RNAs, we selected mainly upregulated RNAs to validate by qPCR based on their potential roles in repair cells identified from literature searches and Kyoto Encyclopedia of Genes and Genomes (KEGG) pathway and protein family analysis (Figures 1C and 1D; Table S2A). Myelin genes and known repair program genes were used as controls. In total, we successfully validated 36 out of these 37 RNAs by qPCR on uncut and 7-day cut nerves. These included the main AP-1 TF members, four lncRNAs, and repair cell genes with potential roles in extracellular matrix (ECM) remodeling, axon growth and intracellular signaling (Table S2A). Although the majority of cells in uninjured and injured nerves are Schwann cells (Table S2C), we wanted to check the relative expression of putative repair program RNAs in the major different cells types found within the injured nerve. As cultured Schwann cells closely replicate the gene expression of repair Schwann cells in vivo, they make a valid in vitro assay for repair cells (Arthur-Farraj et al., 2012). Using purified cultures of Schwann cells, nerve fibroblasts, and macrophages, we found that the large majority of putative repair program coding and non-coding RNAs (24 out of 33) we tested were significantly more highly expressed in Schwann cells than in fibroblasts or macrophages (Table S2B).

To observe the broader regulation of genes after nerve injury, we performed KEGG pathway, gene ontology (GO), and protein family enrichment analysis (PFAM). Among downregulated genes, this revealed enrichment in the sterol biosynthetic processes and regulation of lipolysis and significant enrichment for genes belonging to diacylglycerol-binding, calponin homology, and PDZ-domain-containing protein families (Figure 1C). Within upregulated genes, there was enrichment of lysosome, osteoclast differentiation, focal adhesion, and inflammatory pathways along with biological processes involving the cell cycle, migration, signal transduction, and cell surface receptor signaling. Furthermore, there was enrichment of genes belonging to various protein families, including collagens, plexins, myosins, and calcium-binding epidermal growth factor domains (Figure 1D). These findings emphasize the importance of gene expression changes after nerve injury in remodeling of the ECM, changes in the actin cytoskeleton, and changes in intracellular signaling in repair Schwann cells.

The morphological transition from a differentiated Schwann cell into a repair Schwann cell is similar to epithelial-mesenchymal transition (EMT), which has well-known roles in oncogenesis and wound healing (Lamouille et al., 2014). Myelin Schwann cells have previously been likened to epithelial cells (Bunge and Bunge, 1983), as they have a basement membrane and cell polarity, with an abaxonal and adaxonal membrane, and express typical epithelial markers such as E-cadherin, claudins, and polarity proteins such as PAR3 (Chan et al., 2006; Crawford et al., 2008; Table S1). After nerve injury, Schwann cells lose their abaxonal and adaxonal polarity, re-enter the cell cycle, and adopt migratory behavior at the site of injury (Jessen and Mirsky, 2016).

To test whether genes involved in EMT were substantially regulated after nerve injury, we utilized the list of known human EMT genes in the dbEMT database and identified the corresponding mouse orthologs (Zhao et al., 2015). Out of the 326 mouse-to-human orthologous EMT genes, 111 were significantly DE between uncut and 7-day cut nerves, and a Fisher's exact test confirmed enrichment ($p < 2.2E-16$; odds ratio [OR], 3.14; 95% confidence interval [CI], 2.46, 3.97; Table S3). Of the top 10 most upregulated EMT genes, there were mRNAs known to be specifically expressed in repair Schwann cells, such as *Shh*, *Tnc*, and *Tgfb1* (Arthur-Farraj et al., 2012; Martini et al., 1990; Scherer et al., 1993; Figure 1E; Table S3). Furthermore, we also found that there was significant enrichment of EMT genes within the subset of Schwann cell/*c-Jun*-dependent genes identified from our previous microarray (11/172 genes; OR, 14.58; $p = 1.39E-09$; Arthur-Farraj et al., 2012). These

Figure 1. RNA-Seq Analysis Identifies Enrichment of EMT Genes after Nerve Injury

(A) A heatmap of the top 30 significantly downregulated genes between uncut and 7-day cut nerves (n = 3, adjusted p value [p-adj] < 0.05).

(B) A heatmap of the top 30 significantly upregulated genes between uncut and 7-day cut nerves (n = 3, p-adj < 0.05).

(C and D) Enriched KEGG pathways, GO terms, and protein families (PFAM) for (C) downregulated genes and (D) upregulated genes from RNA-seq analysis 7 days after nerve cut compared to the uncut nerve (n = 3). *p-adj < 0.05; **p-adj < 0.01; ***p-adj < 0.001.

(E) Enrichment analysis of EMT genes from the RNA-seq study showing the 10 most downregulated and upregulated mRNAs (p-adj < 0.05).

OPEN
ACCESS
CellPress

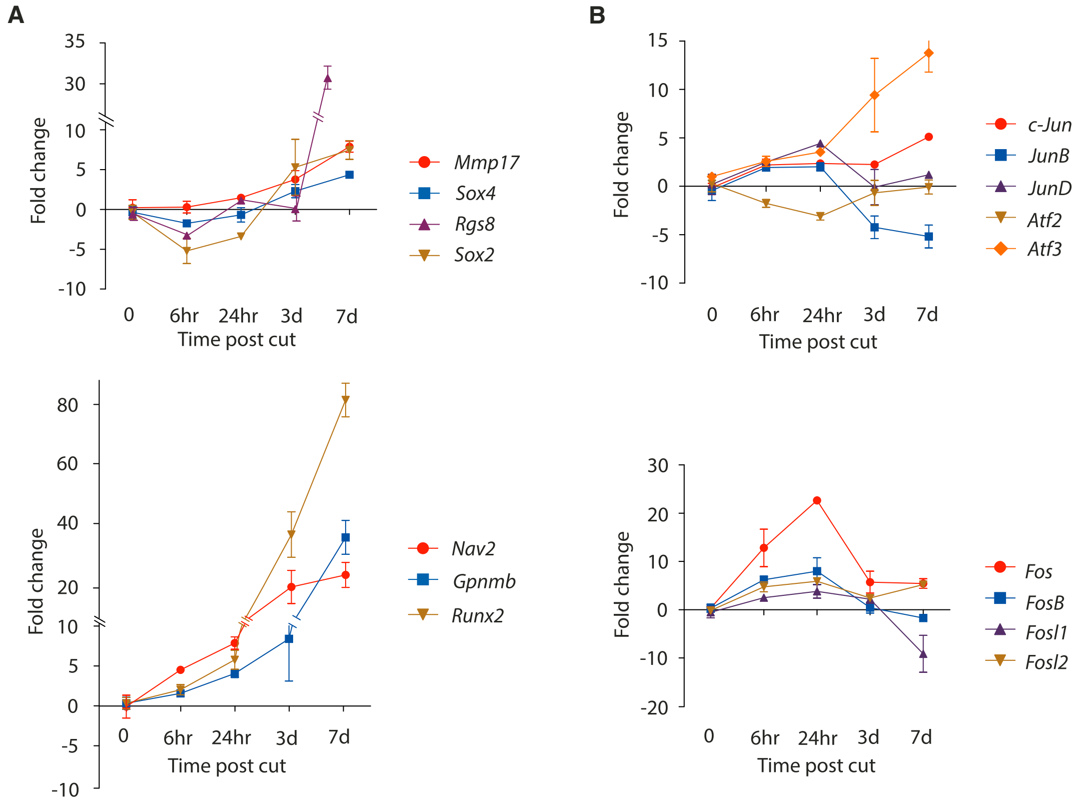

C

Gene	24hr cut FC	p-adj value
Atf2	-3.01	0.08 NS
Atf3	3.53	1.76E-05
Fos	22.64	4.03E-05
FosB	7.99	0.15 NS
Fosl1	3.78	0.09 NS
Fosl2	5.90	1.89E-05
Gpnmb	4.06	0.04
c-Jun	2.35	1.04E-03
JunB	2.01	0.12 NS
JunD	4.42	0.003
Mmp17	1.47	0.33 NS
Nav2	7.87	6.8E-03
Rgs8	1.12	0.11 NS
Runx2	5.78	0.04
Sox2	-3.29	0.07 NS
Sox4	-1.24	0.79 NS

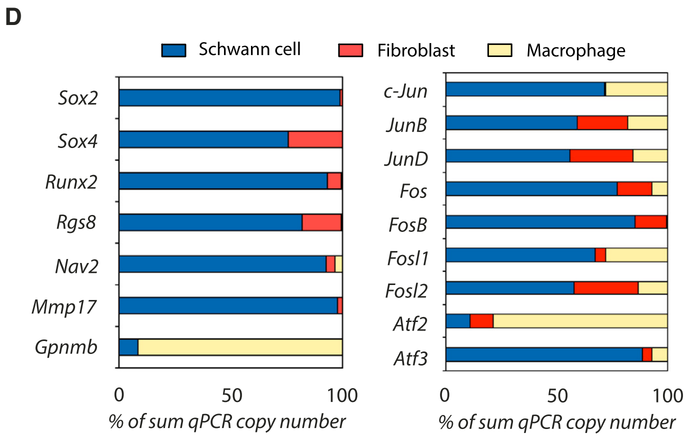

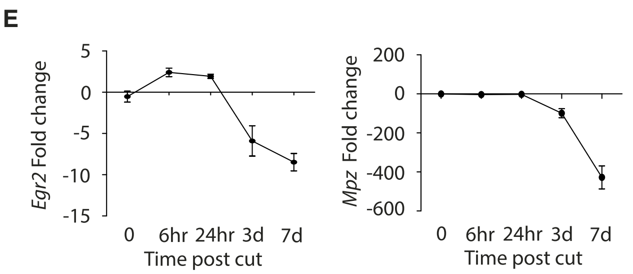

F

Gene	24hr cut FC	p-adj value
Egr2	1.93	0.06 NS
Mpz	-2.18	0.30 NS

Figure 2. Expression Patterns for Putative Schwann Cell Repair Program Genes and AP-1 TFs after Nerve Injury

(A) Time course of putative repair program gene expression after sciatic nerve cut by qPCR (n = 5).

(B) Time course of expression of members of the AP-1 TF family after sciatic nerve cut by qPCR (n = 5). Fold change in (A) and (B) is relative to the uncut nerve.

(legend continued on next page)

results show that a significant proportion of the molecular machinery involved in EMT is also regulated during the formation of repair Schwann cells after nerve injury.

Fast Activation of AP-1 TFs in Repair Schwann Cells

c-Jun mRNA and protein are upregulated within the first 24 hr after nerve injury, and it has been suggested that this fast rate of upregulation may be important for the strong effects of Schwann cell C-JUN on nerve regeneration (Arthur-Farraj et al., 2012; Painter et al., 2014; Parkinson et al., 2008). We determined the time course of expression for a number of repair program genes that we validated from the RNA-seq results, including *c-Jun* and other members of the AP-1 TF family. There was little change or reduction in the expression of several repair program genes, such as *Rgs8*, *Sox2*, and *Sox4*, during the first 3 days after cut, followed by a linear increase up to day 7 (Figure 2A). However, other repair program genes, such as *Nav2* and *Runx2*, and many of the AP-1 genes followed a different expression pattern, showing an early peak of mRNA expression ~24 hr after nerve injury (Figures 2A–2C). Importantly, 20 out of 22 genes examined in this set of experiments were significantly more highly expressed in cultured Schwann cells than in nerve fibroblasts or macrophages. Only *JunB* and *Gpnmb*, while expressed in Schwann cells, were more highly expressed in macrophages (Figure 2D; Table S2B).

Interestingly, the fast activation of repair program genes was not accompanied by downregulation of myelin genes, as both *Mpz* and *Egr2* mRNA were still robustly expressed at 24 hr post-cut and only significantly downregulated at 3 days post-cut (Figures 2E and 2F). This indicates that transcriptional activation of the repair program starts very soon after injury, before the TFs that control myelination are extinguished and, crucially, before axonal degeneration has taken place, which happens at 36–44 hr in mouse sciatic nerves (Conforti et al., 2014). Therefore, the transcriptional regulators of both the myelin program and the repair program may be active in Schwann cells at early time points after nerve injury.

Expression Patterns of Non-coding RNAs in Repair Schwann Cells

Between uncut and 7-day cut samples, we identified 52 known DE lncRNAs out of a total of 1,533 annotated lncRNAs. Of these 52, 19 were upregulated and 33 were downregulated (Figure 3A; Table S1). Additionally, we identified 913 predicted high-confidence lncRNAs, 433 antisense and 480 intergenic, of which 17 were DE (11 up and 6 down), 7 days after nerve cut (Figures 3B and S2; Table S1). Interestingly, a number of known and predicted lncRNAs were located near genes that were also DE after nerve injury. These included *H19* and *Igf2*, *Pvt1* and *Myc*, *Gm12688* and *Foxd3*, *Sox2ot* and *Sox2*, *GM16083* and *Cd55*, *Sap30bpos* and *Itgb4*, *STGIG035960* and *Slc15a3*, and *STGIG026815* and *Wnt10b* (Figure 3C). We validated expression of four lncRNAs upregulated after nerve injury by qPCR (Figure 3D; Table S2A). We then used cell culture to examine the cell-type expression of these lncRNAs and found that *Sox2ot* and *H19* were both preferentially expressed in Schwann cells. *Rian* and *Meg3* were also expressed in Schwann cells, although they were found at higher levels in nerve fibroblasts. There was negligible expression of these four lncRNAs in macrophages (Figure 3E; Table S2B).

We identified a total of 397 DE miRNAs between uncut and cut samples from our small RNA-seq analysis. We analyzed a 3-day time point, in addition to 7 days post-cut, as earlier time points had previously been shown to have significant miRNA regulation (Viader et al., 2011; Adilakshmi et al., 2012) (Figure 4A; Table S4). We found similar numbers of DE miRNAs at 3 days and 7 days post-cut when both were compared to uncut nerve. Specifically, we found 237 DE miRNAs between uncut and 3-day cut samples, of which 113 were upregulated and 124 were downregulated. Between uncut and 7-day cut samples, we identified 239 DE miRNAs, of which 114 were upregulated and 125 were downregulated. Comparisons between 3-day and 7-day cut samples revealed 257 DE miRNAs, with 127 significantly upregulated and 130 downregulated (Figures 4B–4D; Table S4). Six miRNAs (miR-146b, miR-383-5p, miR-34c-5p, miR-96-5p, miR-183-5p, and miR-182-5p) were among the top 40 regulated miRNAs in all three comparisons, and miR-21a-5p and miR-34b-3p were among the top three most upregulated miRNAs in 3-day and 7-day cut nerves (Figures 4B–4D; Table S4). We successfully validated 15 miRNAs (7 upregulated and 8 downregulated) on uncut and 3-day and 7-day cut samples using qPCR (Figures S3A and S3B).

To test whether miRNAs involved in EMT were regulated after nerve cut, we again used the dbEMT database to obtain a list of known human EMT miRNAs and identified the corresponding mouse orthologs (Zhao et al., 2015). We found a significant enrichment of miRNAs involved in EMT. 15 out of 20 mouse orthologous miRNAs with a role in EMT were DE between uncut and 7-day cut samples from our small RNA-seq analysis; this included several members of the miR-21 and miR-34 families ($p < 0.013$; OR, 2.61; 95% CI, 1.15, 5.94; Figure 4E; Table S3).

Six of the seven validated upregulated miRNAs after nerve injury were expressed in cultured Schwann cells (Figure S3C). miR-17-5p and miR-362-3p showed the highest relative expression in Schwann cells, while miR-142-3p was expressed specifically in cultured macrophages. However, expression levels of miR-21-3p, miR-34a-5p, miR-34b-5p, miR34c-5p, and miR-132-3p were higher in nerve fibroblasts than in Schwann cells (Figure S3C).

Using the miRWalk webserver, we identified a set of miRNAs that target genes associated with some of the enriched KEGG pathways, gene ontologies, and protein families from

(C) Table showing fold change values of repair program genes and all AP-1 TFs 24 hr post-cut relative to uncut nerve. p-adj values are displayed.
(D) Relative cell type expression of repair program genes and AP-1 TFs in cultured mouse Schwann cells (blue), nerve fibroblasts (red), and activated macrophages (yellow) displayed as percentage of the sum of qPCR copy-number values from 1ug of RNA from each of the three cell types (n = 3).
(E) Time course of expression of *Egr2* and *Mpz* by qPCR after sciatic nerve cut. Fold change is relative to the uncut nerve (n = 5).
(F) Table showing fold change values of myelin program genes, *Egr2* and *Mpz*, 24 hr post-cut relative to uncut nerve, with p-adj displayed.
Error bars in all graphs represent SEM.

OPEN ACCESS
CellPress

A

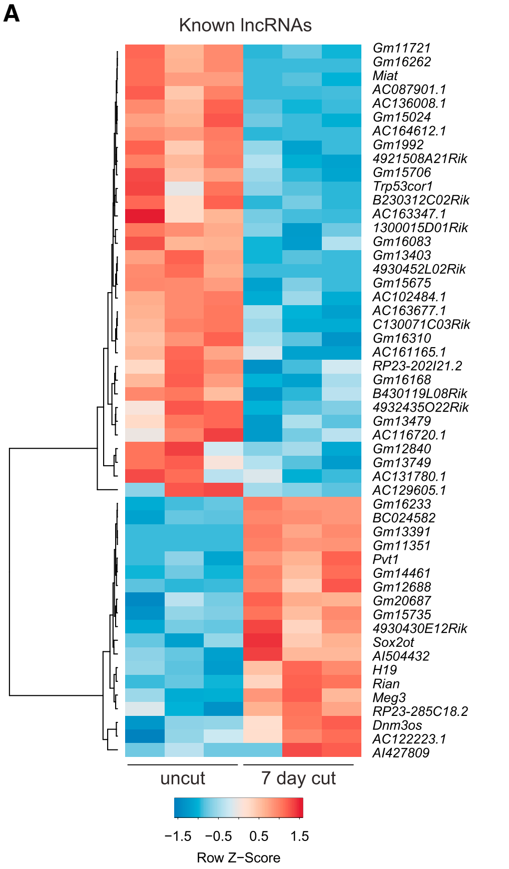

C i

Known LncRNA	FC	P-adj	Closest Genes	DE?	FC
Gm14461	232.73	2.67E-08	*Ube2e3*(-631107)	N	
			Cwc22(-291565)	N	
AI427809	55.70	0.04	*Slc44a1*(-176627)	Y	-1.64
			Abca1(-103891)	Y	16.57
Bach2os	48.70	3.18E-04	*Gja10*(+31137)	N	
			D130062J21Rik(+327890)	N	
H19	7.91	2.91E-38	*Mrpl23*(+44474)	N	
			Igf2(+82003)	Y	5.19
Pvt1	4.32	6.77E-04	*Myc*(+52703)	Y	2.77
Meg3	2.43	9.63E-09	*Rtl1*(+59280)	N	
			Dlk1(+88201)	Y	19.47
Gm12688	2.23	0.04	*Foxd3*(-1349)	Y	4.61
Sox2ot	1.78	0.03	*Sox2*(-545500)	Y	9.83
			Dnajc19(-23193)	N	
9530026P05Rik	-3.12	5.47E-04	*Adamts9*(-39148)	Y	2.53
Gm15706	-5.44	5.79E-03	*Kras*(-420)	Y	-3.26
Gm16083	-8.08	4.84E-03	*Cd55*(+117)	Y	-5.40
Gm13479	-15.85	0.04	*Zeb2*(-244020)	N	
Sap30bpos	-25.52	2.79E-09	*Itgb4*(-22650)	Y	-2.36
			Recql5(-18598)	N	
4932435O22Rik	-84.96	1.84E-06	*Sdk2*(-382548)	Y	-11.32
			Rpl38(-219843)	N	
B230312C02Rik	-220.52	1.05E-03	*Slco4a1*(-75150)	N	
			Gata5(-51130)	N	

ii

Predicted LncRNA	FC	P-adj	Closest Genes	DE?	FC
STGIG017703	54.64	0.01	*Slc22a23*(-336)	Y	7.56
STGIG069312	45.29	0.05	*4930555F03Rik*(-527231)	N	
			Tenm3(-168992)	Y	8.26
STGASG035960	26.36	0.01	*Tmem132a*(+10029)	Y	2.86
			Slc15a3(+17367)	Y	32.31
STGIG026815	22.04	0.03	*Wnt10b*(-278)	Y	3.28
STGASG028957	3.37	5.93E-18	*Filip1l*(+38366)	N	
			Cmss1(+215366)	N	
STGIG025081	1.20	0.04	*Tbc1d22a*(-19580)	N	
			Cerk(-8738)	Y	2.32
STGASG028349	-2.69	0.01	*Txndc11*(-9437)	N	
			Zc3h7a(+32306)	N	
STGASG076617	-2.72	0.02	*Limd1*(-16707)	N	
			Lars2(+95054)	N	
STGASG027573	-2.82	7.13E-04	*Kpna1*(-1610)	N	
STGASG039934	-2.90	8.60E-09	*Gm10800*(+260)	Y	-8.13
STGIG068665	-4.50	6.32E-07	*Cd209a*(-5519)	N	
			Cd209e(+99806)	N	
STGIG005361	-4.66	1.10E-04	*Gm10797*(-2843)	N	
STGASG027814	-4.82	3.19E-09	*Filip1l*(+38250)	N	
			Cmss1(+215482)	N	
STGASG039933	-7.25	1.33E-04	*Gm10800*(+616)	Y	-8.13
STGIG074680	-29.13	2.22E-03	*Topaz1*(-64336)	N	
			Abhd5(+331402)	Y	-1.82

B

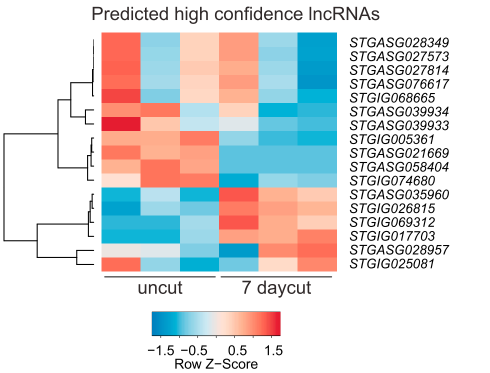

D

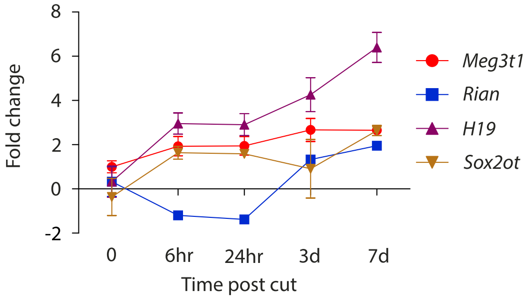

E

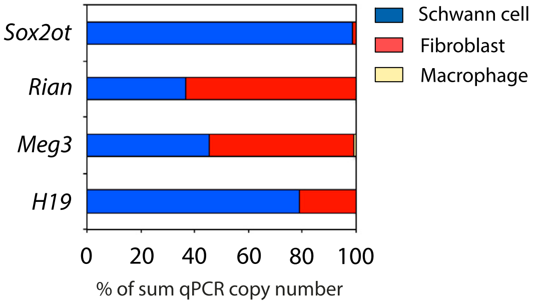

(legend on next page)

our RNA-seq study (Figures 1 and S3D). For instance, two miRNAs belonging to the miR29 family (miR-29a and miR-29c) and two members of the miR-154 family (miR-409 and miR-494) were both predicted to target genes enriched for the focal adhesion pathway, and miR29 family members are also predicted to target collagen protein families (Figure S3D).

These findings demonstrate that there is significant regulation of non-coding RNAs in the injured peripheral nerve. In particular, the lncRNAs *H19* and *Sox2ot* are highly expressed in cultured Schwann cells. However, we have also identified that a number of lncRNAs and miRNAs are also highly expressed in nerve fibroblasts. This suggests that other cells types, in addition to Schwann cells, are likely to contribute to non-coding RNA expression patterns in the injured nerve.

c-Jun Regulates the Expression of miRNA in Repair Schwann Cells

In silico interactions among miRNAs, mRNAs, and TFs were calculated using six independent predictor methods. In this way, we identified *c-Jun* and *Foxd3* as potential key regulators of miRNA expression (Figure S3E). We analyzed the expression of the 15 validated injury-regulated miRNAs in cut nerves of a Schwann cell-specific knockout of *c-Jun* (Arthur-Farraj et al., 2012). We found no significant differences in expression of four miRNAs, which were predicted to be regulated by *c-Jun*, between cut *c-Jun flox/flox* control nerves and *c-Jun*-null nerves (Figure S2F). However, we did identify six miRNAs that were DE between 7-day cut control and *c-Jun* null nerves. Both miR-21a-5p and miR-34b were expressed at significantly lower levels in *c-Jun*-null cut nerves than in controls, suggesting that C-JUN may be important for their upregulation after nerve injury. In contrast, miR96-5p, miR-124-3p, miR-183-5p, and miR-204-5p were more highly expressed in *c-Jun*-null cut nerves than in controls, indicating that C-JUN may downregulate expression of these miRNAs after nerve injury (Figure 4F). These results show that the *c-Jun*-dependent control of Schwann cell reprogramming extends to the regulation of appropriate miRNA levels in injured nerves.

CpG Methylation Changes in Repair Schwann Cells after Nerve Injury

For analysis of CpG methylation changes in injured nerves, we performed WGSB-seq. First, we identified 4,221,750 methylated CpG sites that had >5× coverage (meaning that each CpG site was sequenced at least five times) across the genome. Validation of the WGBS-seq data was obtained by locus-specific bisulfite-Sanger sequencing ($r^2 = 0.88$) at 20 individual CpGs (Figures 5A and S4A–S4C). Out of these 4,221,750 CpGs, only 853 were significantly differentially methylated (DM) between uncut and 7-day cut samples (adjusted p value [p-adj] < 0.05; differential methylation difference >20%) (Table S5; Figure S4D). Since biologically significant alterations in CpG methylation often affect adjacent CpGs, we clustered together ≥2 DM CpGs within 500 bp of one another and identified 46 unique differentially methylated regions (DMR) (Figure S4E). Significant enrichment of individual DM CpGs and DMRs occurred within gene regulatory regions, in particular introns and enhancers (Figures 5B, 5C, and S4D–S4F). Importantly, this enrichment was independent of technical variation in CpG read coverage (Figure S4G). DM CpGs were predominantly within 500 kb of transcription start sites (TSSs), indicating their *cis*-regulatory potential (Figure 5D). Next, we investigated whether the DM in the nerve is a result of Schwann cell-specific changes. We assessed percentage total methylation at individual CpGs in six of the most DM CpG clusters, identified from our WGSB-seq analysis. We then compared this to the percentage methylation of the same CpGs in cultured Schwann cells, nerve-derived fibroblasts, and activated macrophages using Sanger sequencing. We found CpG methylation levels in Schwann cells correlated strongly with levels of CpG methylation in the injured nerve, whereas no significant correlation was found for macrophages and fibroblasts with injured nerve samples (Figures 5E–5G).

The expression of myelin genes is reduced in adulthood relative to that seen during active myelination in developing nerves, and this reduction is accompanied by increased methylation of promoters and enhancer regions of myelin genes (Varela-Rey et al., 2014). Downregulation of myelin genes also takes place after injury, when adult expression levels decline to the very low levels seen in repair Schwann cells. However, comparing uncut nerves with 7-day cut nerves, we observed barely any CpG (between 0.0003% and 0.0005%) DM in putative regulatory regions within myelin-associated gene boundaries (determined by topological associated domains; see Supplemental Experimental Procedures) (Figure 5H). Surprisingly, therefore, myelin gene downregulation associated with development and after injury appear to be regulated differently, with CpG methylation having a reduced role in the injury response.

CpG methylation has previously been suggested to influence TF binding to target sites in DNA (Domcke et al., 2015; Schmidl et al., 2009). To investigate whether DM was occurring in or around specific TF-binding sites, we performed a de novo motif search within a 40-bp window centered on each of the 853 DM CpGs. In particular, we identified AP-1, *Atf3*, and *Fosl2* motifs as significantly enriched (Figure 5I). Both *Atf3* and *Fosl2* are AP-1 family members, along with *c-Jun*. These results demonstrate that significant changes in DNA methylation occur near

Figure 3. lncRNA Expression after Nerve Injury

(A) A heatmap of the 52 annotated DE lncRNAs between uncut and 7-day cut sciatic nerve from RNA-seq experiments (p-adj < 0.05).

(B) A heatmap of the 17 predicted DE lncRNAs between uncut and 7-day cut sciatic nerve from RNA-seq experiments (p-adj < 0.05).

(C) Table of selected known (i) and predicted (ii) lncRNAs, with fold change (FC) and p-adj values from the RNA-seq study along with their closest genes, whether they are also differentially expressed (DE?) in the RNA-seq study, and the corresponding fold change.

(D) Time course of expression of the lncRNAs, *Meg3* (t1 = transcript 1), *Rian*, *H19*, and *Sox2ot* after sciatic nerve cut by qPCR (n = 4). Fold change is relative to the uncut nerve. Error bars represent SEM.

(E) Relative cell type expression of *Meg3* (transcript 1), *Rian*, *H19*, and *Sox2ot* in cultured mouse Schwann cells (blue), nerve fibroblasts (red), and activated macrophages (yellow) displayed as percentage of the sum of qPCR copy-number values from 1ug of RNA from each of the three cell types (n = 3).

OPEN
ACCESS
CellPress

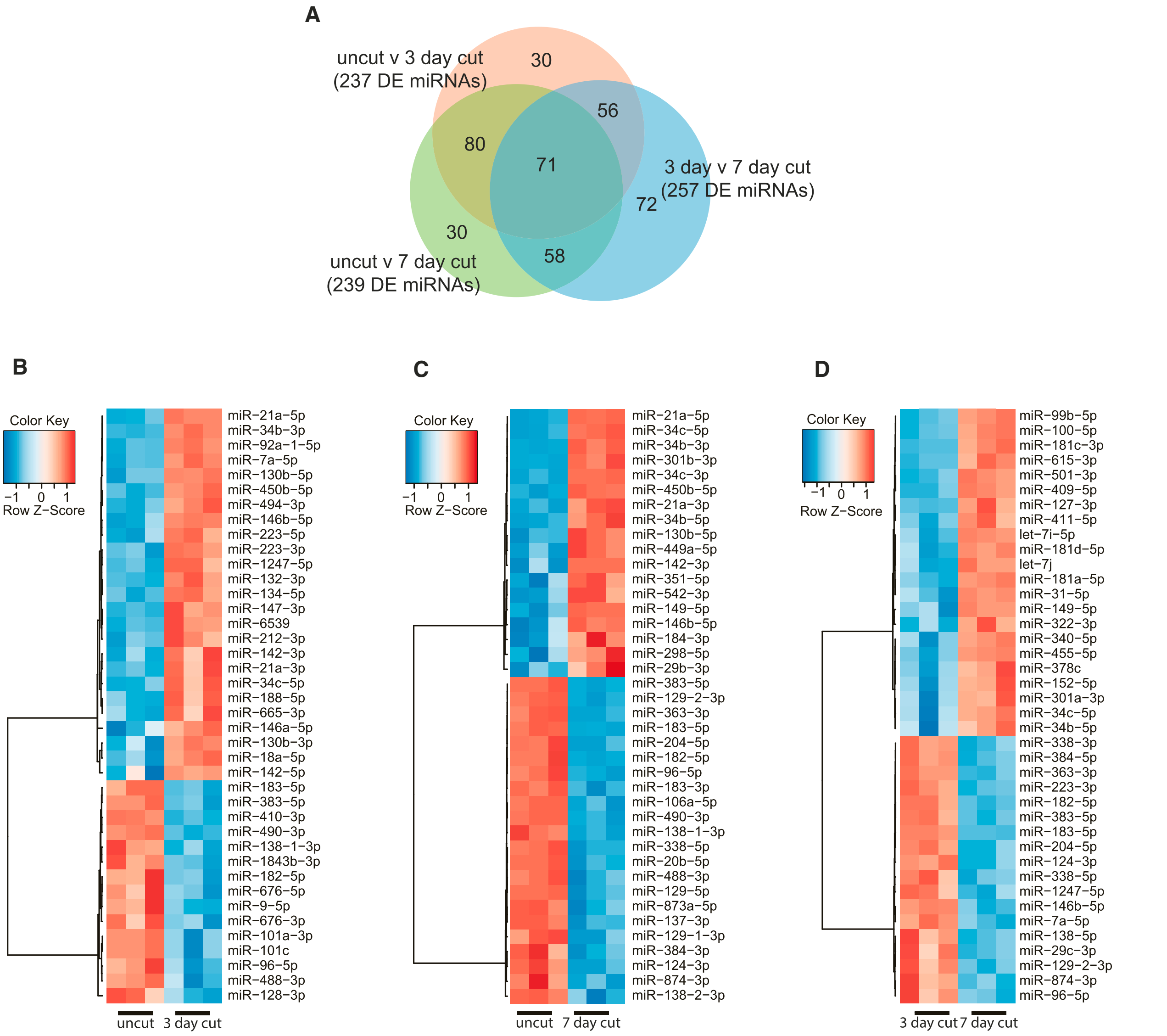

E

UPREGULATED EMT-ASSOCIATED miRNAs

miRNA Name	FC uncut v 7d cut	p-adj
miR-21a-3p	26.75	2.41E-43
miR-34c-5p	16.78	7.50E-54
miR-21a-5p	16.10	4.42E-54
miR-34c-3p	9.86	7.37E-26
miR-200b-3p	3.47	2.58E-11
miR-149-5p	3.09	7.13E-12
miR-200a-3p	2.94	0.031
miR-221-3p	2.35	1.37E-06
miR-15b-5p	2.34	1.46E-05
miR-221-5p	2.26	1.13E-07
miR-222-3p	2.09	4.51E-06

DOWNREGULATED EMT-ASSOCIATED miRNAs

miRNA Name	FC uncut v 7d cut	p-adj
miR-137-3p	-123.24	4.84E-28
miR-33-3p	-4.42	0.00025
miR-30a-3p	-2.38	2.14E-07
miR-30a-5p	-1.59	0.011

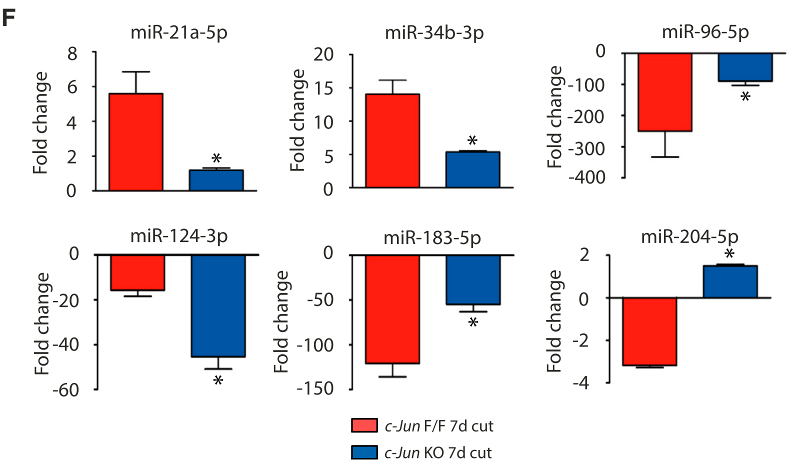

(legend on next page)

TF-binding motifs for *c-Jun* and other AP-1 members that are expressed as part of the repair Schwann cell phenotype.

Differential CpG Methylation in Enhancers of Repair Program Genes

We correlated WGSB-seq data with gene expression data from the RNA-seq experiments (Tables S1 and S5) 7 days after nerve cut. No correlation with gene expression was observed between DM CpGs found within exons, introns, intergenic regions, or enhancers, and weak negative correlation was observed with DM in the promoter region based on a limited number of CpGs (Figure S5A). Of the DM CpGs that occurred in enhancers, we found enrichment of genes associated in particular with ERBB, transforming growth factor β (TGF-β), and neurotrophin signaling (Figure 6A). Enhancers are tissue-specific regulatory elements that exhibit low levels of sequence conservation across vertebrates (Villar et al., 2015). While histone marks of enhancer activity have not been studied in injured mouse nerves, a recent study identified H3K27acylation chromatin immunoprecipitation sequencing (ChIP-seq) peaks in injured rat nerve (Hung et al., 2015), and we mapped 2,529 of these rat enhancers to mm10 mouse genome coordinates. Despite the majority of the mouse DM CpGs occurring >1,000 bp away from the mapped enhancers, 18 DM CpGs either overlapped or were in syntenic conservation with the mapped sciatic nerve enhancers, which were associated with 23 unique genes, including *Nedd4l*, *Arl4c*, *Filip1*, and *Camk2d* (Table S5).

We identified two DMRs, within intronic regions of *Nedd4l*, which contained three DM CpGs within 22 bp and five DM CpGs within 146 bp. These DMRs had a mean decrease in CpG methylation of 66.9% and 49.9%, respectively, which we also validated by Sanger bisulfite sequencing (Figures 6B, 6C, and S5B). The second DMR was located within a known mouse enhancer region and in close proximity to a mapped rat sciatic nerve enhancer (Figure 6B). Sanger bisulfite sequencing showed that these two *Nedd4l*-associated DMRs are significantly hypomethylated in cultured Schwann cells relative to macrophages and fibroblasts (Figure 6D). We also confirmed *Nedd4l* upregulation in injured sciatic nerve by qPCR and demonstrated that *Nedd4l* mRNA is more highly expressed in cultured Schwann cells than in macrophages and nerve fibroblasts (Figures 6E and 6F). In addition to *Nedd4l*, we found relative hypomethylation of a DMR in the *Cln8* gene along with strong expression of *Cln8* mRNA in cultured Schwann cells when compared to cultured macrophages and fibroblasts (Figures 6G and 6H; Tables S2A and S2B).

In summary, these findings indicate that Schwann cell-specific CpG methylation changes occur within putative enhancer regions of the genome and are correlated with changes in expression of their nearest gene, such as in the case for *Nedd4l* and *Cln8*.

DISCUSSION

The aims of the present study were to characterize more fully the genetic and epigenetic signature of the repair Schwann cell, define further how the phenotype of this cell depends on c-Jun, and determine the DNA methylation changes that accompany the conversion of myelin and Remak Schwann cells to repair Schwann cells in injured nerves.

AP-1 TF Expression in Repair Schwann Cells

C-JUN has to homo- or heterodimerize with itself or another AP-1 family member to form transcriptionally active complexes (Wagner, 2002); however, the binding partner for C-JUN in repair Schwann cells is unknown. We find that both *Fosl2* and *Atf3* show expression profiles similar to *c-Jun*, and along with *Fos*, they are the main AP-1 family members highly expressed at the mRNA level 7 days after nerve cut. Interestingly, *c-Jun*, *JunD*, *Fos*, *Fosl2*, and *Atf3* were all significantly elevated only 24 hr after nerve injury, as were other repair-program genes, such as *Nav2* and *Runx2*. At this time point, myelin genes such as *Egr2* and *Mpz* are still highly expressed and axonal degeneration has not yet taken place (Conforti et al., 2014). This suggests that at early time points after nerve injury, Schwann cells may express transcriptional components of both the repair program and the myelin program. In further support of this, phosphorylation of the ERBB2 receptor within 1 hr and the p38 mitogen-activated protein kinase (MAPK) and extracellular signal-related kinase (ERK) pathways within 6–24 hr is observed in Schwann cells after nerve injury (Guertin et al., 2005; Harrisingh et al., 2004; Yang et al., 2012). Additionally, a recent study showed that there is loss of the repressive histone mark H3K27me3 at the promoter and gene bodies of a number of repair program genes such as *Shh*, *Gdnf*, and *Fgf5* within 24 hr of nerve injury in the rat (Ma et al., 2016). Together, these findings support the view that Schwann cells may in part be able to sense nerve injury before axonal degeneration has taken place.

Repair Schwann Cell Formation Involves EMT

Repair Schwann cell formation represents an adaptive response of an adult differentiated cell type to tissue injury (Jessen et al., 2015). The transition from a myelinated Schwann cell to a repair Schwann cell shares some similarities with EMT. Using a database of all mRNAs and miRNAs currently known to have a role in EMT, we have shown that these genes are markedly enriched in our datasets from injured nerve samples from both wild-type and Schwann cell-specific *c-Jun*-knockout mice

Figure 4. miRNA Expression after Nerve Injury

(A) Venn diagram showing the overlap of DE miRNAs between uncut, 3-day cut, and 7-day cut sciatic nerve.

(B–D) Heatmap of the top 40 DE miRNAs between (B) uncut and 3-day cut nerves, (C) uncut and 7-day cut nerves, and (D) 3-day cut and 7-day cut nerves (n = 5, p-adj < 0.05).

(E) Enrichment analysis of EMT associated miRNAs from small RNA-seq study showing the most upregulated and downregulated miRNAs (FC, Fold change; p-adj < 0.05).

(F) Expression of miRNAs in *c-Jun* flox/flox (control; red) and *c-Jun* null (*P0Cre c-Jun* flox/flox; blue) 7-day cut nerves. *p < 0.05, n = 3. Fold change is relative to the uncut control nerve (*c-Jun* flox/flox). Error bars represent SEM.

OPEN
ACCESS
CellPress

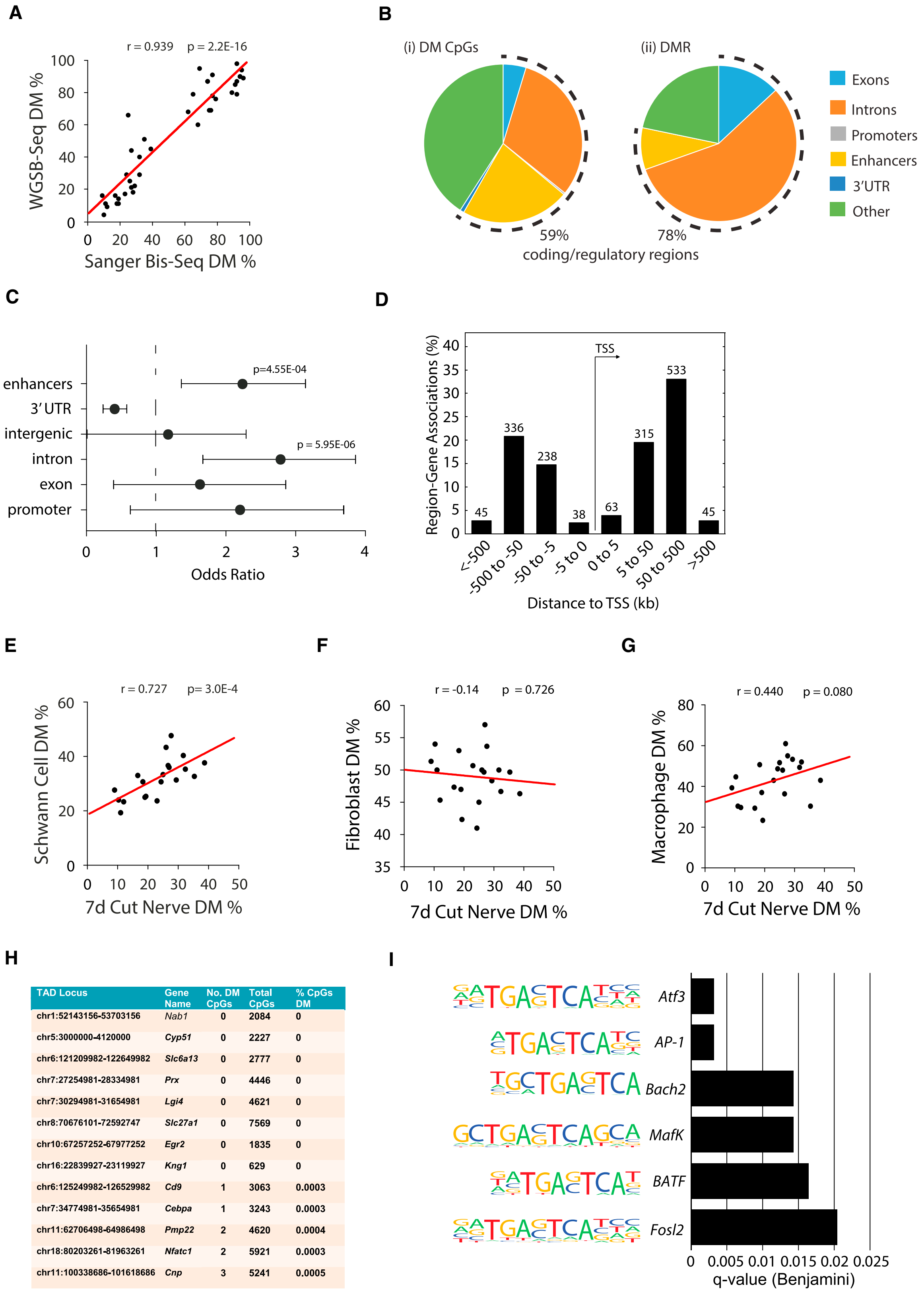

TAD Locus	Gene Name	No. DM CpGs	Total CpGs	% CpGs DM
chr1:52143156-53703156	*Nab1*	0	2084	0
chr5:3000000-4120000	*Cyp51*	0	2227	0
chr6:121209982-122649982	*Slc6a13*	0	2777	0
chr7:27254981-28334981	*Prx*	0	4446	0
chr7:30294981-31654981	*Lgi4*	0	4621	0
chr8:70676101-72592747	*Slc27a1*	0	7569	0
chr10:67257252-67977252	*Egr2*	0	1835	0
chr16:22839927-23119927	*Kng1*	0	629	0
chr6:125249982-126529982	*Cd9*	1	3063	0.0003
chr7:34774981-35654981	*Cebpa*	1	3243	0.0003
chr11:62706498-64986498	*Pmp22*	2	4620	0.0004
chr18:80203261-81963261	*Nfatc1*	2	5921	0.0003
chr11:100338686-101618686	*Cnp*	3	5241	0.0005

(legend on next page)

(Arthur-Farraj et al., 2012; Zhao et al., 2015). A number of these genes have already been shown to have roles in repair Schwann cells, including *Notch1*, *Sonic Hedgehog*, and members of the miR-34, miR-221, and miR-222 families (Martinez et al., 2015; Viader et al., 2011; Woodhoo et al., 2009; Zhou et al., 2016). Additionally, repair Schwann cells also demonstrate nuclear translocation of β-catenin, a key step in EMT, and express a number of genes expressed by mesenchymal tissue such as *α5 integrin* and *vimentin* and, at the protein level, N-cadherin and neural cell adhesion molecule (Arthur-Farraj et al., 2012; Jung et al., 2011; Lamouille et al., 2014; Table S1).

EMT has a well-established role in tissue remodeling, repair, and fibrosis (Thiery et al., 2009). Tissue injury in organs, such as the kidney and intestine, results in mesenchymal-like cell formation through EMT to promote tissue repair. This process requires a prominent inflammatory response to drive the process, which is another similarity shared with nerve injury (Thiery et al., 2009). The similarities between repair Schwann cell generation on the one hand and EMT on the other are therefore likely to reflect the relationship between this event and injury responses in other tissues. EMT is an important mechanism in tumor formation and invasiveness, and in repair Schwann cells, the involvement of this process may also relate to their capacity to form malignant nerve sheath tumors. Further investigation of the molecular mechanism that underlies the repair Schwann cell transition may therefore potentially benefit both the fields of regenerative medicine and cancer biology.

Non-coding RNA Expression in Repair Schwann Cells

The role of lncRNAs in the regulation of gene expression is incompletely understood, but they have broadly described roles in regulating transcription, translation, and chromatin remodeling (Quinn and Chang, 2016). After nerve injury, we identified 52 annotated and 17 predicted high-confidence DE lncRNAs. A recent study found 3,314 DE lncRNAs in mouse 7-day crushed nerves (Pan et al., 2017). The disparity between their findings and ours are influenced by (1) the use of a nerve crush versus nerve cut model, (2) parametric tests to determine differential expression versus utilization of the negative binomial distribution, (3) less stringent cutoffs for lowly expressed lncRNAs, and (4) not including FANTOM5 cap analysis of gene expression (CAGE) data to limit lncRNAs to those with defined 5′ ends (Hon et al., 2017).

We found that both *Sox2* overlapping transcript (*Sox2ot*) and *H19* were highly expressed in cultured Schwann cells. *Sox2ot* is a multi-exon lncRNA that contains the *Sox2* gene within one of its introns. *Sox2* is strongly expressed in repair Schwann cells and may have a role in cell-to-cell contact (Parrinello et al., 2010). Interestingly, *Sox2ot* has been shown to positively regulate the expression of *Sox2* in a breast cancer cell line (Askarian-Amiri et al., 2014), suggesting that it may be a regulator of *Sox2* expression in repair Schwann cells too. *H19* is a maternally expressed, paternally imprinted gene, which resides in close proximity to the *Igf2* gene, which is reciprocally imprinted and paternally expressed. *H19* has been shown to regulate osteogenesis, partly through modulation of *Runx2*-dependent gene expression, which is a TF that is also highly expressed in repair Schwann cells (Huang et al., 2015).

Our results have greatly expanded the list of known miRNAs regulated after nerve injury (Adilakshmi et al., 2012; Viader et al., 2011; Zhou et al., 2016). Using six independent predictor methods, we revealed the TFs *c-Jun* and *Foxd3* as potential key regulators of a number of miRNAs after nerve injury. Through analysis of a Schwann cell-specific knockout of *c-Jun*, we found six dysregulated miRNAs out of 15 tested 7 days after nerve cut. One of the most significantly regulated miRNAs, miR-21, has been shown to be a direct target of C-JUN in vascular endothelial cells (Zhou et al., 2011), and we found that miR-21-3p is expressed at much lower levels in *c-Jun*-null injured nerves than in control injured nerves. The function of miR-21 in Schwann cells is unknown, but it has been shown to target Sox2 during differentiation of hair-follicle-derived neural crest stem cells into Schwann cells (Ni et al., 2014).

The Role of CpG Methylation after Nerve Injury

Roles for cytosine methylation have been implicated in cell differentiation, particularly in the hematopoietic system, Schwann cell myelination, and other processes such as tumorigenesis, splicing, and X chromosome inactivation (Beerman et al., 2013; Jones, 2012; Varela-Rey et al., 2014). In the PNS, mutations in DNA methyltransferase 1 cause a form of hereditary sensory and autonomic neuropathy through aberrant DNA methylation (Klein et al., 2011). Furthermore, methylome analysis of neuronal cell bodies in dorsal root ganglia (DRG) shows tissue-specific changes in response to nerve injury (Gölzenleuchter et al., 2015). In Schwann cells, high-resolution methylation maps derived from reduced representation bisulfite sequencing showed global hypomethylation of promoter and enhancer regions of a large number of myelin-associated genes in neonatal mouse nerves during myelination relative to mature adult nerves. Hypomethylation of these genes was correlated with downregulation of gene expression, and artificially increasing global DNA

Figure 5. Overview of the Methylome in the Injured Nerve

(A) Validation of WGSB-seq results by Sanger sequencing of bisulfite-treated DNA. Strong correlation of total methylation percentage of 20 individual CpGs within six DMRs in WGSB-seq compared with Sanger sequencing. DMRs related to genes *Cln8*, *Mob3b*, *Arl4C*, and *Nr1h4* and two DMRs in *Nedd4l* were used.

(B) Percentage of DM CpGs (i) or DMRs(ii) in coding/regulatory regions (dotted line) or other genomic loci in 7-day cut nerve.

(C) Enrichment of DM CpGs across genomic loci with odds ratio and confidence intervals (x axis), genomic loci (y axis), and significant p-adj values shown. Error bars represent SEM.

(D) Distance of DM CpGs to the transcription start site (TSS), binned by distance and gene orientation, with total number of DM CpGs shown above each bin.

(E–G) Percentage total methylation of 20 individual CpGs within six DMRs by Sanger sequencing in 7-day cut nerves compared with cultured (E) mouse Schwann cells, (F) nerve fibroblasts, and (G) activated macrophages (n = 3, *p-adj < 0.05).

(H) Table demonstrating lack of DM around myelin genes after nerve injury. For each topological associated domain (TAD) locus containing a known myelin gene, the percentage of DM CpGs is shown.

(I) Enrichment of significant transcription-factor-binding motifs in close proximity to DM CpGs.

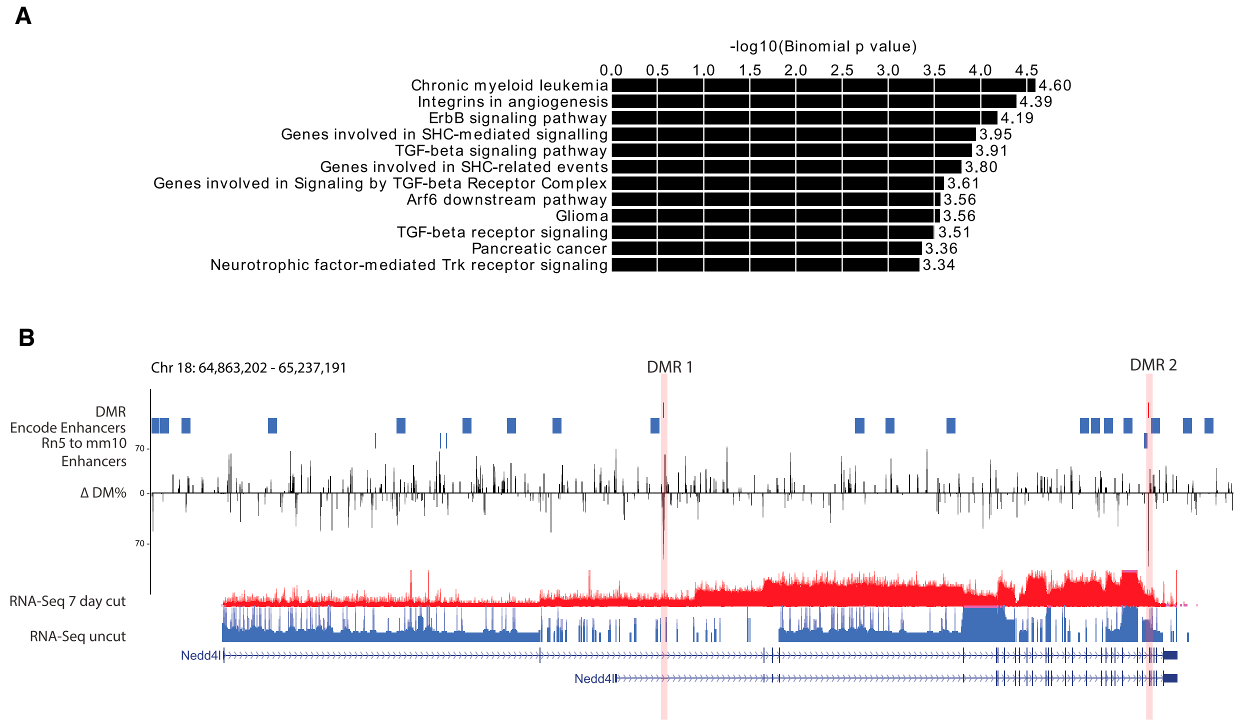

C

Genomic Location	*Nedd4l* DMR	Individual CpG Mean % DM uncut vs 7d cut WGSB-Seq	Sanger Bis-Seq
65207793	1	-62	-69
65207803	1	-71	-68
65207813	1	-69	-67
65040025	2	-32	-67
65040044	2	-50	-62
65040062	2	-50	-68
65040088	2	-63	-62
65040169	2	-58	-68

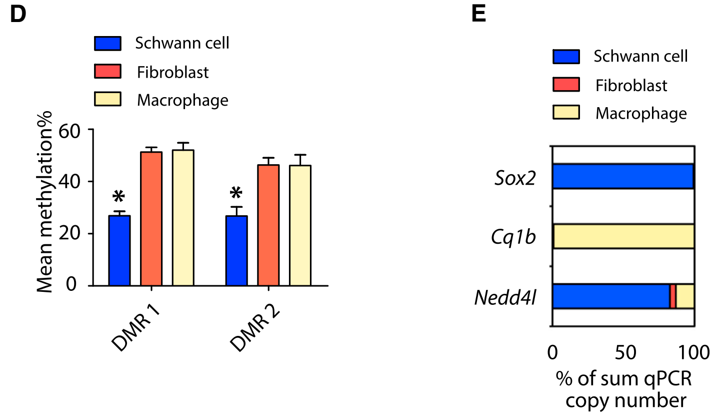

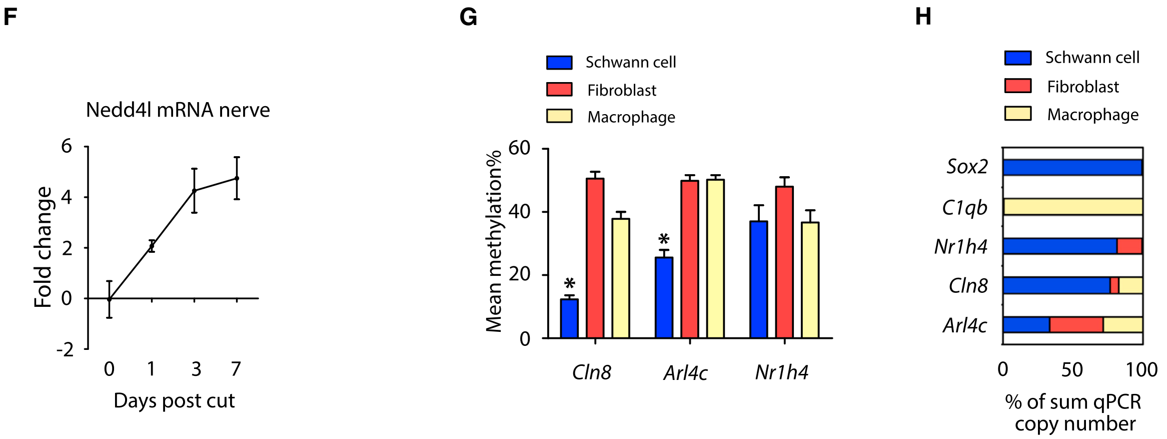

(legend on next page)

methylation in Schwann cells in vitro and in vivo perturbed myelin gene expression and myelination (Varela-Rey et al., 2014). Additionally, a recent study performed methylated DNA immunoprecipitation sequencing on cultured Schwann cells that had been harvested from the distal stump of a 7-day cut adult rat sciatic nerve. Since their control condition, in this study, was Schwann cells from an uninjured brachial plexus, which they also cultured, it is difficult to interpret how their results are readily translatable to the changes that a Schwann cell undergoes during nerve injury in vivo (Zhou et al., 2017).

In our study, methylation maps generated from repair Schwann cells in injured nerves did not identify any significant global DM in any myelin-associated genes. In fact, using methods identical to those that we used previously to show differential methylation of tens of thousands of CpGs between rat strains or during development (Johnson et al., 2012, 2014), out of over four million CpGs analyzed, we found only 853 DM CpGs. This would suggest that CpG methylation plays a less extensive role in adult cellular plasticity than it does in relation to germline DNA differences or in development. Additionally, this finding highlights that the downregulation of myelin genes that accompanies cessation of active myelination is regulated differently from that seen after injury. One possible caveat with this conclusion would be that our study under estimated the true number of DM CpGs. However, we maintained adequate average sequencing coverage of our samples, in line with current recommendations for WGSB-seq, and there was equal coverage of genomic regions of interest within individual samples (Figures S4B and S4G; Ziller et al., 2015). Furthermore, genome-wide methylation studies in other injury models, such as keloid scar formation and toxic injury to sperm, demonstrated a relatively low percentage of DM, comparable to our study (Jones et al., 2015; Dere et al., 2016).

Although a small percentage of DM CpGs were identified, the majority were located in gene regulatory areas of the genome, and a significant proportion were located within putative enhancer regions. This suggests that CpG methylation may have a biologically significant role after nerve injury. However, while a number of these potential enhancer regions were located near mapped active rat nerve injury enhancers (Hung et al., 2015), conservation of enhancer sequences is poor (Villar et al., 2015). Thus, mapping of active enhancers in mouse nerves after injury using H3K27acylation ChIP-seq will be required to show functional conservation and identify additional nerve enhancers in mouse. Enrichment analysis of DM CpGs revealed genes associated with ERBB, TGF-β, and neurotrophin signaling, which have all been previously identified in repair Schwann cells (Boerboom et al., 2017; Guertin et al., 2005; Scherer et al., 1993). This suggests that DNA methylation may have roles in modulating growth factor signaling in repair Schwann cells. When we looked in detail at one gene, *Nedd4l*, which is highly expressed in cultured Schwann cells, we found that there were two specific hypomethylated CpG clusters located within introns. One CpG cluster was located in a known mouse enhancer, which mapped very closely to an active enhancer in injured rat peripheral nerve (Hung et al., 2015). Both CpG clusters were hypomethylated in cultured mouse Schwann cells, but not in macrophages or nerve fibroblasts, suggesting that these changes are likely to be Schwann cell specific. NEDD4L is an E3 ubiquitin ligase that targets various ion channels and proteins involved in growth factor receptor signaling for proteasomal degradation, in particular components of TGF-β and nerve growth factor signaling (Goel et al., 2015). It is therefore possible that *Nedd4l* regulates signaling of multiple growth factors in repair Schwann cells, although further experiments will be needed to explore this.

While the role of CpG methylation in negatively regulating gene expression has been well documented in imprinting and X chromosome inactivation, the causal relationship between DNA methylation in other regions of the genome, including enhancer regions, with gene expression is less certain (Jones, 2012). In our study, enhancer DMRs were associated with both up- and downregulated genes. This is similar to findings for DMR in enhancer regions for Schwann cells during myelination (Varela-Rey et al., 2014). There is evidence from T cells, using luciferase reporter assays, that DMRs within lineage-specific enhancers can differentially regulate enhancer activity (Schmidl et al.,

Figure 6. Enhancer-Specific Differential Methylation in the Injured Nerve

(A) Pathway enrichment of genes associated with DM enhancer loci.

(B) Differential CpG methylation after 7-day nerve cut compared to uncut nerve along the *Nedd4l* gene. The two DMRs, both of which were significantly demethylated after nerve cut, are marked in red, and mouse (mm10) encode enhancers and active rat (rn5) nerve enhancers, identified by Hung et al., (2015), are shown. DMR2 is located in close proximity to an active rat injured nerve enhancer. Mean RNA-seq data for uncut (blue) and 7-day cut (red) nerves are shown in addition to the location of all introns (arrowed blue lines) and exons (vertical blue lines) within the *Nedd4l* gene.

(C) Genomic location and mean DM between uncut and 7-day cut nerves of individual CpGs within both DMRs of the *Nedd4l* gene measured by WGSB-seq and by bisulfite Sanger sequencing.

(D) Mean methylation percentage for the two DMRs within *Nedd4l* enhancers in cultured mouse Schwann cells (blue), nerve fibroblasts (red), and activated macrophages (yellow). *p-adj < 0.05, n = 3.

(E) Relative cell type expression of *Nedd4l* mRNA in cultured mouse Schwann cells (blue), nerve fibroblasts (red), and activated macrophages (yellow) displayed as percentage of the sum of qPCR copy-number values from 1 μg RNA from each of the three cell types (n = 3). *Sox2* and *Cq1b* are included as positive controls for Schwann cells and macrophages, respectively.

(F) Time course of *Nedd4l* mRNA expression in the distal segment after sciatic nerve cut (n = 4).

(G) Mean methylation percentage for DMRs near *Arl4c*, *Cln8*, and *Nr1h4* genes in cultured mouse Schwann cells (blue), nerve fibroblasts (red), and activated macrophages (yellow). *p-adj < 0.05.

(H) Relative cell-type expression of *Arl4c*, *Cln8*, and *Nr1h4* mRNA in cultured mouse Schwann cells (blue), nerve fibroblasts (red), and activated macrophages (yellow) displayed as percentage of the sum of qPCR copy-number values from 1 μg RNA from each of the three cell types (n = 3). *Sox2* and *Cq1b* are included as positive controls for Schwann cells and macrophages, respectively.

Error bars in all graphs represent SEM.

2009). Furthermore, genome-wide studies of the TF NRF in mouse embryonic stem cells identified that its binding to motifs in regulatory regions is influenced by local changes in CpG methylation (Domcke et al., 2015). Certainly, in our study, we found enrichment of DM CpGs within 40 bp of AP-1 TF-binding sites, in particular *Atf3* and *Fosl2* sites, suggesting that DNA methylation may have a biologically important and possibly causal relationship with TF binding, although the directionality of such a relationship has not been investigated here. Further analysis will be needed to test whether DM around TF-binding sites causally influences enhancer activity in repair Schwann cells.

A limitation of this study is that both RNA and methylation sequencing were carried out on whole nerve preparations. Nerves used for methylation analysis were desheathed beforehand, increasing Schwann cell purity to 80%. However, desheathing was not compatible with high-quality RNA extraction, with Schwann cell purity estimated between 48% and 74% in samples used for RNA-seq (Table S2C). To attempt to explore Schwann cell-specific methylation changes and RNA expression, we used purified cultures of Schwann cells, which replicate the expression of many repair Schwann cell genes (Arthur-Farraj et al., 2012). In addition, we repeated the analysis using nerve-derived fibroblasts (which would also include perineurial cells) and bone-marrow-derived macrophages treated with LPS, which constitute the two other main cell populations within the injured nerve. While we were able to show that 24 out of 33 RNAs were specifically highly expressed in Schwann cells in vitro, these findings still need to be confirmed in vivo using in situ hybridization. Furthermore, using Sanger sequencing, we confirmed similar methylation percentages of specific CpGs in cultured Schwann cells and whole nerve samples. Importantly macrophages and nerve fibroblasts showed different methylation profiles. While this finding lends weight to the likelihood that our whole-nerve sequencing results demonstrate DM within Schwann cells, single-cell analysis will reveal cell-type-specific and inter-cell heterogeneity of the DM profile.

In conclusion, this work provides a basis for understanding the molecular mechanisms underlying cell plasticity and a framework for functional studies aimed at identifying drug targets for the development of therapies for nerve injuries and peripheral neuropathies.

EXPERIMENTAL PROCEDURES

Animal experiments conformed to UK home office guidelines and were performed with institutional permission from University College London. 6- to 8-week-old male C57BL/6J mice were obtained from The Jackson Laboratory. *P0CRE*$^{+}$/*c-Jun*$^{f/f}$ male mice were generated as previously described (Parkinson et al., 2008). *c-Jun*$^{f/f}$ littermates were used as controls.

Nerve Transection and Cell Culture Experiments

All experiments used sciatic nerve cuts, which were performed at the sciatic notch as previously described (Arthur-Farraj et al., 2012). The proximal stump was diverted away from the distal stump, but not ligated. No animals showed any reconnection of axons between proximal and distal stumps. Nerves were harvested after 3 or 7 days, and the contralateral uninjured nerve was used as a control (referred to as uncut).

For cell culture methods, see Supplemental Experimental Procedures.

RNA Extraction, Total RNA-Seq, and Small RNA-Seq Library Preparation

RNA was extracted from uncut and 3-day and 7-day cut sciatic nerves and cultures of neonatal mouse Schwann, adult nerve fibroblasts, and activated macrophages using TRIzol (Invitrogen). Nerves from two mice were pooled together for each biological replicate. The integrity and quantity of RNA was determined using Qubit (Invitrogen) and Agilent 2100 Bioanalyzers (Agilent Technologies).

1 μg total RNA was used to generate RNA-seq libraries using the TruSeq Stranded total RNA sample preparation kit (Illumina) according to the manufacturer's instructions. An average of 61 million reads per sample was obtained from RNA-seq, with an average of 79.72% mapping to the GRCm38/mm10 genome. Hierarchal clustering of RNA-seq data revealed grouping of biological replicates between uncut and 7-day cut nerves (Figure S1).

Small RNA libraries were prepared using the TruSeq Small RNA sample preparation kit (Illumina) (Supplemental Experimental Procedures).

Whole-Genome Shotgun Bisulfite Sequencing

DNA was extracted from desheathed uninjured and 7-day cut sciatic nerves and cultures of neonatal mouse Schwann, adult nerve fibroblasts, and activated macrophages using the QIAamp DNA micro kit (QIAGEN). The quantity of DNA was determined using a Qubit (Invitrogen). Libraries were prepared from individual nerves to generate methylation profiles from 100-bp paired-end reads on the HiSeq 2000 platform (Illumina). The full protocol was previously described (Johnson et al., 2012). Bisulfite-treated DNA was sequenced to a high depth with an average of 181 million reads per sample. The percentage of aligned pairs mapped to the genome with a mapping success of 72.9% (Figure S3A).

Statistical Analysis

qPCR data are presented as arithmetic mean ± SEM, and statistical significance was demonstrated using an unpaired, two-tailed Student's t test with Bonferroni correction for multiple testing where necessary. For sequencing data, detailed statistical methods are described in Supplemental Experimental Procedures. The Benjamini-Hochberg procedure was used for multiple testing.

DATA AND SOFTWARE AVAILABILITY

The accession number for the sequence reads reported in this paper is ArrayExpress: E-MTAB-5633.

SUPPLEMENTAL INFORMATION

Supplemental Information includes Supplemental Experimental Procedures, five figures, and six tables and can be found with this article online at http://dx.doi.org/10.1016/j.celrep.2017.08.064.

AUTHOR CONTRIBUTIONS

Conceptualization, P.J.A.-F. and T.J.A.; Methodology, M.A., T.J.A., C.C.M., and P.J.A.-F.; Bioinformatics and Analysis, C.C.M. and A.B.; Investigation and Validation, P.J.A.-F., M.A., B.R., J.A.G.-S., and S.V.F.; Writing – Original Draft, P.J.A.-F. and C.C.M.; Writing – Review & Editing; P.J.A.-F., C.C.M., T.J.A., K.R.J., R.M., and M.A.; Funding Acquisition, T.J.A., K.R.J., and R.M.; Resources, T.J.A., K.R.J., and R.M.; Project Oversight, P.J.A.-F., T.J.A., and M.A.

ACKNOWLEDGMENTS

We thank the CSC Genomics Core Laboratory and Imperial College High Performance Computing Service. We also thank M.L. Feltri and L. Wrabetz for the gift of P_0-*CRE* mice and A. Behrens for *Jun* flox mice. We thank S.J. Hunt, Z. D'Souza for technical assistance. This study was supported by the National Institute of Health Research (Academic Clinical Fellowship to P.J.A.-F.; 2137), the Leducq Foundation (Transatlantic Network of Excellence grant

11CVD01 to for M.A.), the British Heart Foundation (Imperial College Centre funds to C.C.M.; G0901467), and the MRC CSC (intramural funds). J.G.S. and S.V.F. were funded by the Wellcome Trust (programme grant 091119 to K.R.J. and R.M.), and B.R. was funded by the Medical Research Council.

Received: April 2, 2017
Revised: June 17, 2017
Accepted: August 18, 2017
Published: September 12, 2017

REFERENCES

Adilakshmi, T., Sudol, I., and Tapinos, N. (2012). Combinatorial action of miRNAs regulates transcriptional and post-transcriptional gene silencing following in vivo PNS injury. PLoS ONE *7*, e39674.

Arthur-Farraj, P.J., Latouche, M., Wilton, D.K., Quintes, S., Chabrol, E., Banerjee, A., Woodhoo, A., Jenkins, B., Rahman, M., Turmaine, M., et al. (2012). c-Jun reprograms Schwann cells of injured nerves to generate a repair cell essential for regeneration. Neuron *75*, 633–647.

Askarian-Amiri, M.E., Seyfoddin, V., Smart, C.E., Wang, J., Kim, J.E., Hansji, H., Baguley, B.C., Finlay, G.J., and Leung, E.Y. (2014). Emerging role of long non-coding RNA SOX2OT in SOX2 regulation in breast cancer. PLoS ONE *9*, e102140.

Beerman, I., Bock, C., Garrison, B.S., Smith, Z.D., Gu, H., Meissner, A., and Rossi, D.J. (2013). Proliferation-dependent alterations of the DNA methylation landscape underlie hematopoietic stem cell aging. Cell Stem Cell *12*, 413–425.

Boerboom, A., Dion, V., Chariot, A., and Franzen, R. (2017). Molecular mechanisms involved in Schwann cell plasticity. Front. Mol. Neurosci. *10*, 38.

Bonasio, R., Tu, S., and Reinberg, D. (2010). Molecular signals of epigenetic states. Science *330*, 612–616.

Bunge, R.P., and Bunge, M.B. (1983). Interrelationship between Schwann cell function and extracellular matrix production. Trends Neurosci. *6*, 499–505.

Chan, J.R., Jolicoeur, C., Yamauchi, J., Elliott, J., Fawcett, J.P., Ng, B.K., and Cayouette, M. (2006). The polarity protein Par-3 directly interacts with p75NTR to regulate myelination. Science *314*, 832–836.

Conforti, L., Gilley, J., and Coleman, M.P. (2014). Wallerian degeneration: an emerging axon death pathway linking injury and disease. Nat. Rev. Neurosci. *15*, 394–409.

Crawford, A.T., Desai, D., Gokina, P., Basak, S., and Kim, H.A. (2008). E-cadherin expression in postnatal Schwann cells is regulated by the cAMP-dependent protein kinase a pathway. Glia *56*, 1637–1647.

Dere, E., Wilson, S.K., Anderson, L.M., and Boekelheide, K. (2016). From the cover: sperm molecular biomarkers are sensitive indicators of testicular injury following subchronic model toxicant exposure. Toxicol. Sci. *153*, 327–340.

Domcke, S., Bardet, A.F., Adrian Ginno, P., Hartl, D., Burger, L., and Schübeler, D. (2015). Competition between DNA methylation and transcription factors determines binding of NRF1. Nature *528*, 575–579.

Fontana, X., Hristova, M., Da Costa, C., Patodia, S., Thei, L., Makwana, M., Spencer-Dene, B., Latouche, M., Mirsky, R., Jessen, K.R., et al. (2012). c-Jun in Schwann cells promotes axonal regeneration and motoneuron survival via paracrine signaling. J. Cell Biol. *198*, 127–141.

Goel, P., Manning, J.A., and Kumar, S. (2015). NEDD4-2 (NEDD4L): the ubiquitin ligase for multiple membrane proteins. Gene *557*, 1–10.

Gökbuget, D., Pereira, J.A., Bachofner, S., Marchais, A., Ciaudo, C., Stoffel, M., Schulte, J.H., and Suter, U. (2015). The Lin28/let-7 axis is critical for myelination in the peripheral nervous system. Nat. Commun. *6*, 8584.

Gölzenleuchter, M., Kanwar, R., Zaibak, M., Al Saiegh, F., Hartung, T., Klukas, J., Smalley, R.L., Cunningham, J.M., Figueroa, M.E., Schroth, G.P., et al. (2015). Plasticity of DNA methylation in a nerve injury model of pain. Epigenetics *10*, 200–212.

Gomez-Sanchez, J.A., Carty, L., Iruarrizaga-Lejarreta, M., Palomo-Irigoyen, M., Varela-Rey, M., Griffith, M., Hantke, J., Macias-Camara, N., Azkargorta, M., Aurrekoetxea, I., et al. (2015). Schwann cell autophagy, myelinophagy, initiates myelin clearance from injured nerves. J. Cell Biol. *210*, 153–168.

Guertin, A.D., Zhang, D.P., Mak, K.S., Alberta, J.A., and Kim, H.A. (2005). Microanatomy of axon/glial signaling during Wallerian degeneration. J. Neurosci. *25*, 3478–3487.

Harrisingh, M.C., Perez-Nadales, E., Parkinson, D.B., Malcolm, D.S., Mudge, A.W., and Lloyd, A.C. (2004). The Ras/Raf/ERK signalling pathway drives Schwann cell dedifferentiation. EMBO J. *23*, 3061–3071.

Hon, C.-C., Ramilowski, J.A., Harshbarger, J., Bertin, N., Rackham, O.J.L., Gough, J., Denisenko, E., Schmeier, S., Poulsen, T.M., Severin, J., et al. (2017). An atlas of human long non-coding RNAs with accurate 5′ ends. Nature *543*, 199–204.

Huang, Y., Zheng, Y., Jia, L., and Li, W. (2015). Long noncoding RNA H19 promotes osteoblast differentiation via TGF-B1/Smad3/HDAC signaling pathway by deriving mir-675. Stem Cells *33*, 3481–3492.

Hung, H.A., Sun, G., Keles, S., and Svaren, J. (2015). Dynamic regulation of Schwann cell enhancers after peripheral nerve injury. J. Biol. Chem. *290*, 6937–6950.

Jessen, K.R., and Mirsky, R. (2016). The repair Schwann cell and its function in regenerating nerves. J. Physiol. *594*, 3521–3531.

Jessen, K.R., Mirsky, R., and Arthur-Farraj, P. (2015). The role of cell plasticity in tissue repair: adaptive cellular reprogramming. Dev. Cell *34*, 613–620.

Johnson, M.D., Mueller, M., Game, L., and Aitman, T.J. (2012). Single nucleotide analysis of cytosine methylation by whole-genome shotgun bisulfite sequencing. Curr. Protoc. Mol. Biol. *Chapter 21*, Unit 21.23.

Johnson, M.D., Mueller, M., Adamowicz-Brice, M., Collins, M.J., Gellert, P., Maratou, K., Srivastava, P.K., Rotival, M., Butt, S., Game, L., et al. (2014). Genetic analysis of the cardiac methylome at single nucleotide resolution in a model of human cardiovascular disease. PLoS Genet. *10*, e1004813.

Jones, P.A. (2012). Functions of DNA methylation: islands, start sites, gene bodies and beyond. Nat. Rev. Genet. *13*, 484–492.

Jones, L.R., Young, W., Divine, G., Datta, I., Chen, K.M., Ozog, D., and Worsham, M.J. (2015). Genome-wide scan for methylation profiles in keloids. Dis. Markers *2015*, 943176.

Jung, J., Cai, W., Lee, H.K., Pellegatta, M., Shin, Y.K., Jang, S.Y., Suh, D.J., Wrabetz, L., Feltri, M.L., and Park, H.T. (2011). Actin polymerization is essential for myelin sheath fragmentation during Wallerian degeneration. J. Neurosci. *31*, 2009–2015.

Klein, C.J., Botuyan, M.-V., Wu, Y., Ward, C.J., Nicholson, G.A., Hammans, S., Hojo, K., Yamanishi, H., Karpf, A.R., Wallace, D.C., et al. (2011). Mutations in DNMT1 cause hereditary sensory neuropathy with dementia and hearing loss. Nat. Genet. *43*, 595–600.

Lamouille, S., Xu, J., and Derynck, R. (2014). Molecular mechanisms of epithelial-mesenchymal transition. Nat. Rev. Mol. Cell Biol. *15*, 178–196.

Ma, K.H., Hung, H.A., and Svaren, J. (2016). Epigenomic regulation of Schwann cell reprogramming in peripheral nerve injury. J. Neurosci. *36*, 9135–9147.

Martinez, J.A., Kobayashi, M., Krishnan, A., Webber, C., Christie, K., Guo, G., Singh, V., and Zochodne, D.W. (2015). Intrinsic facilitation of adult peripheral nerve regeneration by the Sonic hedgehog morphogen. Exp. Neurol. *271*, 493–505.

Martini, R., Schachner, M., and Faissner, A. (1990). Enhanced expression of the extracellular matrix molecule J1/tenascin in the regenerating adult mouse sciatic nerve. J. Neurocytol. *19*, 601–616.

Ni, Y., Zhang, K., Liu, X., Yang, T., Wang, B., Fu, L., A, L., and Zhou, Y. (2014). miR-21 promotes the differentiation of hair follicle-derived neural crest stem cells into Schwann cells. Neural Regen. Res. *9*, 828–836.

Painter, M.W., Brosius Lutz, A., Cheng, Y.-C., Latremoliere, A., Duong, K., Miller, C.M., Posada, S., Cobos, E.J., Zhang, A.X., Wagers, A.J., et al. (2014). Diminished Schwann cell repair responses underlie age-associated impaired axonal regeneration. Neuron *83*, 331–343.

Pan, B., Zhou, H.-X., Liu, Y., Yan, J.-Y., Wang, Y., Yao, X., Deng, Y.-Q., Chen, S.-Y., Lu, L., Wei, Z.-J., et al. (2017). Time-dependent differential expression of long non-coding RNAs following peripheral nerve injury. Int. J. Mol. Med. *39*, 1381–1392.

Parkinson, D.B., Bhaskaran, A., Arthur-Farraj, P., Noon, L.A., Woodhoo, A., Lloyd, A.C., Feltri, M.L., Wrabetz, L., Behrens, A., Mirsky, R., and Jessen, K.R. (2008). c-Jun is a negative regulator of myelination. J. Cell Biol. *181*, 625–637.

Parrinello, S., Napoli, I., Ribeiro, S., Wingfield Digby, P., Fedorova, M., Parkinson, D.B., Doddrell, R.D.S., Nakayama, M., Adams, R.H., and Lloyd, A.C. (2010). EphB signaling directs peripheral nerve regeneration through Sox2-dependent Schwann cell sorting. Cell *143*, 145–155.

Quinn, J.J., and Chang, H.Y. (2016). Unique features of long non-coding RNA biogenesis and function. Nat. Rev. Genet. *17*, 47–62.

Sabin, L.R., Delás, M.J., and Hannon, G.J. (2013). Dogma derailed: the many influences of RNA on the genome. Mol. Cell *49*, 783–794.

Scherer, S.S., Kamholz, J., and Jakowlew, S.B. (1993). Axons modulate the expression of transforming growth factor-betas in Schwann cells. Glia *8*, 265–276.

Schmidl, C., Klug, M., Boeld, T.J., Andreesen, R., Hoffmann, P., Edinger, M., and Rehli, M. (2009). Lineage-specific DNA methylation in T cells correlates with histone methylation and enhancer activity. Genome Res. *19*, 1165–1174.

Thiery, J.P., Acloque, H., Huang, R.Y.J., and Nieto, M.A. (2009). Epithelial-mesenchymal transitions in development and disease. Cell *139*, 871–890.

Varela-Rey, M., Iruarrizaga-Lejarreta, M., Lozano, J.J., Aransay, A.M., Fernandez, A.F., Lavin, J.L., Mósen-Ansorena, D., Berdasco, M., Turmaine, M., Luka, Z., et al. (2014). S-adenosylmethionine levels regulate the schwann cell DNA methylome. Neuron *81*, 1024–1039.

Viader, A., Chang, L.W., Fahrner, T., Nagarajan, R., and Milbrandt, J. (2011). MicroRNAs modulate Schwann cell response to nerve injury by reinforcing transcriptional silencing of dedifferentiation-related genes. J. Neurosci. *31*, 17358–17369.

Villar, D., Berthelot, C., Aldridge, S., Rayner, T.F., Lukk, M., Pignatelli, M., Park, T.J., Deaville, R., Erichsen, J.T., Jasinska, A.J., et al. (2015). Enhancer evolution across 20 mammalian species. Cell *160*, 554–566.

Wagner, E.F. (2002). Functions of AP1(Fos/Jun) in bone development. Ann. Rheum. Dis. *61* (*Suppl 2*), 40–42.

Woodhoo, A., Alonso, M.B.D., Droggiti, A., Turmaine, M., D'Antonio, M., Parkinson, D.B., Wilton, D.K., Al-Shawi, R., Simons, P., Shen, J., et al. (2009). Notch controls embryonic Schwann cell differentiation, postnatal myelination and adult plasticity. Nat. Neurosci. *12*, 839–847.

Yang, D.P., Kim, J., Syed, N., Tung, Y.J., Bhaskaran, A., Mindos, T., Mirsky, R., Jessen, K.R., Maurel, P., Parkinson, D.B., and Kim, H.A. (2012). p38 MAPK activation promotes denervated Schwann cell phenotype and functions as a negative regulator of Schwann cell differentiation and myelination. J. Neurosci. *32*, 7158–7168.

Zhao, M., Kong, L., Yining, L., and Hong, Q. (2015). dbEMT: an epithelial-mesenchymal transition associated gene resource. Sci. Rep. *5*, 11459.

Zhou, J., Wang, K.C., Wu, W., Subramaniam, S., Shyy, J.Y., Chiu, J.J., Li, J.Y., and Chien, S. (2011). MicroRNA-21 targets peroxisome proliferators-activated receptor-alpha in an autoregulatory loop to modulate flow-induced endothelial inflammation. Proc. Natl. Acad. Sci. USA *108*, 10355–10360.

Zhou, S., Ding, F., and Gu, X. (2016). Non-coding RNAs as emerging regulators of neural injury responses and regeneration. Neurosci. Bull. *32*, 253–264.

Zhou, X.-H., Lin, W., Ren, Y.-M., Liu, S., Fan, B.-Y., Wei, Z.-J., Shi, G.-D., Cheng, X., Hao, Y., and Feng, S.-Q. (2017). Comparison of DNA methylation in Schwann cells before and after peripheral nerve injury in rats. BioMed Res. Int. *2017*, 5393268.

Ziller, M.J., Hansen, K.D., Meissner, A., and Aryee, M.J. (2015). Coverage recommendations for methylation analysis by whole-genome bisulfite sequencing. Nat. Methods *12*, 230–232, 1, 232.

Cell Reports, Volume 20

Supplemental Information

Changes in the Coding and Non-coding Transcriptome and DNA Methylome that Define the Schwann Cell Repair Phenotype after Nerve Injury

Peter J. Arthur-Farraj, Claire C. Morgan, Martyna Adamowicz, Jose A. Gomez-Sanchez, Shaline V. Fazal, Anthony Beucher, Bonnie Razzaghi, Rhona Mirsky, Kristjan R. Jessen, and Timothy J. Aitman

SUPPLEMENTAL INFORMATION

1. Supplemental Figures and Tables

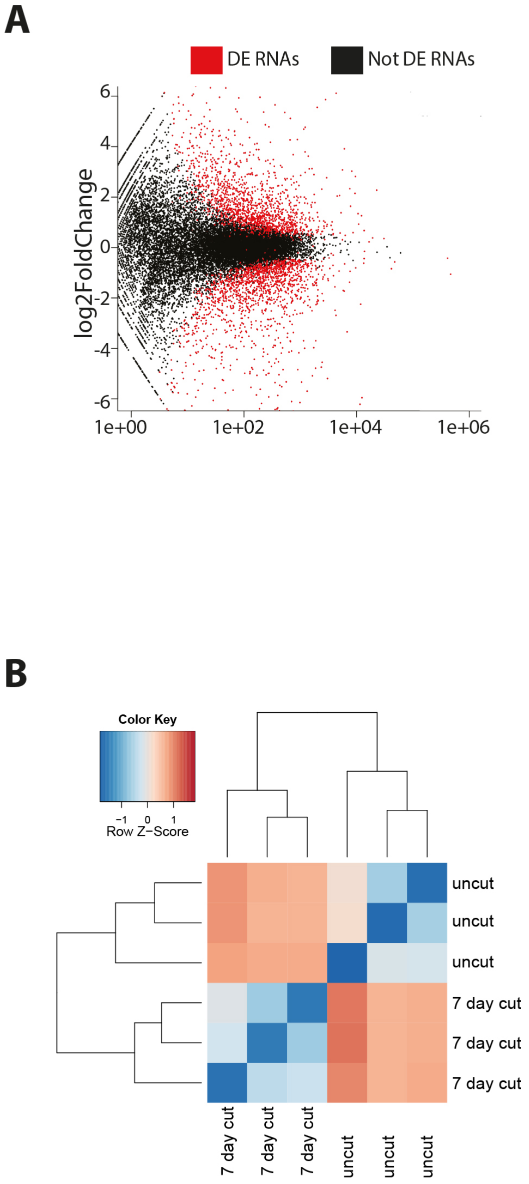

Supplemental Figure 1. RNA-Seq clustering of samples. Related to Figure 1, 2 and 3. (A) MA plot showing differentially expressed (red dots, DE genes) and non-differentially expressed (black dots, not DE genes) genes in uncut versus seven day cut nerves. (B) Clustering of RNA-Seq biological replicates for uncut and seven day cut nerves.

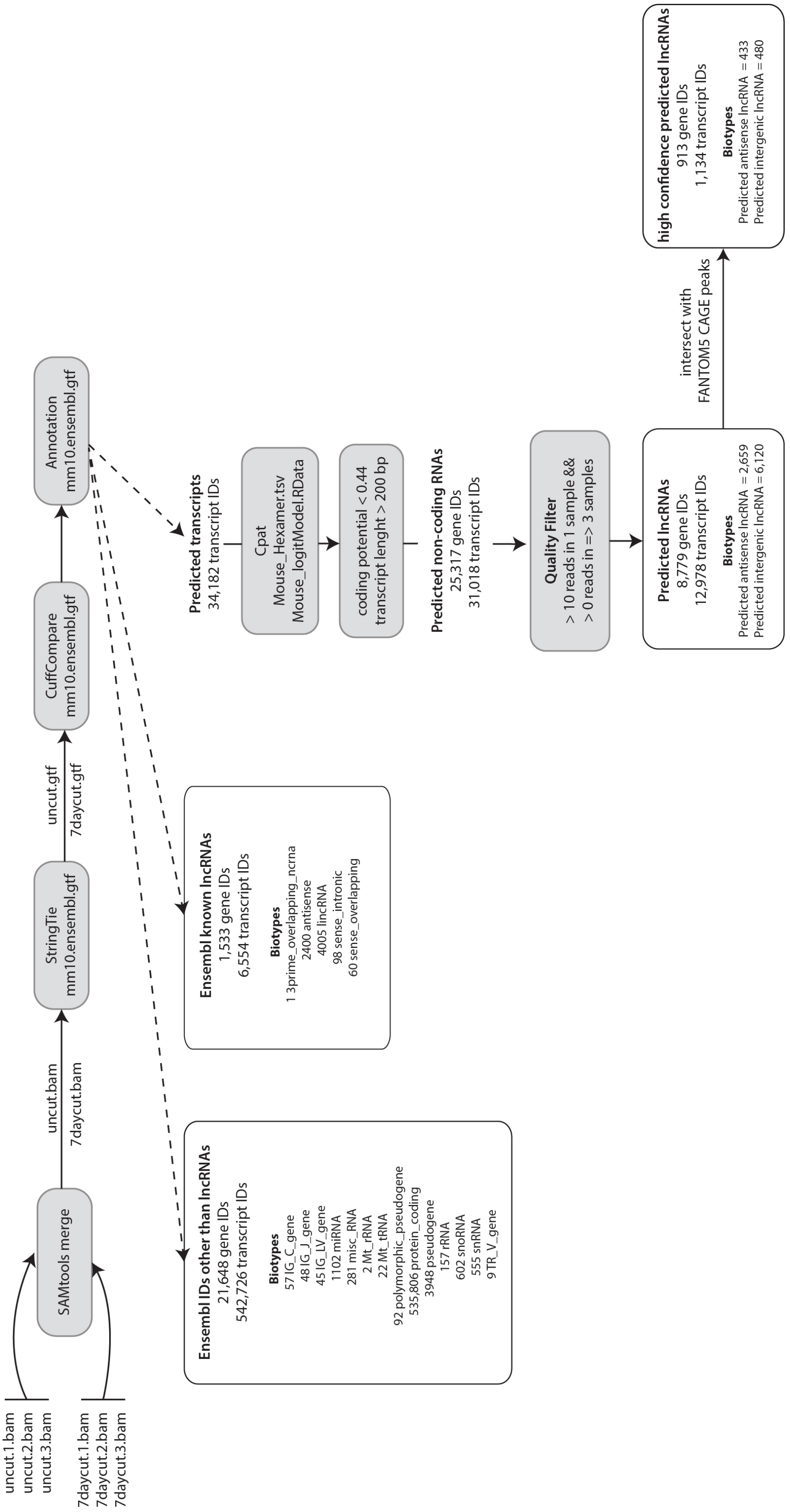

Supplemental Figure 2. Overview of the bioinformatic pipeline to isolate predicted high confidence Lnc RNA. Related to Figure 3. Predicted transcripts from RNA-Seq data, without an ENSEMBL ID, that had low protein coding potential and a minimum length of 200bp were intersected with FANTOM5 CAGE data to identify a set of high confidence lncRNAs (see Supplemental methods).

A

GENE	miRNAseq 3d cut FC	P value	qPCR 3d cut FC	P value
miR 17-5p	2.87	6.29E-06	1.60	0.016
miR 21a-3p	38.33	2.41E-43	18.98	3.50E-06
miR 34b-5p	5.28	3.73E-38	4.02	0.015
miR 34c-5p	6.51	7.50E-54	6.33	0.014
miR 132-3p	2.60	1.02E-07	3.47	0.0088
miR 142-3p	9.09	8.99E-18	6.78	0.00057
miR 362-5p	2.09	8.13E-07	1.94	0.0083
miR 92b-3p	-3.53	2.24E-09	-2.77	0.0085
miR 96-5p	-5.39	1.70E-47	-8.39	0.00027
miR 383-5p	-6.35	2.28E-78	-7.59	0.0017
miR 598-3p	-3.85	0.00013	-4.25	0.0069
miR 34a-5p	-1.04	0.96 NS	-2.31	0.016
miR 204-5p	-1.71	5.70E-23	-5.56	0.0011
miR 183-5p	-4.83	1.95E-46	-6.60	4.32E-05
miR 124-3p	-2.58	4.59E-15	-6.06	0.00017

B

GENE	miRNAseq 7d cut FC	P value	qPCR 7d cut FC	P value
miR 17-5p	1.91	0.0010	1.53	0.027
miR 21a-3p	26.75	2.41351E-43	10.97	3.90E-06
miR 34b-5p	18.15	1.90899E-23	10.10	0.0012
miR 34c-5p	9.86	7.37144E-26	19.14	0.014
miR 132-3p	2.60	1.01824E-07	1.56	0.024
miR 142-3p	4.89	8.99148E-18	5.52	0.0026
miR 362-5p	2.31	8.13282E-07	1.66	0.0072
miR 92b-3p	-5.01	2.23666E-09	-6.17	1.68E-05
miR 96-5p	-77.52	1.69617E-47	-144.02	2.54E-07
miR 383-5p	-155.79	2.27588E-78	-20.93	0.0010
miR 598-3p	-3.83	0.000127806	-4.86	0.0048
miR 34a-5p	-1.08	0.76 NS	-1.55	0.033
miR 204-5p	-4.97	5.70446E-23	-5.59	0.0012
miR 183-5p	-126.1	1.95E-46	-384.40	0.00012
miR 124-3p	-283.90	4.59193E-15	-33.56	9.67E-06

C

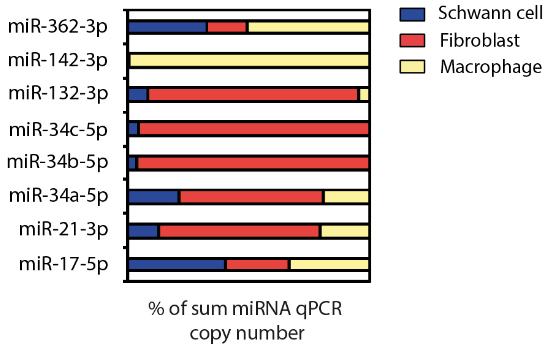

D

Enriched KEGG Pathway	miRNAs targeting enriched gene set (p-adj<0.01)
Focal adhesion	miR-29a, miR-29c, miR-409-3p, miR-485, miR-494, miR-770-5p
ECM-receptor interaction	miR-770
Leukocyte transendothelial migration	NS
**Enriched Gene ontology**	**miRNAs targeting enriched gene set (p-adj<0.001)**
regulation of cell morphogenesis	NA
fatty acid metabolic process	miR-15a, miR-15b, miR-32, miR-322, miR-378, miR-665, miR-758
regulation of signal transduction	miR-214
sterol metabolic process	miR-32
intracellular signaling cascade	miR-32, miR-199a-3p, miR-455, miR-873
regulation of small GTPase mediated signal transduction	NS
endocytosis	miR-32
**Enriched protein family**	**miRNAs targeting enriched gene set (p-adj<0.001)**
Pleckstrin homology	NS
Collagen triple helix repeat	miR-29c, miR-299, miR-29a, miR-29b, miR-455, miR-770-5p
Fibronectin type 3	NS
Plexin repeat	miR-431, miR-671-5p, miR-139-5p, miR-484, miR-874
Immunoglobulin	NS
Myosin_head	NS

E

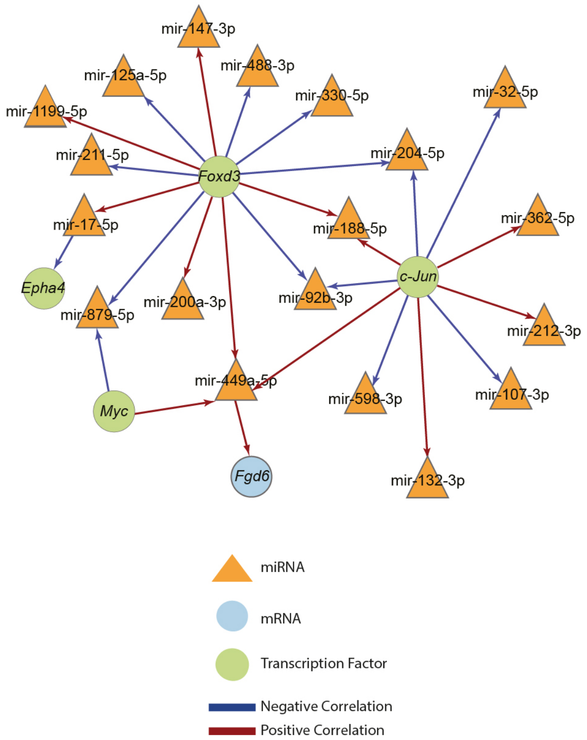

F

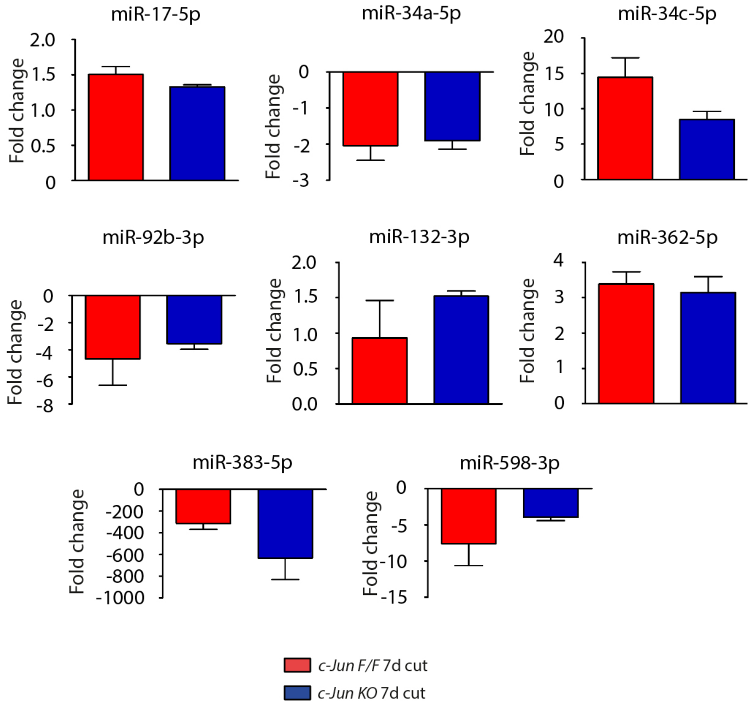

Supplemental Figure 3. miRNA profiles in the injured nerve. Related to Figure 4. (A and B) Fold change (FC) of miRNA expression values from RNA-Seq and qPCR in (A) three day cut and (B) seven day cut sciatic nerve samples compared to uncut nerves, along with associated p-adjusted values (n=5, p-adj<0.05). (C) Relative cell type expression of miRNAs in cultured mouse Schwann cells (blue), nerve fibroblasts (red) and activated macrophages (yellow) displayed as percentage of the sum of qPCR 2DCT (copy number) values from 1ug of RNA from each of the three cell types (n=3) (D) Enriched KEGG pathways, biological processes and protein families of miRNA targets, p-adj<0.05. (E) Magia[2] analysis showing putative regulatory network of miRNAs, transcription factors and genes targets following nerve cut. (F) Expression of miRNAs in *c-Jun* flox/flox (Control; red) and *c-Jun* null (P0Cre *c-Jun* flox/flox; blue) seven day cut nerves. Fold change is relative to the uncut control nerve (*c-Jun* flox/flox) (n=3, * p<0.05).

A

Mapping	aligned pairs	unique pairs	non-unique pairs
uncut 1	89608433 (92.8%)	74566713 (77.2%)	15041720 (15.6%)
uncut 2	88833543 (93.0%)	74643523 (78.2%)	14190020 (14.9%)
uncut 3	67984256 (92.4%)	56056794 (76.2%)	11927462 (16.2%)
Cut 7 days 1	90835398 (93.0%)	76880312 (78.7%)	13955086 (14.3%
Cut 7 days 2	84565914 (93.3%)	71835201 (79.3%)	12730713 (14.0%
Cut 7 days 3	78262623 (92.8%)	65711793 (77.9%)	12550830 (14.9%)
Average	83348361 (92.9%)	69949056 (72.9%)	13399305 (15.0%)

B

Coverage	Min	1st Qu.	Median	Mean	3. Qu	Max
uncut	5	6	8	10.44	11	65540
Cut 7 days	5	6	7	9.98	10	65540

C

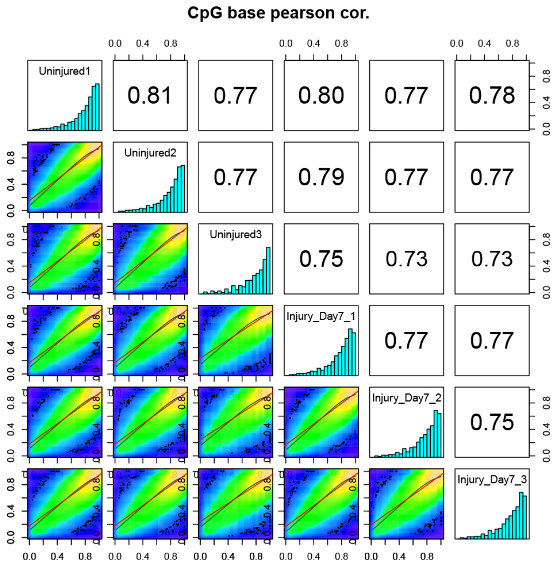

D

Annotation of individual DM CpGs	DM CpGs	promoter	exon	intron	3'UTR	intergenic	Enhancers	CpG Islands	CpG Shores	CpG Shelves
uncut v Cut 7days	853	41 (5%)	40 (5%)	285 (33%)	19 (2%)	468 (55%)	298 (34.9%)	3 (0.9%)	27 (3.2%)	37 (4.3%)

E

Annotation of 46 Clusters	DM CpGs in Clusters	promoter	exon	intron	3'UTR	intergenic	Enhancers	CpG Islands	CpG Shores	CpG Shelves
uncut v Cut 7days	114	8 (7.0%)	6 (5.3%)	60 (52.6%)	1 (0.9%)	39 (34.2%)	57 (50%)	0	0	10 (8.8%)

F

Enrichment of clustered DM CpGs	single CpGs	CpGs in Clusters	p-value	OR	95% CI	Enriched
promoter	41	8	2.39E-01	1.61	0.63, 3.69	No
exon	40	6	8.11E-01	1.15	0.39, 2.86	No
intron	285	60	5.95E-06	2.54	1.67, 3.86	Yes
3' UTR	19	1	4.96E-01	0.35	0.01, 2.29	No
intergenic	468	39	2.55E-06	0.38	0.24, 0.58	No
Enhancers	298	57	4.55E-04	2.06	1.36, 3.14	Yes

G

Uncut_1	min	1st Qu.	Median	3rd. Qu	Max
Exon	1	7	12	17	2744
Promoter	1	6	11	16	1252
3-UTR	1	6	10	15	2744
Enhancer	1	6	10	15	2000600
Intron	1	5	10	15	30913
Other	1	5	9	15	313924
**Uncut_2**	**min**	**1st Qu.**	**Median**	**3rd. Qu**	**Max**
Exon	1	7	11	16	2495
Promoter	1	6	10	15	1054
3-UTR	1	5	9	14	2495
Enhancer	1	6	10	14	1794100
Intron	1	5	9	14	28334
Other	1	4	8	14	255723
**Uncut_3**	**min**	**1st Qu.**	**Median**	**3rd. Qu**	**Max**
Exon	1	7	12	16	2597
Promoter	1	6	11	16	1090
3-UTR	1	6	10	15	2597
Enhancer	1	6	10	15	1770040
Intron	1	5	9	14	28274
Other	1	5	9	14	230895

7daycut_1	min	1st Qu.	Median	3rd. Qu	Max
Exon	1	6	9	13	1744
Promoter	1	5	8	12	822
3-UTR	1	5	8	12	1744
Enhancer	1	5	8	12	1421520
Intron	1	5	8	11	36553
Other	1	4	7	11	290759
**7daycut_2**	**min**	**1st Qu.**	**Median**	**3rd. Qu**	**Max**
Exon	1	5	9	13	1691
Promoter	1	5	8	12	1011
3-UTR	1	4	8	11	1691
Enhancer	1	4	8	12	1702930
Intron	1	4	7	12	22916
Other	1	4	7	12	318952
**7daycut_3**	**min**	**1st Qu.**	**Median**	**3rd. Qu**	**Max**
Exon	1	6	9	13	1973
Promoter	1	5	8	12	793
3-UTR	1	5	8	12	1973
Enhancer	1	5	8	12	1574250
Intron	1	4	8	12	32285
Other	1	4	7	11	307265

Supplemental Figure 4. Overview of Whole genome shotgun bisulfite data quality and results. Related to Figure 5 and 6. (A) Mapping results for aligned unique and non-unique pairs across uncut and seven day cut replicates. (B) Average depth of coverage across CpGs for uncut and seven day cut samples. (C) Pearson correlation of CpG methylation states between uncut and cut samples and technical replicates. (D and E) The genomic annotation of (D) individual DM CpGs and (E) clustered CpGs is provided. (F) Fishers exact test of clustered DM CpGs versus single DM CpGs occurrence in various genomic loci. Odd's ratio (OR) and 95% confidence interval (CI) are displayed. (G) Read coverage across different genomic regions for each individual sample sequenced.

A

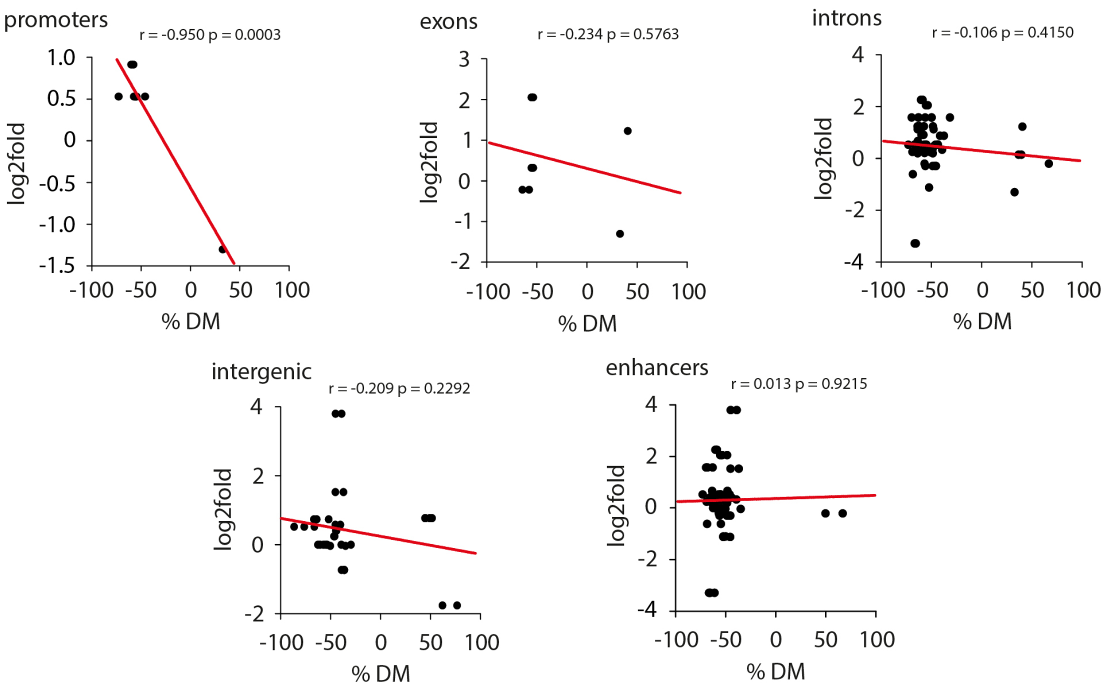

B

Cluster Location	# DM CpGs	Δ %	Closest Gene	Annotation	FC	p-adj	r
chr8:125498650-125498713	3	-37.1	*Sipa1l2*	6055 up	-1.66	2.01E-04	+ve
chr11:51881210-51881377	3	-60.8	*Jade2*	24085 up	1.67	4.11E-02	-ve
chr12:100878642-100878656	3	-52.5	*9030617O03Rik*	Exon	1.25	1.00E+00	NA
chr18:65207791-65207813	3	-66.9	*Nedd4l*	Intron	2.99	1.49E-11	-ve
chr1:88587585-88587674	3	-59.9	*Glrp1*	77700 up	Inf	1.00E+00	NA
chr4:35096498-35096508	3	-64.4	*Mob3b*	Intron	-9.73	6.29E-31	+ve
chr6:31080847-31080985	3	-76.2	*Gm13834*	6927 down	1.44	6.83E-01	NA
chr17:84333040-84333192	4	-49.1	*Thada*	Intron	-1.22	8.33E-01	NA
chr8:14891377-14891676	4	-57.8	*Cln8*	Intron	2.36	1.79E-05	-ve
chr16:16988992-16989128	4	-58.3	*Mapk1*	Intron	1.39	7.84E-01	NA
chr14:105870531-105870582	5	48.2	*Spry2*	22221 down	1.71	2.46E-02	+ve
chr18:65040023-65040169	5	-49.9	*Nedd4l*	Intron	2.99	1.49E-11	-ve
chr9:63743959-63744150	5	-57.3	*Smad3*	Promoter	1.44	4.04E-01	-ve

Supplemental Figure 5. Correlation of DM CpGs with expression of nearest gene. Related to Figure 6. (A) Correlation (r) of percentage DM of each DMR located within promoters, exons, introns, intergenic and enhancer regions with the log2fold expression of the nearest gene from RNA-Seq data. (B) Location and number of differentially methylated CpGs (# DM CpGs) in each cluster (DMR), along with percentage change in methylation (Δ %), closest gene to DMR and location of DMR within that gene (annotation) fold change from RNA-Seq analysis (FC), p-adj<0.05, and correlation with differential methylation are displayed (r).

A

AP-1 transcription factors

GENE	RNAseq 7d cut FC	P adj value	qPCR 7d cut FC	P adj value
ATF3	19.46	1.41E-077	13.76	0.0037
c-Jun	6.63	1.96E-038	5.097	6.41E-06
Fosl2	5.90	1.26E-33	5.57	5.21E-07
Fos	3.99	1.17E-08	5.43	0.013
Fosl1	3.34	0.15 NS	-1.65	0.085 NS
JunB	2.62	8.94E-05	-4.48	0.029 NS
JunD	-1.16	0.068 NS	1.19	0.53 NS
ATF2	1.00	0.88 NS	-1.02	0.76 NS
FosB	-1.49	0.27 NS	-1.62	0.13 NS

LncRNAs

GENE	RNAseq 7d cut FC	P adj value	qPCR 7d cut FC	P adj value
H19	7.84	1.65E-060	6.4	0.004
Meg3	2.31	1.77E-013	NA	NA
Meg3 transcript 1	N/A	N/A	2.66	0.028
Meg3 transcript 2	N/A	N/A	2.84	2.29E-03
Meg3 transcript 3	N/A	N/A	1.70	0.10 NS
Rian	1.97	4.97E-008	1.95	0.012
Sox2ot	1.78	0.00071	2.64	0.03

Myelin genes

GENE	RNAseq 7d cut FC	P adj value	qPCR 7d cut FC	P adj value
Egr2	-8.6	6.53E-31	-8.00	0.0013
Mpz	-74.65	1.47E-189	-403.33	0.0054

Putative Repair program genes

GENE	RNAseq 7d cut FC	P adj value	qPCR 7d cut FC	P adj value
Rgs8	334.80	1.42E-063	29.05	0.00016
Slc17a8	199.68	2.28E-064	234.51	0.039
Runx2	99.39	1.12E-107	80.70	0.0045
Gpnmb	58.35	9.76E-141	36.58	0.0072
Gadd45a	50.25	1.38E-078	39.95	0.0037
Gria1	41.29	7.16E-066	5.87	0.012
Fgd3	40.88	1.2064E-99	106.21	0.013
Rgs16	14.72	1.16E-040	29.36	0.013
Nr1h4	13.96	4.08E-009	38.00	0.017
Arl4c	12.57	4.77E-027	9.20	0.014
Sox4	9.29	2.56095E-38	8.06	0.0026
Mmp17	7.83	9.33E-034	7.88	0.0011
Adam11	7.21	6.43E-01	4.18	8.0049E-05
C1qb	6.53	6.52E-034	8.39	0.015
Nav2	6.43	6.59E-046	29.01	7.011E-06
Foxd3	4.02	1.73E-012	3.62	0.0003
Socs3	3.18	2.27E-008	6.21	0.00039
Nedd4l	2.99	4.12E-013	4.75	0.037
Cln8	2.36	1.06E-006	4.50	0.025
Mgp	2.26	2.72E-008	10.77	0.0057
Dnmt1	1.74	0.00014	3.91	0.046
Sox2	7.78	9.57E-018	7.47	0.0017

B

Gene	SC % of sum copy no.	Fibroblast % of sum copy no.	Macrophage % of sum copy no.	SC vs. Fibro p adj	SC vs. Macro p adj
Adam11	97.58	2.42	0.00	8.64E-05	6.88E-05
Arl4c	33.37	38.27	28.36	0.719 NS	0.273 NS
Atf2	59.14	23.07	17.79	1.79E-04	2.80E-04
Atf3	71.48	0.55	27.98	0.011	0.028
C1qb	0.30	0.33	99.36	0.843 NS	0.025
Cln8	76.53	5.93	17.55	2.10E-03	3.10E-04
Fgd3	98.14	0.94	0.91	7.15E-03	7.16E-03
Fos	67.07	4.89	28.04	0.019	3.35E-03
FosB	56.16	28.44	15.40	0.111 NS	0.087 NS
Fosl1	85.48	14.06	0.46	8.55E-04	5.32E-03
Fosl2	77.32	15.61	7.07	0.025	0.013
Foxd3	98.77	1.23	0.00	0.027	0.026
Gadd45a	95.07	4.26	0.67	0.040	0.037
Gpnmb	8.47	0.19	91.34	0.293 NS	0.007
Gria1	99.10	0.70	0.20	0.009	0.009
H19	79.01	20.89	0.10	1.67E-03	0.039
c-Jun	88.51	4.31	7.18	0.027	0.025
JunB	10.70	10.82	78.48	0.929 NS	0.023
JunD	58.04	28.82	13.14	0.023	7.24E-03
Meg3t1	45.61	53.59	0.80	0.736 NS	0.045
Meg3t2	45.82	54.14	0.05	0.562 NS	0.030
Meg3t3	41.88	57.65	0.47	0.107 NS	0.004
Mmp17	97.69	2.31	0.01	0.003	0.003
Mob3b	5.79	1.52	92.68	0.099 NS	0.056 NS
Nav2	92.93	3.50	3.57	0.020	0.020
Nedd4l	82.89	4.15	12.96	2.20E-03	1.16E-03
Nrlh4	81.34	17.99	0.67	0.070	0.054
Rian	36.68	63.22	0.10	0.027	0.024
Rgs8	82.17	17.53	0.30	1.97E-04	0.017
Rgs16	75.52	23.62	0.86	6.78E-04	0.011
Runx2	92.99	6.23	0.78	4.38E-06	4.38E-06
Slc17a8	86.47	1.26	12.27	5.36E-03	7.95E-03
Socs3	2.77	2.92	94.30	0.752 NS	0.016
Sox2	99.14	0.77	0.09	0.013	0.013
Sox2ot	98.64	1.19	0.17	0.044	0.044

C

i

TOTAL CELLS (n=3)	SCHWANN CELLS	MACROPHAGES	FIBROBLASTS	OTHER
311	229	41	35	6
	74%	13%	11%	2%

ii

UNTREATED (n=4)	IMMUNOPANNED (n=4)	DESHEATHED (n=4)	IMMUNOPANNED + DESHEATHED (n=4)
48%	62%	80%	89%

Supplemental Table 2. Confirmation of RNA-Seq coding and non-coding RNAs by qPCR in whole nerve and primary cultured cell types and estimates of Schwann cell purity in injured nerves. Related to Figure 1, 2 and 3 and experimental procedures. (A) Fold change for RNA-Seq and qPCR gene expression values (ΔCT values) in seven day cut compared to uncut nerves for AP-1 TF, lncRNAs, Myelin genes and putative repair programme genes. P-adjusted values are given. (B) Relative cell type expression for repair program coding RNAs and lncRNAs in cultured mouse Schwann cells, nerve fibroblasts and activated macrophages displayed as percentage of the sum of qPCR copy number values from 1ug of RNA from each of the three cell types (n=3). Separate p-adjusted values given for statistical comparisons of Schwann cell values to fibroblasts and to macrophages, respectively. RNAs labeled in blue are statistically more highly expressed in Schwann cells than in fibroblasts or macrophages. (C) (i) Cells counts from Electron micrographs at 5000x magnification of seven day cut distal stumps (5mm from cut site; n=3). (ii) Percentage purity of Schwann cells from enzyme dissociated cultures of adult mouse seven day cut nerve, immunopanned, desheathed, immunopanned and desheathed or left untreated (n=4).

A

GENE	PRIMER NAME	PRIMER SEQUENCE
Adam11	**Adam11_For2**	**GGAGAGGTCCGAAAGCCACA**
	Adam11_Rev2	CCGGGATGACGAAACTCACC
Ankrd27	**Ank27_For2**	**TCCTGCCAGTTCGAGTCCTAT**
	Ank27_Rev2	AATGACGACAGCCTTTCCATC
Arl4c	**Arl4c_For1**	**CTGCACATCGTTATGCTGGG**
	Arl4c_Rev1	GGTGTTGAAGCCGATAGTGGG
ATF2	**ATF2_For4**	**ACTCCAACGCCAACAAGATTC**
	ATF2_Rev4	TCTACAACAGAAGGCTCCTCAAT
ATF3	**ATF3_For1**	**CCTTGATAGGTGGGGTGGGA**
	ATF3_Rev1	AACACCAGGCTCTGAAACGG
C1qb	**C1qb_For1**	**GGCTCTGGGAATCCACTGCT**
	C1qb_Rev1	GCCCAGGACACATGGAGAAAA
Canx	**Canx_For2**	**CTTCCAGGGGATAAAGGACTTGT**
	Canx_Rev2	ACATAGGCACCACCACATTCTA
Cln8	**Cln8_For2**	**CAGCTCTCATCGTCCCTGAAT**
	Cln8_Rev2	GGTGATGTGAAACCAACACCA
Dnmt1	**Dnmt1_Fw1**	**CTAGTTCCGTGGCTACGAGG**
	Dnmt1_Rev1	AGTTCCCCTCTTCCGACTCT
Egr2	**Egr2_For2**	**CCGTATCCGAGTAGCTTCGC**
	Egr2_Rev2	TCAATGGAGAATTTGCCCATGT
Fgd3	**Fgd3_For2**	**CACTCAGGACCGACACCTCT**
	Fgd3_Rev2	CAGGCTGCGTTTGGTTTCAC
Fos	**cFos_For3**	**GGTTTCAACGCCGACTACGA**
	cFos_Rev3	GTTGGCACTAGAGACGGACAG
FosB	**FosB_For2**	**ACTGGAGAGAAAGTTTTGTGGGT**
	FosB_Rev2	AGCGTCCCAAGAAATGAGGG
Fosl1	**Fosl1_For1**	**GCATGTACCGAGACTACGGGGAA**
	Fosl1_Rev1	TGCACCATCCAGTGCAGTTCCT
Fosl2	**Fosl2_For3**	**CACGCTCACATCCCTACAGTC**
	Fosl2_Rev3	CCGGATTCGACGCTTCTCCT
Foxd3	**Foxd3_Fw1**	**GACCCCGAACAAGCCCAAG**
	Foxd3_Rev1	GAAAACGGTTGCTGATGAACTC
Gadd45a	**Gadd45a_Fw1**	**CTGCAGAGCAGAAGACCGAA**
	Gadd45a_Rev1	GGGTCTACGTTGAGCAGCTT
GAPDH	**GAPDH_Fw3**	**GGGTGTGAACCAGAGAAA**
	GAPDH_Rev3	GTCTTCTGGGTGGCAGTGAT
GPNMB	**GPNMB_For1**	**ATTACGTGGCTGGTCTTCGG**
	GPNMB_Rev1	CCTTCTGGCATCTGGGGAAC
GRIA1	**GRIA1_For1**	**AAGGGGAATGTGGAAGCAAGG**
	GRIA1_Rev1	CGACTCGCTACGGGATTTGT
H19	**H19_For1**	**GCTCTGGCAAAGTCCCAAGT**
	H19_Rev1	AGTGCCTCATGGGAATGGTG
c-Jun	**cJun_For1**	**CCTTCTACGACGATGCCCTC**
	cJun_Rev1	GGTTCAAGGTCATGCTCTGTTT
JunB	**JunB_Fw4**	**TCACGACGACTCTTACGCAG**
	JunB_Rev4	CCTTGAGACCCCGATAGGGA

GENE	PRIMER NAME	PRIMER SEQUENCE
JunD	**JunD_For2**	**CACGCAAGAACGCATCAAGG**
	JunD_Rev2	GTTGACGTGGCTGAGGACTT
Meg3t1	**Meg3t1_For1**	**GTCCACGTTTTCGTTGCTCC**
	Meg3t1_Rev1	ATGCTGGGAAAGCTGGTTGT
Meg3t2	**Meg3t2_For2**	**GTCCACGTTTTCGTTGCTCC**
	Meg3t2_Rev2	ATGCTGGGAAAGCTGGTTGT
Meg3t3	**Meg3t3_For2**	**ACATCATCGGCTCACACCAG**
	Meg3t3_Rev2	CAGGCAACTTGAGGGAGTGG
MMP17	**MMP17_For3**	**AGTGTGGACGCCGTGTATG**
	MMP17_Rev3	GGAGGCTGAAGTCGGAGACA
MPZ	**MPZ_For1**	**CGGACAGGGAAATCTATGGTGC**
	MPZ_Rev1	TGGTAGCGCCAGGTAAAAGAG
Nav2	**Nav2_For2**	**AGCCTGCTCTGGAGGAACTCA**
	Nav2_Rev2	CTCAGTGGTGACGTTGGTGTC
Nedd4l	**Nedd4l_For1**	**GTCCGGCTGTTCCGTACTC**
	Nedd4l_Rev1	AGGCCATAGTAGGGGTTAAACAT
Nr1h4	**Nr1h4_For**	**GGCAGAATCTGGATTTGGAATCG**
	Nr1h4_Rev	GCCCAGGTTGGAATAGTAAGACG
Rgs16	**Rgs16For1**	**TCCAGGGCTCACCACATCTT**
	Rgs16Rev1	TCTCCATCAATGTGCGGGTC
Rgs8	**Rgs8_For4**	**GCAGGAACAAAGGCATGAGGA**
	Rgs8_Rev4	TGCTTCTTCCGTGGAGAGTCT
Rian	**Rian_For2**	**GACTCATCCTGACGTGCCAA**
	Rian_Rev2	TTCCCTTACCCACACAGGGG
Runx2	**Runx2_For1**	**CACCTCTGACTTCTGCCTCTG**
	Runx2_Rev1	CTGGGTAGTGCATTCGTGGG
Slc17a8	Slc_Rev1	AGCTGACAAGAGACCCACCT
	**Slc_For2**	**CCTATGCTGGGGCAGTTGTT**
Socs3	**Socs3_Fw1**	**GCGGGCACCTTTCTTATCC**
	Socs3_Rev1	CTGGAGGCGGCATGTAGTG
Sox2	**Sox2_For1**	**TCGCAGGGAGTTCGCAAAAG**
	Sox2_Rev1	ACCCAGCAAGAACCCTTTCC
Sox2ot	**Sox2ot_For2**	**GAACGGGCACACATCAAGCA**
	Sox2ot_Rev2	GCCCCCTATACAACACTGCC
Sox4	**Sox4_For2**	**CCAGTTCTTGCACGCTGTTTC**
	Sox4_Rev2	ATGTATGTTTCTCCCTCCCTCCT

B

Primer Name	Product No.	Target Sequence 5'-3'
hsa-miR-17-5p	204771	CAAAGUGCUUACAGUGCAGGUAG
mmu-miR-21a-3p	205400	CAACAGCAGUCGAUGGGCUGUC
mmu-miR-34a-5p	204486	UGGCAGUGUCUUAGCUGGUUGU
mmu-miR-34b-5p	205075	AGGCAGUGUAAUUAGCUGAUUGU
mmu-miR-34c-5p	202017	AGGCAGUGUAGUUAGCUGAUUGC
hsa-miR-92b-3p	204383	UAUUGCACUCGUCCCGGCCUCC
hsa-miR-96-5p	204417	UUUGGCACUAGCACAUUUUUGCU
hsa-miR-124-3p	204319	UAAGGCACGCGGUGAAUGCC
hsa-miR-132-3p	204129	UAACAGUCUACAGCCAUGGUCG
hsa-miR-142-3p	204291	UGUAGUGUUUCCUACUUUAUGGA
hsa-miR-183-3p	204652	UAUGGCACUGGUAGAAUUCACU
hsa-miR-204-5p	205708	UUCCCUUUGUCAUCCUAUGCCU
hsa-miR-362-5p	205073	AAUCCUUGGAACCUAGGUGUGAAU
hsa-miR-383-5p	205000	AGAUCAGAAGGUGACUGUGGCU
hsa-miR-598-3p	205045	UACGUCAUCGUCGUCAUCGUUA

C

Chr	Str	Start	End	Gene	Pr	Sequence
1	+ve	8858 7086	8858 8175	*Arl4c*	F	**TTTTTTTGAAAATGGAGGAAATTATAGA**
					R	AAAATAAACCCAAATCCTCACACATAC
4	-ve	3509 5999	3509 7009	*Mob3b*	F	**ATGAGTTAGGTGTGATTGGGTTAT**
					R	AACCCAACTAAAAACTCAATATAAAATT
8	+ve	1489 0878	1489 2177	*Cln8*	F	**TTAGGGGATAGGGTGTGTTAGTTTAGAG**
					R	ACAACAAAATCCACTTCCTCAACC
10	-ve	8945 2630	8945 3668	*Nr1h4*	F	**GAGATAAAGGAGGGTGGAGATTAAATAAAT**
					R	AAAAACCCCATTACAACCAAAAAC
18	-ve	6503 9524	6504 0670	*Nedd4l*	F	**GAGAGTTTTTTTAAGATTATTGGGATTA**
					R	ACCTTCCTTTAAACTACCTTCAAATACT

Supplemental Table 6. List of primers for quantitative PCR and bisulfite PCR. Related to Figure 2 to 6 and experimental procedures. (A) Primers for qPCR (B) miRNA qPCR and (C) Primers for bisulfite PCR.

2. Supplemental Experimental Procedures

Cell culture preparation

Neonatal mouse Schwann cell culture and cell expansion was performed as previously described. Cultures were consistently >95% pure judged by 04 and S100b (Arthur-Farraj et al., 2011; Stevens et al., 1998). Adult seven day cut nerves were desheathed and dissociated with enzyme cocktail (Woodhoo et al., 2004) for 24 hours before plating on PLL/laminin coverslips plates in Defined medium (Arthur-Farraj et al., 2011) with 0.5% horse serum (Invitrogen, UK). Adult peripheral nerve fibroblasts were obtained from seven day cut nerves, which were not desheathed and were then dissociated in enzyme cocktail for 24 hours before being plated directly onto uncoated tissue culture dishes in DMEM 10% foetal calf serum (Perbio, UK) and expanded for 14 days, re-plating three times to remove Schwann cells. Cultures were >99% pure judged by S100b negative immuno-staining. Bone marrow derived macrophage cultures were prepared as described previously (Behmoares et al., 2010).

Assessment of Schwann cell purity in nerve samples for sequencing

The uncut nerve consists of predominantly of Schwann cells (>90%). Seven days after nerve cut, Schwann cells still make up greater than 70% of the total cell number in the nerve, the majority of the rest being fibroblasts, macrophages and perineural cells. For all DNA sequencing experiments, adult nerves were desheathed, enabling greater than 80% cell purity in cut nerve samples, when tested in cell dissociation experiments. For RNA sequencing experiments, adult nerves were not desheathed as this compromised RNA integrity (Table S2C).

Quantitative RT-PCR

cDNA was synthesized, using 1µg of total RNA per sample and iScript cDNA synthesis kit (BioRad) following manufacturers instruction. MiRCURY LNA Universal RT microRNA PCR kits (Exiqon) were used for microRNA qPCR. For standard qPCR, *Ankrd27* and *Canx* were used as housekeeping genes. All primers were designed using Primer blast (NCBI)(Ye et al., 2012). Primer sequences are detailed in Table S6A. Micro RNA Primers were commercially deigned (Exiqon). miR191-5p was used as a housekeeping miRNA. For target miRNA sequences see Table S6B Standard curves and fluorescent quantitation PCR were performed on 7900HT Fast Real Time PCR Machine (Applied Biosystems) and cDNA quantified according to the manufacturer's software recommendation.

Quantification of relative RNA expression in specific cultured cell types

In order to compare the relative level of expression of RNAs between different cell types *in vitro*, we performed qPCR, using cDNA made from 1ug of RNA extracted from each of the following cell types: cultured mouse Schwann cells; cultured mouse nerve-derived fibroblasts; and cultured mouse bone marrow-derived macrophages treated with Lipopolysaccharide (LPS). The mean qPCR copy number values (n=3) for each cell type were then added together to achieve a sum total. The mean copy number value for each cell type was then expressed as a percentage of the sum total. Preferential expression of an RNA in a particular cell type was determined by an unpaired two-tailed students t-test with Bonferonni correction for multiple testing (p-adjusted <0.05).

Stranded RNA-seq

Briefly, cytoplasmic and mitochondrial rRNA was removed using biotinylated, target specific oligos combined with Ribo-Zero rRNA removal beads. Following purification, RNA was fragmented using divalent cations under elevated temperature and cleaved fragments were copied onto first strand cDNA using reverse transcriptase and random primers. Next, the second strand was synthesised using DNA Polymerase I and RNase H. cDNA 3' ends were adenylated and indexed adapters ligated followed by 10 cycles of library amplification. Finally, the libraries were purified using AMPue XP Beads (Beckman Coulter) and quality was evaluated using Agilent 2100 Bioanalyzer. Three libraries were run on a single lane of the HiSeq 2000 platform (Illumina) to generate 100bp paired-end reads.

Small RNA Seq

Briefly, 3` and 5` indexed adapters were ligated to 1mg of total RNA and the product of this reaction was RT-PCR amplified. Small RNA library was size selected using Pippin Prep elution system (Sage Science). The quality of the libraries was evaluated using Agilent 2100 Bioanalyzer. All the libraries were pooled together and run on two lanes of the HiSeq 2000 platform to generate 50bp single-end reads.

Bisulfite PCR

Primers for bisulfite PCR were designed using MethPrimer (Li and Dahiya, 2002) and sequences are listed in Table S6C. Bisulfite PCR was performed using PfuTurbo Cx hotstart DNA polymerase (Agilent) according to manufacturers guidelines. PCR conditions used 95°C for 2 min; 95°C for 30s, 52°C-62°C for 30s and 72°C for 1min for 40 cycles; then 72°C for 10min.

Analysis of CpG dinucleotide methylation using Bisulfite-Sanger sequencing

Genomic DNA was bisulfite treated using MethylCode ™ bisulfite conversion kit (Thermofisher). Bisulfite PCR was performed and PCR products were then purified and sent for Sanger sequencing. Sequences were analysed using Sequencher® version 5.4.1 sequence analysis software (Gene Codes).

Identification of miRNA sequences present in samples

Illumina adapters were clipped from the 3' end and sequences shorter than 18 nucleotides were discarded. MirDeep2 (v.2.0.0.5) (Friedlander et al., 2012) was used to determine the presence and quantity of miRNAs based on mouse precursor sequences and mature sequences from mouse and rat with miRBase release 19 (Kozomara and Griffiths-Jones, 2011).

Identification of transcriptionally active sequences in samples

RNA-Seq reads were aligned with Tophat splice junction mapper (v2.0.8) (Trapnell et al., 2009) and short read mapper Bowtie (v2.1.0) (Langmead and Salzberg, 2012) using the stranded parameter (fr-firststrand) against the UCSC Mouse genome reference sequence assembly (GCRm38/mm10) and the Ensembl transcript annotations (version GRCm38.73). Gene based read counts were obtained using HTSeq count module (v0.5.4p3) (Anders et al., 2015).

Identification of Enhancer and their association to genes

Putative enhancers were defined by genomic locations derived from Encode and the UCSC browser (ENCODE PMID:22763441 and UCSC PMID: 25428374). Using GREAT (http://bejerano.stanford.edu/great/public/html/index.php) the DM CpGs overlapping enhancers that were associated with a gene were identified using basal plus extension default parameters, which search 5kb upstream of a TSS, 1kb downstream plus distal extension up to 1 Mb from the TSS.

Mapping Rat enhancers to genomic loci in mouse

Rat sciatic nerve H3K27ac Chip peaks were obtained from NCBI GEO under accession number GSE64103. Of the 31,894 active enhancer regions identified in the rn5 genome, 2,529 of these loci map to the mm10 genome using UCSC liftOver in conjunction with the rn5ToMm10.over.chain file.

Assessing DM CpGs within topological associated domains of myelin-associated genes.

Topological associated domains for the mm9 genome were obtained from the Mouse Encode project (http://chromosome.sdsc.edu/mouse/hi-c/download.html) (Pope et al., 2014). The mm9 co-ordinates were converted to mm10 using liftOver. The TAD domains were between 0.8 and 1.9 Mb in size. The total number of DM CpGs and the average DM CpG % were calculated within the TAD that contained the following myelin-associated genes studied by (Varela-Rey et al., 2014); *Nab1*, *Cyp51*, *Slc6a13*, *Prx*, *Lgi4*, *Slc27a1*, *Egr2*, *Kng1*, *Cd9*, *Cebpa*, *Pmp22*, *Nfatc1* and *Cnp*.

De novo motif searching around DM CpG loci

We performed a de novo motif search using findMotifsGenome.pl which is part of the Homer package. We searched for enrichment of motifs of 6, 8, 10, 12, 14 and 16 bp in length within a 40bp window centered on the 853 DM CpGs identified in the mm10 genome.

Differential Expression analysis of RNA-Seq and small RNA-Seq data Differential expression (DE) analysis was performed using DESeq Bioconductor package (Anders and Huber, 2010). Independent filtering was applied to remove the genes in the lowest 40% quantile before statistical testing was employed. The empirical dispersion values for each gene estimated using a conservative approach (sharing mode='maximum'). Raw p values were adjusted for multiple testing with the Benjamini-Hochberg procedure (Benjamini and Hochberg, 1995). Genes or miRNAs with adjusted p-value of 0.05 or less were termed as differentially expressed and are listed in Table S1 and S5 respectively.

Comparison of seven day cut microarray to RNA-Seq datasets

There are 6009 genes that are DE in the uncut vs seven day cut microarray data with p-adj < 0.05, 4999 of which have ensemble IDs (Arthur-Farraj et al., 2012). This is compared to 3,176 DE RNAs from our uncut vs seven day cut RNA-Seq data. However, when we analyse DE genes (p-adj<0.05) with a fold

change >2 in the microarray, 84% (267/317) of downregulated genes and 77% of upregulated genes (326/422) were similarly regulated in our RNA-Seq dataset.

Enrichment Analysis of KEGG Pathways, Gene Ontologies and Protein Families
Enrichment of KEGG Pathways and Biological Processes for the 3331 DEG was calculated using DAVID (v6.8) (Dennis et al., 2003) with a background list of 23,121 genes identified in this study. Raw p-values were adjusted for multiple testing using the Benjamini-Hochberg procedure (Benjamini and Hochberg, 1995) and were deemed significant if the p-adj value was < 0.01.

mRNA-miRNA-TF Target predictions
A miRNA-mRNA target was accepted if at least two of the six programs employed on the miRWalk webserver (miRanda, miRDB, miRWalk, Targetscan, RNA22 and PITA) predicted a significant match ($p<0.05$), with a minimum seed length of seven within the 3'UTR region of the longest transcript. The predicted targets were compared against the list of DE genes identified between uncut and seven day cut nerves and genes common to both were tested for enrichment of pathways and gene ontologies. The miRNA gene target enrichment analysis using DAVID was set at p-adjusted value < 0.05. Magia^2 (Bisognin et al., 2012) was used to reconstruct a post-transcriptional regulatory network and transcription factors (TF) that regulate miRNA were determined. Both miRNA and RNA-Seq expression data was uploaded to Magia^2 (Bisognin et al., 2012) website and the Spearman correlation method alongside experimentally validated TF-miRNA interactions reported in mirGene2.0 (Friard et al., 2010) and Transmir (Wang et al., 2010) were used to determine the relationships between TFs and miRNAs. The following target predictors with mean thesholds were used to determine TF, miRNA, mRNA interactions; mirDB (threshold: 63), Microcos (threshold: 16.3), RNA22 (threshold: -27.5), DIANA microT (threshold 2.0), TargetScan (threshold: 0.7), Pita (threshold 0.2), PicTar (threshold: 3.8) and microrna.org (threshold: -0.3). P values were adjusted for multiple testing using FDR and the subsequent p-adjusted value was deemed significant if less than 0.10.

Enrichment analysis of EMT genes
A list of human EMT genes was obtained from the dbEMT database (Zhao et al., 2015). Mouse orthologs of the Ensembl listed EMT genes were identified (Ensembl v. 85). Of the 326 of the mouse to human orthologous EMT genes, 111 were significantly differentially expressed between cut and uncut nerve. A fishers exact test was performed to determine enrichment. No multiple correction was applied.
There were 20 mature miRNAs listed in the dbEMT database, of which mouse ortholgous miRNAs were identified through miRBase. Of the 17 mature miRNAs present in mouse nerve tissue, when taking into account the 3' and 5' arm of the miRNAs, there were a total of 29 miRNAs expressed in the mouse nerve. There were 14 of these miRNAs DE between cut and cut day three, 15 DE between uncut and cut at day seven, and 11 DE miRNAs between cut day three and cut day seven. A fishers exact test was performed to determine enrichment. No multiple correction was applied.

Identifying novel lncRNAs
To identify novel lncRNAs, cut and uncut bam file replicates were merged using SAMtools (Li et al., 2009) and RNA-Seq alignments were assembled into potential transcripts with StringTie (Pertea et al., 2015) with a minimum isoform fraction of 0.01. Uncut and cut GTF files were merged with Cuffcompare and transcripts were annotated and divided into those that had known Ensembl gene IDs and those that were novel. The protein coding potential of the novel transcripts was determined using Cpat (Wang et al., 2013), where a coding probability (CP) of 0.44 and a minimum length of 200bp was used to determine putative lncRNAs. To remove truncated transcripts resulting from post transcriptional modifications or degradation of RNA, additional quality filters were applied where lncRNAs were accepted if they had > 10 reads in one of the six samples and were expressed (reads > 0) in at least 3 samples. Lift over was used to convert the FANTOM5 mouse CAGE peak data (Forrest et al., 2014) from mm9 to mm10 and a 500 bp window +/- TSS of novel putative lncRNA was intersected these peaks to identify a set of high confidence lncRNAs (Fig. S2).

Detecting Differentially Methylated Bases
Illumina adapters were removed using paired option in trim_galore (v0.2.5) and reads with quality Phred score cut-off < 20 were removed. The overall quality of the sequence data was assessed for suitability using FastQC (v0.9.4)(www.bioinformatics.babraham.ac.uk/projects/fastqc). BSMAP (v2.74) (Xi and Li, 2009) was used to align reads to the mouse genome with quality threshold cut-off at 20, max mismatches at 8% and mapping strand information set to 0 where PE sequencing maps read1 to ++ and -+ strand and read2 to +- and -- strand. Reads were sorted, duplicates marked and removed, bam file indexed and lanes merged using PICARD tools (v1.107)(http://picard.sourceforge.net).

Methylation ratios were extracted from BSMAP mapping files using methratio.py from BSMAP (v2.74) (Xi and Li, 2009) where the minimum sequencing depth for reported loci was 10X and CpG methylation ratios were combined on both strands. Differential methylation was calculated using methylKit (Akalin et al., 2012) which calculates the p-values using logistic regression and these are subsequently adjusted to p-adjusted values using the sliding linear model (SLIM) method (Wang et al., 2011). Differentially methylated bases were then determined if the p-adjusted value was less than 0.05 and the percentage difference in methylation between samples was greater than 20%.

3. Supplemental References

Akalin, A., Kormaksson, M., Li, S., Garrett-Bakelman, F.E., Figueroa, M.E., Melnick, A., and Mason, C.E. (2012). methylKit: a comprehensive R package for the analysis of genome-wide DNA methylation profiles. Genome Biol. *13*, R87.

Anders, S., and Huber, W. (2010). Differential expression analysis for sequence count data. Genome Biol. *11*, R106.

Anders, S., Theodor, P., and Huber, W. (2015). HTSeq - A Python framework to work with high-throughput sequencing data. Bioinformatics, *31*: 166-169.

Arthur-Farraj, P., Wanek, K., Hantke, J., Davis, C.M., Jayakar, A., Parkinson, D.B., Mirsky, R., and Jessen, K.R. (2011). Mouse schwann cells need both NRG1 and cyclic AMP to myelinate. Glia *59*, 720–733.

Behmoares, J., Smith, J., D'Souza, Z., Bhangal, G., Chawanasuntoropoj, R., Tam, F.W., Pusey, C.D., Aitman, T.J., and Cook, H.T (2010). Genetic loci modulate macrophage activity and glomerular damage in experimental glomerulonephritis. J. Am. Soc. Nephrol. *21*, 1136-1144.

Benjamini, Y., and Hochberg, Y. (1995). Controlling the False Discovery Rate - a Practical and Powerful Approach to Multiple Testing. J. Roy. Stat. Soc. B. *57*, 289-300.

Bisognin, A., Sales, G., Coppe, A., Bortoluzzi, S., and Romualdi, C. (2012). MAGIA(2): from miRNA and genes expression data integrative analysis to microRNA-transcription factor mixed regulatory circuits (2012 update). Nuc. Acids Res. *40*, W13-21.

Dennis, G., Jr., Sherman, B.T., Hosack, D.A., Yang, J., Gao, W., Lane, H.C., and Lempicki, R.A. (2003). DAVID: Database for Annotation, Visualization, and Integrated Discovery. Genome Biol. *4*, P3.

Forrest, A.R., Kawaji, H., Rehli, M., Baillie, J.K., de Hoon, M.J., Haberle, V., Lassmann, T., Kulakovskiy, I.V., Lizio, M., Itoh, M. FANTOM Consortium and the RIKEN PMI and CLST (DGT). (2014). A promoter level mammalian expression atlas. Nature. *507*, 462-470.

Friard, O., Re, A., Taverna, D., De Bortoli, M., and Cora, D. (2010). CircuitsDB: a database of mixed microRNA/transcription factor feed-forward regulatory circuits in human and mouse. BMC bioinformatics *11*, 435.

Friedlander, M.R., Mackowiak, S.D., Li, N., Chen, W., and Rajewsky, N. (2012). miRDeep2 accurately identifies known and hundreds of novel microRNA genes in seven animal clades. Nuc. Acids Res. *40*, 37-52.

Kozomara, A., and Griffiths-Jones, S. (2011). miRBase: integrating microRNA annotation and deep-sequencing data. Nucleic Acids Res. *39*, D152-157.
Krueger, F.

Langmead, B., and Salzberg, S.L. (2012). Fast gapped-read alignment with Bowtie 2. Nat. Methods *9*, 357-359.

Li, H., Handsaker, B., Wysoker, A., Fennell, T., Ruan, J., Homer, N., Marth, G., Abecasis, G., Durbin, R. & Genome Project Data Processing. (2009). The Sequence Alignment/Map format and SAMtools. Bioinformatics. *25***,** 2078-9.

Li, M.C., and Dahiya, R. (2002). MethPrimer: designing primers for methylation PCRs. Bioinformatics. *18*, 1427-1431.

Pertea, M., Pertea, G. M., Antonescu, C. M., Chang, T. C., Mendell, J. T. & Salzberg, S. L. (2015). StringTie enables improved reconstruction of a transcriptome from RNA-seq reads. Nat. Biotechnol. *33***,** 290-295.

Pope, B.D., Ryba, T., Dileep, V., Yue, F., Wu, W., Denas, O., Vera, D.L., Wang, Y., Hansen, R.S., Canfield, T.K., et al. (2014). Topologically associating domains are stable units of replication-timing regulation. Nature *515*, 402-405.

Stevens, B., Tanner, S., and Fields, R.D. (1998). Control of myelination by specific patterns of neural impulses. J. Neurosci. *18*, 9303-9311.

Trapnell, C., Pachter, L., and Salzberg, S.L. (2009). TopHat: discovering splice junctions with RNA-Seq. Bioinformatics *25*, 1105-1111.

Wang, H.Q., Tuominen, L.K., and Tsai, C.J. (2011). SLIM: a sliding linear model for estimating the proportion of true null hypotheses in datasets with dependence structures. Bioinformatics *27*, 225-231.

Wang, L., Park, H. J., Dasari, S., Wang, S., Kocher, J. P. & Li, W. (2013). CPAT: Coding-Potential Assessment Tool using an alignment-free logistic regression model. Nucleic Acids Res. *41***,** e74.

Woodhoo, A., Dean, C.H., Droggiti, A., Mirsky, R., and Jessen, K.R. The trunk neural crest and its early glial derivatives: a study of survival responses, developmental schedules and autocrine mechanisms. (2004). Mol. Cell Neurosci. *25*, 30-41.

Xi, Y., and Li, W. (2009). BSMAP: whole genome bisulfite sequence MAPping program. BMC Bioinformatics *10*, 232.

Ye, J., Coulouris, G., Zaretskaya, I., Cutcutache, I., Rozen, S., Madden, T. (2012). Primer-BLAST: A tool to design target-specific primers for polymerase chain reaction. BMC Bioinformatics *13*, 134.
